# Multicomponent Synthesis of Polyphenols and Their In Vitro Evaluation as Potential β-Amyloid Aggregation Inhibitors

**DOI:** 10.3390/molecules24142636

**Published:** 2019-07-19

**Authors:** Denise Galante, Luca Banfi, Giulia Baruzzo, Andrea Basso, Cristina D’Arrigo, Dario Lunaccio, Lisa Moni, Renata Riva, Chiara Lambruschini

**Affiliations:** 1 Istituto per lo Studio delle Macromolecole, Consiglio Nazionale delle Ricerche, via De Marini 6, 16149 Genova, Italy; 2 Department of Chemistry and Industrial Chemistry, University of Genova, via Dodecaneso 31, 16146 Genova, Italy; 3 Department of Pharmacy, Università di Genova, viale Cembrano 4, 16147 Genova, Italy

**Keywords:** multicomponent reactions, polyphenols, β-amyloid proteins, Alzheimer’s disease, Ugi reaction, β-lactams

## Abstract

While plant polyphenols possess a variety of biological properties, exploration of chemical diversity around them is still problematic. Here, an example of application of the Ugi multicomponent reaction to the combinatorial assembly of artificial, yet “natural-like”, polyphenols is presented. The synthesized compounds represent a second-generation library directed to the inhibition of β-amyloid protein aggregation. Chiral enantiopure compounds, and polyphenol-β-lactam hybrids have been prepared too. The biochemical assays have highlighted the importance of the key pharmacophores in these compounds. A lead for inhibition of aggregation of truncated protein AβpE3-42 was selected.

## 1. Introduction

Natural polyphenols of plant origin are important elements of our diet and, for this reason, their biological properties have been thoroughly studied [1,2]. Their most renowned characteristic is their antioxidant activity, which is believed to play an important role in preventing age-related diseases such as atherosclerosis. Since long ago, plant polyphenols have attracted much interest in the nutraceutical and cosmetic fields. On the other hand, the pharmaceutical industry has remained so far rather detached regarding the investigation of plant polyphenols as possible leads for drug development [3]. The reasons for this lack of interest are not fully clear but may be related to the poor pharmacokinetic properties of natural polyphenols, to their in vivo instability, and to the challenging synthetic modification of the natural members of this family. Actually, only few studies on the preparation of artificial analogues through total synthesis or semi-synthesis have been reported so far [3,4,5,6,7,8,9,10,11,12]. Nevertheless, several reports have pointed out that polyphenols, apart from anti-oxidant properties, may have a variety of other biological effects, such as anti-inflammatory [13], anti-cancer [12,14,15], anti-microbial [16], and anti-hyperglycemic [17] activity. Last but not least, some natural polyphenols have been demonstrated to be able to inhibit β-amyloid aggregation, thus being promising for the prevention of the Alzheimer’s disease [18,19,20,21].

**Figure 1:**
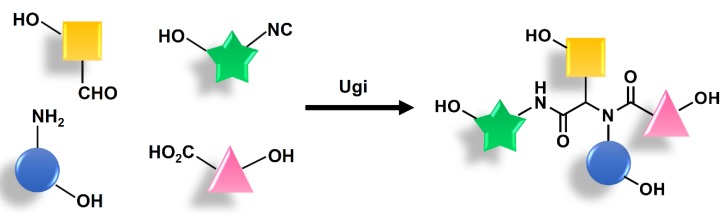
General strategy for assembling artificial polyphenols from simple phenolic building blocks through the Ugi reaction.

We reasoned that a “natural fragment-based approach” [22] to the combinatorial synthesis of artificial polyphenols starting from small, phenolic, building blocks, would allow a more systematic exploration of their chemical space, allowing to select possible hits for drug discovery, endowed with modulable pharmacodynamic and pharmacokinetic properties. In particular, the Ugi multi-component reaction seemed particularly well suited for this approach, since it leads to mixed polyphenol-peptidomimetic structures and allows rigidification through post-MCR cyclization steps [23,24]. A similar approach was recently used by Ismaili et al. [7], who developed a multi-target lead for Alzheimer’s disease by joining ferulic acid to an acetylcholine esterase inhibitor, lipoic acid and a melatonin analogue. However, in Ismaili’s work, only one of the four components used as inputs in the Ugi reaction was a phenol, and the final product was not indeed polyphenols. On the contrary, in our plan, depicted in Figure 1, up to four of the components would be of phenolic nature, thus leading to true artificial, polyphenols of general formula **1**. Our preliminary results using this strategy were reported in a previous paper [25]. Now, also on the basis of the biochemical properties of the first-generation library, we have extended exploration of the chemical space of these polyphenols, preparing new chemical entities, also exploiting components that are chiral enantiopure or that allow post-MCR cyclization.

## 2. Results and Discussion

### 2.1. Synthesis

**Scheme 1:**
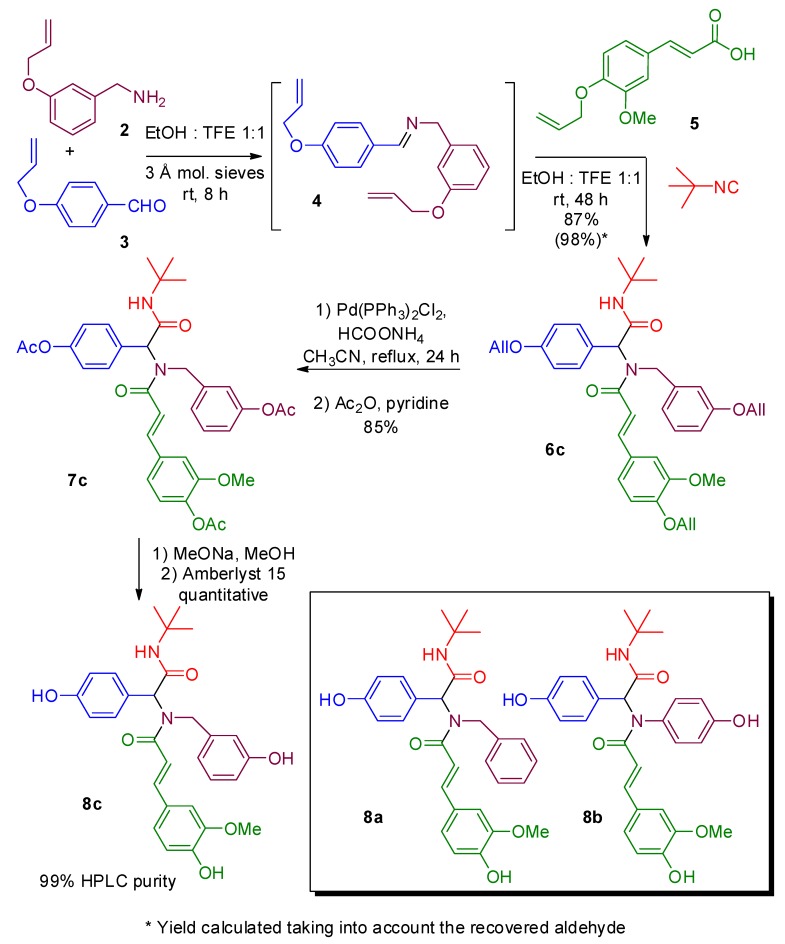
Representative example of the synthesis of artificial polyphenols.

Scheme 1 shows a specific example of the general synthetic strategy used for the preparation of polyphenols. This strategy was optimized also on the basis of the outcomes of our initial preliminary work. The need, in particular for in vivo experiments, of high purity final polyphenols **8**, prompted us to prepare compounds **8** through a high yielding solvolysis of polyacetates **7**. In this way, the purification is very simple, and we can avoid an extractive and chromatographic purification of the polyphenols, which, in some cases, although not always, gave insufficiently pure compounds. On the other hand, the acetate protecting group was found to be not fully stable under the conditions of the Ugi reaction. Thus, our standard, optimized, procedure involved the use of phenolic building blocks protected as allyl ethers. The Ugi reaction gave best results when preformation of the imine was implemented and CF_3_CH_2_OH (TFE)/ethanol mixture was used as the solvent. Removal of the allyl protecting groups was best performed with a minimum amount of Pd catalyst and ammonium formate as the scavenger, followed by immediate acetylation. Chromatographic purification afforded acetates **7**, which were obtained in high purity and fully characterized at this level. Finally, deacetylation was performed with a protocol that completely avoided both extractive and chromatographic purification of the final polyphenols **8**. As shown in Scheme 1, for compound **8c** this procedure was very efficient, and the final HPLC purity was 99%.

**Figure 2:**
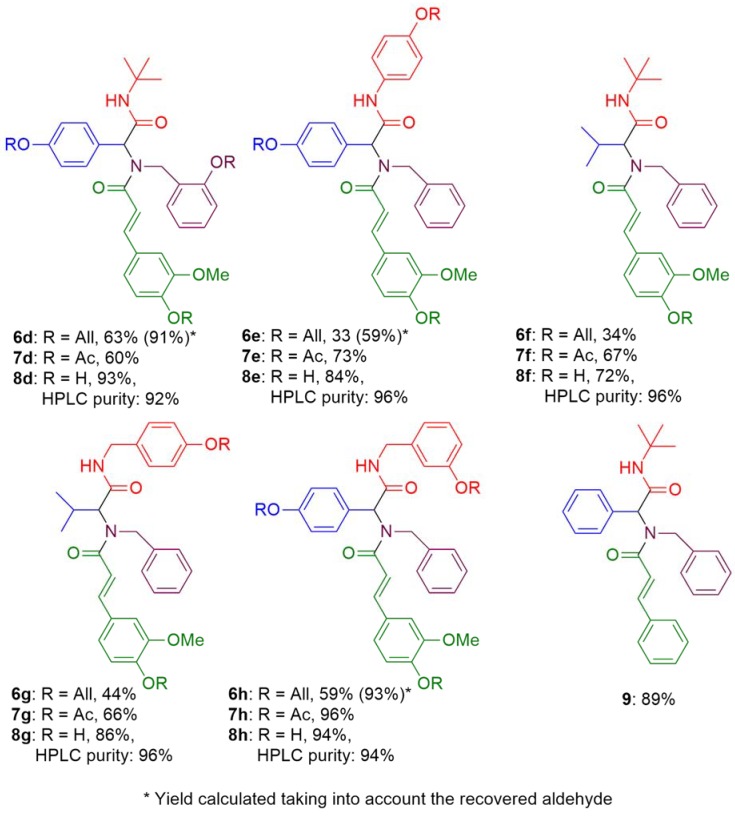
Simple analogues of **8a** prepared.

Other similar polyphenols prepared by us are shown in Figure 2. In designing the new entities to be synthesized, we took as a model the best compounds that emerged from the preliminary studies [25]. In that paper, we tested the polyphenols on two amyloid proteins: Aβ1-42 and AβpE3-42. For the first one, the best compound was found to be **8a**. On the smaller fragment, the best hits contained a residue derived from caffeic acid, but we later found that polyphenols containing the catechol moiety typical of caffeic acid where less stable and, most of all, cytotoxic to neuronal cells [26]. Thus, we decided to keep, as the lead for AβpE3-42, compound **8b**, which was the best among those derived from ferulic acid.

Since the ferulic derived part seemed to be very important, in this study we maintained the carboxylic building block, and varied only the other three components. For example, looking at the structure of **8a**, we replaced benzylamine with a phenol containing benzylamine (see **8c** and **8d**). In **8f** and **8g** we replaced *p*-hydroxybenzaldehyde (that was the aldehyde component for preparation of both **8a** and **8b**) with an aliphatic aldehyde (isobutyraldehyde). In **8e** we replaced *t*-butyl isocyanide with a phenol containing an aromatic isocyanide, whereas for **8g** and **8h** a benzyl isocyanide containing a phenol was used. Finally, we also prepared compound **9**, which is the analogue of **8a**, totally devoid of phenolic groups, in order to check the importance of this moiety for biological activity.

As far as it concerns the overall yields of the various syntheses, the most critical step was found to be the Ugi reaction. In general, we can say that aromatic isocyanides (see **6d**) and aliphatic aldehydes (see **6e** and **6f**) bring about a less efficient multicomponent reaction. The high yield achieved for compound **9** demonstrates that also protected ferulic acid, as the carboxylic component, is not ideal for the Ugi reaction. The decrease in the yield is typically due to sluggish reactions and incomplete conversion. In some instances, we have determined the yield considering the recovered starting aldehyde. However, it should be noted that, since our main goal was to assess the biochemical properties of our polyphenols, the individual syntheses have not been optimized.

**Scheme 2:**
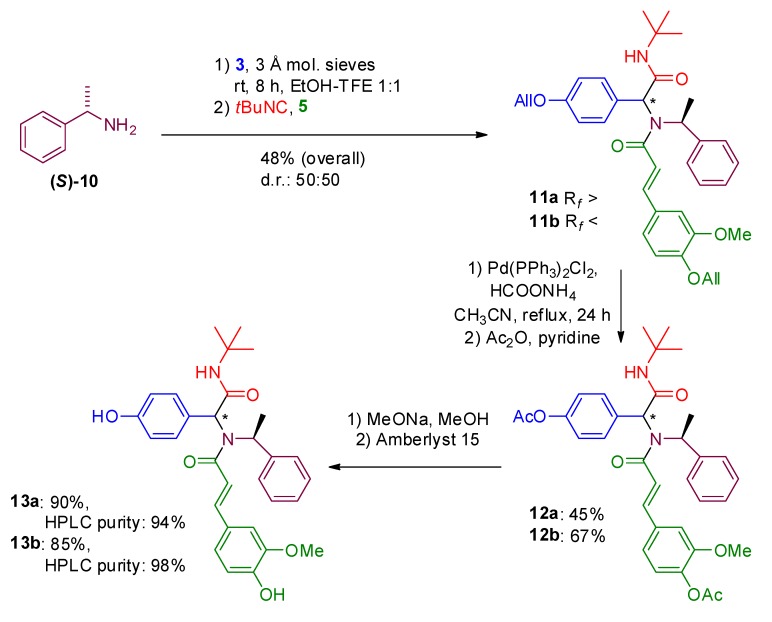
Preparation of enantiopure polyphenols **13a**,**b**.

All the polyphenols prepared in the previous paper and those depicted in Scheme 1 and Figure 2 are racemic. To verify the possible influence of the absolute configuration of the stereogenic center generated during the Ugi reaction, we decided to use, as amine component, enantiopure α-methylbenzylamine **10** (Scheme 2).

As expected, the Ugi reaction was poorly diastereoselective (and slower compared to the ones employing unsubstituted benzylamines). The two diastereomers could be conveniently separated at this stage, and then independently converted into enantiopure polyphenols **13a** and **13b**.

**Scheme 3:**
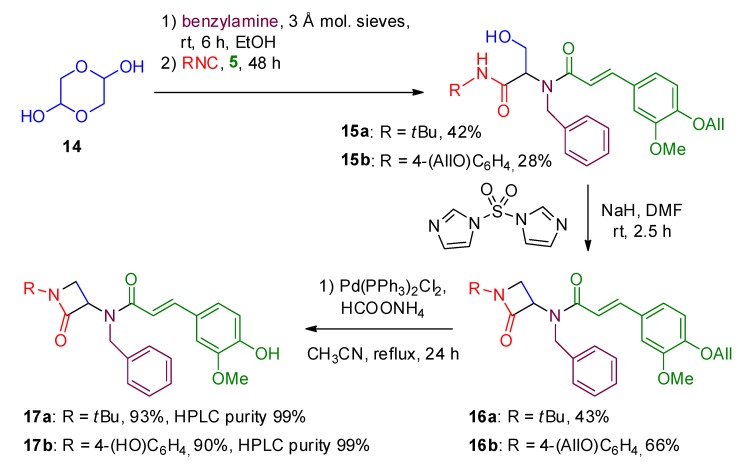
Preparation of β-lactams **17a**,**b**.

Finally, we wanted to prepare some rigidified analogues, by applying a post-MCR cyclization. In a previous work within our group [27], we have noticed that Ugi adducts derived from glycolaldehyde could be cyclized to β-lactams, exploiting the isocyanide derived secondary amide as the nucleophile in an intramolecular S_N_2 process [23]. This interesting reaction was not fully explored, but we reasoned that in the present case it could offer a possibility to obtain polyphenol-β-lactam hybrids as rigidified analogues of **8a** and **8b**. Scheme 3 shows this approach.

Glycolaldehyde dimer **14** has been already used by us and others in Ugi or Ugi-type reactions [27,28,29]. Ugi reactions with **14** are known to proceed only in moderate yields, and, as stated above, protected ferulic acid, like other α,β-unsaturated acids, is not an ideal component for this MCR. Thus, we were not surprised by the low to moderate yields achieved for **15a** and **15b**. Cyclization was then carried out using sulfonyl diimidazole in the presence of NaH. This method, first introduced by Hanessian [30,31], is a variant of the better renown Mitsunobu reaction, and offers advantages in terms of operational simplicity and atom economy [32,33]. Formation of the β-lactams took place quite fast, but it was much cleaner starting with the more acidic aromatic secondary amine **16b**, whereas with the *t*-butyl derivative **16a** several side products were present, lowering the yields.

In this case, we preferred to avoid the final deacetylation. Therefore, after deprotection of the allyl groups, pure final phenols **17a**,**b** were directly obtained by chromatography in highly pure form, being not susceptible to degradation under chromatographic conditions.

### 2.2. Biochemical Assays

Then, we investigated the interaction of our candidates with two β-amyloid peptides, Aβ1-42 and AβpE3-42. While the full-length Aβ1-42 is one of the most abundantly identified in the brain deposits, AβpE3-42 is a peptide *N*-terminal truncated at residue 3 (Glu) and further modified by cyclization of Glu (E) to pyroglutamic acid (pE). These structural modifications are known to increase AβpE3-42 aggregation propensity [34].

First, in order to check that no precipitation of our phenols could occur under the assay conditions, we determined the solubility in phosphate buffer solution (PBS) at pH 7.4 containing 1% of DMSO. This percentage of DMSO does not alter the aggregation of β-amyloids, as demonstrated by control experiments carried out with the solvent alone. This analysis was performed by UV-VIS spectroscopic monitoring of the solutions of the compounds at different concentrations, both at the λ_max_ (at around 330 nm) and at 405 nm to evidence the formation of turbidity. Only compound **8e** was found to be fully soluble in the whole range of concentrations tested (10–500 µM). In this case the absorbance curve at 333 nm was linear and the line at 405 nm flat. For the other compounds bending of the absorbance curve and increase of turbidity started at the concentrations indicated in Table 1, which were anyway far beyond the one used for the subsequent experiments (25 µM). Interestingly, most of the newly synthesized compounds turned out to be more soluble than our previous lead **8a**, and this may represent ad advantage for in vivo tests. The aggregation inhibition experiments were carried out with the thioflavin T methodology. This assay was selected during our previous work as the most helpful in order to predict activity of our polyphenols. We also preliminary tried to investigate the activity of our polyphenols by ANS fluorescence. However, ANS tests were not informative in the case of compounds derived from ferulic acid, since they showed major spectral interferences with ANS. On the other hand, also circular dichroism experiments were not useful, because of interference of 1% DMSO, which was needed to keep our compounds in solution. During our preliminary work [25,26], we have also used electron microscopy and NMR studies, which confirmed the thioflavin data. However, these long studies were carried out only on **8a** or other few selected compounds, and are not suited for a fast selection of the here reported second generation compounds, that was made with thioflavin T.

β-Amyloid aggregation is known to start when a change of the secondary structure from α-helix (in the membrane environment) or coil (in basic environment) to β-sheet conformation takes place [35]. In the presence of thioflavin-T, β-sheet formation results in a strong increase of fluorescence of the solution. Ideally, inhibitors of aggregation should decrease the maximum plateau of fluorescence obtained in the control experiment. Table 1 shows the relative decrease (or increase) of plateau achieved with compounds **8a**,**h**, **9**, **13a**,**b**, **17a**,**b**.

From the results collected we can draw some useful information. First, as long as we consider Aβ1-42, the lead compound **8a** remains the best one. In particular, substitution of the *t*-Bu group of the isocyanide with a hydroxybenzyl group is highly deleterious, leading to a complete loss of the inhibitory effect (see entries 8 and 9). Only substitution with a hydroxyphenyl group is accepted (entry 6) although a decrease of activity is observed compared to the lead compound **8a**. Also replacing of *p*-hydroxybenzaldehyde with an aliphatic counterpart is detrimental (entry 7).

For AβpE3-42, as already experienced in our previous work, even small differences seem to have a significant effect. The best compound, among those prepared in this second campaign, is **8c**, which turned out to be slightly superior to our lead **8b**. Moreover, this molecule is one of the few, together with **8b**, that shows acceptable activity towards both proteins. Therefore, we think that it is worth of further investigation through in vivo assays. The difference, compared to **8b**, is the amine component, which is a hydroxybenzyl instead of a hydroxyphenyl group. Also, for AβpE3-42, the compounds derived from *iso*-butyraldehyde did not inhibit aggregation. On the contrary, they seem, especially **8f**, to even favor β-sheet formation.

Among the β-lactams, only the one containing the *p*-hydroxyphenyl group showed some activity (entry 14). It was equally active on both proteins and may be a good starting point for further refinement.

It was interesting to see if the different configuration of the new stereogenic center created during the Ugi MCR could influence activity. The results achieved with diastereomers **13a** and **13b** shows a negligible effect on Aβ1-42, but a significant difference in behavior for AβpE3-42. It is worth noting that **13b**, which has just an additional methyl, displays a higher activity for the truncated peptide than **8a**, but we do not know whether this is due to its enantiomeric purity or for the presence of the methyl group.

Finally, it should be noted that compound **9**, lacking any phenolic group, had no inhibitory activity at all. On the contrary, it seems to favor aggregation of Aβ1-42. Furthermore, compounds **8f** and **17a**, that are just monophenols, behaved poorly, stressing the need for a polyphenolic system. The best candidates found so far (**8a**, **8b**, **8c**) are indeed either diphenols or triphenols.

## 3. Materials and Methods

### 3.1. General Information

NMR spectra (see Supplementary Materials) were recorded on a Gemini 300 Mhz instrument (Varian, Palo Alto, CA, USA) at r.t. in CDCl_3_ or in DMSO-*d*_6_ at 300 MHz (^1^H), and 75 MHz (^13^C), using, as internal standard, TMS (^1^H NMR in CDCl_3_; 0.000 ppm) or the central peak of DMSO (^1^H-NMR: 2.506 ppm; ^13^C-NMR: 39.43 ppm) or the central peak of CDCl_3_ (^13^C in CDCl_3_; 77.02 ppm). Chemical shifts are reported in ppm (δ scale). Peak assignments were made with the aid of gCOSY and gHSQC experiments. In ABX system, the proton A is considered upfield and B downfield. IR spectra were recorded as solid, oil, or foamy samples, with the ATR (attenuated total reflectance) technique. TLC analyses were carried out on silica gel plates and viewed at UV (λ = 254 nm or 360 nm) and developed with Hanessian stain (dipping into a solution of (NH_4_)_4_MoO_4_·4H_2_O (21 g) and Ce(SO_4_)_2_·4H_2_O (1 g) in H_2_SO_4_ (31 mL) and H_2_O (469 mL) and warming). R*_f_* values were measured after an elution of 7–9 cm. HRMS: samples were analyzed with a Synapt G2 QToF mass spectrometer (Waters, Milford, MA, USA). MS signals were acquired from 50 to 1200 *m*/*z* in either ESI positive or negative ionization mode. Column chromatography was done with the “flash” methodology by using 220–400 mesh silica. Petroleum ether (40–60 °C) is abbreviated as PE. All reactions employing dry solvents were carried out under nitrogen. After extractions, the aqueous phases were always re-extracted 2 times with the appropriate organic solvent, and the organic extracts were always dried over Na_2_SO_4_ and filtered before evaporation to dryness.

Due to a tendency to partially degrade, the free phenols (**8c**–**h**, **13a**,**b**, **18a**,**b**) were fully characterized and stored in the acetylated form (**7c**–**h**, **12a**,**b**, **17a**,**b**) and then deprotected shortly before use through procedure B, checking the purity by ^1^H-NMR and HPLC. HPLC analyses were carried out on a HP-1100 system (Agilent, Santa Clara, CA, USA) equipped with a Phenyl C6 reverse phase column (150 × 3 mm, 3 µm) at 25 °C with flow = 0.34 mL/min. Gradient from CH_3_CN/H_2_O 40:60 (time 0) to pure CH_3_CN (time 10). detection was done with a DAD detector at 330 nm. Compounds **2**, **3** and **5** and 4-(allyloxy)phenyl isocyanide were prepared as previously described [25]. Known [36] 2-(allyloxy)benzylamine was prepared as previously described [32].

### 3.2. Syntheses

*(R,S)-(E)-3-(4-(Allyloxy)-3-methoxyphenyl)-N-(3-(allyloxy)benzyl)-N-(1-(4-(allyloxy)phenyl)-2-(tert-butylamino)-2-oxoethyl)acrylamide* (**6c**). A solution of aldehyde **3** (200 mg, 1.23 mmol) in dry trifluoroethanol (TFE, 2.5 mL) and dry ethanol (2.5 mL) was treated with amine **2** (221 mg, 1.35 mmol) and freshly activated powdered 3 Å molecular sieves (62 mg). The suspension was stirred for 8 h at rt. Then, allylated ferulic acid **5** (318 mg, 1.36 mmol) and *tert*-butyl isocyanide (154 µL, 1.36 mmol) were added. After stirring for 48 h at rt, the mixture was diluted with CH_2_Cl_2_/MeOH 1:1 and filtered through a Celite cake. After evaporation of the solvent, the crude was taken up in EtOAc and washed with saturated aqueous NaHCO_3_ to remove excess of 5, and then with brine. Evaporation of the organic phase to dryness, followed by chromatography (PE:EtOAc 7:3) gave pure **6c** as a white foam (667 mg, 87%). Unreacted aldehyde **3** (24 mg) was also recovered. Yield based on unrecovered starting material: 98%. R*_f_* 0.61 (PE/EtOAc 60:40). ^1^H-NMR (CDCl_3_): δ 7.65 (d, *J* = 15.1 Hz, 1 H, C*H*=CHCO), 7.33 (d, *J* = 8.2 Hz, 2 H), 7.19 (broad s, 1 H), 7.08 (t, *J* = 7.8 Hz, 1 H), 6.91 (d, *J* = 7.8 Hz, 1 H), 6.80 (d, *J* = 8.1 Hz, 1 H), 6.78 (d, *J* = 8.2 Hz, 2 H), 6.67 (d, *J* = 8.2 Hz, 1 H), 6.65 (d, *J* = 8.2 Hz, 1 H), 6.54 (d, *J* = 15.1 Hz, 1 H, CH=C*H*CO), 6.52 (s, 1 H), 6.13 (s, 1 H, C*H*N), 6.11–5.89 (m, 3 H, C*H*=CH_2_), 5.63 (s, 1 H, N*H*), 5.44–5.19 (m, 6 H, CH=C*H*_2_), 4.85 (d, *J* = 17.9 Hz, 1 H, C*H*HAr), 4.64 (d, *J* = 17.9 Hz, 1 H, C*H*HAr), 4.60 (d, *J* = 5.4 Hz, 2 H, C*H*_2_CH=CH_2_), 4.48 (d, *J* = 4.9 Hz, 2 H, C*H*_2_CH=CH_2_), 4.36 (d, *J* = 4.2 Hz, 2 H, C*H*_2_CH=CH_2_), 3.78 (s, 3 H, OC*H*_3_), 1.35 (s, 9 H, C(C*H*_3_)_3_). ^13^C-NMR (CDCl_3_): δ 169.3, 168.3 (*C*=O), 158.7, 158.6, 149.5, 149.3, 140.5, 128.4, 127.6 (quat.), 143.2 (*C*H=CHCO), 133.1, 133.0, 132.8 (*C*H=CH_2_), 131.1 (×2), 129.3, 121.9, 118.7, 114.8 (×2), 113.4, 112.8, 112.4, 110.0 (aromatic *C*H), 118.2, 117.8, 117.6 (CH=*C*H_2_), 116.5 (CH=*C*HCO), 69.7, 68.7, 68.6 (*C*H_2_CH=CH_2_), 61.9 (*C*HN), 55.8 (O*C*H_3_), 51.6 (*C*(CH_3_)_3_), 49.5 ((*C*H_2_Ar), 28.7 (*C*(CH_3_)_3_). HRMS: *m*/*z* (ESI+): 625.3270 (M + H^+^). C_38_H_45_N_2_O_6_ requires 625.3278.

*(R,S)-(E)-3-(4-(Acetoxy)-3-methoxyphenyl)-N-(3-(acetoxy)benzyl)-N-(1-(4-(acetoxy)phenyl)-2-(tert-butylamino)-2-oxoethyl)acrylamide* (**7c**). A solution of triallyl derivative **6c** (252 mg, 404 µmol), Pd(PPh_3_)_2_Cl_2_ (13 mg, 18.3 µmol, 0.015 eq. calculated on the number of allyl groups), and ammonium formate (114 mg, 1.81 mmol, 1.5 eq. calculated on the number of allyl groups) in dry CH_3_CN (3.6 mL) in a pressure tube was first flushed with argon and then sealed and heated at 81 °C for 24 h. A tlc showed that the reaction was complete. The resulting dark solution was diluted with EtOAc and saturated aqueous NaHCO_3_. The phases were separated and the aqueous one re-extracted three times with EtOAc. The organic phases were washed with brine and evaporated to dryness. The crude was taken up in pyridine (1.45 mL, 18.0 mmol) and treated with acetic anhydride (1.45 mL, 15.3 mmol) and stirred at rt for 3 h. The solution was diluted with EtOAc (20 mL), water (20 mL), and 2 M aqueous HCl (10 mL), checking that the resulting pH is <2 (otherwise more HCl is added). The phases were separated, and the aqueous one re-extracted three times with EtOAc. The organic extracts were washed with brine, evaporated to dryness and chromatographed (PE/EtOAc 60:40 + 2% EtOH) to give pure **7c** as a white powder (221 mg, 87%). R*_f_* 0.30 (PE/EtOAc 60:40). IR: υ_max_ 3318, 2969, 2936, 1761, 1680, 1647, 1601, 1538, 1505, 1453, 1417, 1365, 1300, 1256, 1189, 1155, 1121, 1079, 1031, 1012, 977, 946, 908, 869, 829, 795, 750, 731, 697, 643 cm^−1^. ^1^H-NMR (CDCl_3_): δ 7.72 (d, *J* = 15.3 Hz, 1 H, C*H*=CHCO), 7.38 (d, *J* = 8.2 Hz, 2 H), 7.17 (t, *J* = 7.8 Hz, 1 H), 7.10–6.79 (m, 7 H), 6.75 (s, 1 H), 6.61 (d, *J* = 15.3 Hz, 1 H, CH=C*H*CO), 6.10 (s, 1 H, C*H*N), 5.70 (s, 1 H, N*H*), 4.91 (d, *J* = 18.0 Hz, 1 H, C*H*HAr), 4.65 (d, *J* = 18.0 Hz, 1 H, C*H*HAr), 3.76 (s, 3 H, OC*H*_3_), 2.29, 2.27, 2.25 (3 s, 3 × 3 H, C*H*_3_CO), 1.36 (s, 9 H, C(C*H*_3_)_3_). ^13^C-NMR (CDCl_3_): δ 169.1, 168.8, 168.7, 168.0 (C=O), 151.2, 150.8, 150.7, 141.0, 140.0, 134.0, 132.5 (quat.), 143.5 (*C*H=CHCO), 130.9 (×2), 129.4, 123.5, 123.1, 121.8 (×2), 120.9, 120.2, 119.5, 111.4 (aromatic *C*H), 118.2 (CH=*C*HCO), 61.8 (*C*HN), 55.8 (O*C*H_3_), 51.8 (*C*(CH_3_)_3_), 49.2 (*C*H_2_Ar), 28.6 (C(*C*H_3_)_3_), 21.1 (×2), 20.6 (*C*H_3_CO). HRMS: *m*/*z* (ESI+): 631.2664 (M + H^+^). C_35_H_39_N_2_O_9_ requires 631.2656.

*(R,S)-(E)-N-(2-(tert-Butylamino)-1-(4-(hydroxy)phenyl)-2-oxoethyl)-N-(3-hydroxybenzyl)-3-(4-hydroxy-3-methoxyphenyl)acrylamide* (**8c**). A 0.2 M solution of MeONa in MeOH is freshly prepared treating MeOH with solid Na (4.6 g per liter of MeOH) under nitrogen. Triacetate **7c** (100 mg, 160 µmol) is treated with such solution (4.8 mL, 920 µmol, 2 eq. for each acetyl group). After stirring for 2 h at rt, the solution is treated with dry Amberlyst^®^ 15 (4.7 mmol/g) (freshly thoroughly washed with dry methanol) so that the resulting pH is around 4–5 (about 200 mg of dry resin). The resin was filtered off, washing with methanol and the resulting filtrate evaporated to dryness, to afford **8c**, pure enough for biochemical assays (80 mg, quantitative). The purity by HPLC (for conditions see the general remarks) was 99%. R*_f_* 0.18 (PE/EtOAc 50:50) ^1^H-NMR (DMSO-*d*_6_, 90 °C): δ 9.07, 8.98, 8.84 (3 broad s, 3 × 1 H, O*H*), 7.39 (d, *J* = 15.3 Hz, 1 H, C*H*=CHCO), 7.33 (s, 1 H, N*H*), 7.11 (d, *J* = 8.4 Hz, 2 H), 6.98 (s, 1 H), 6.94 (d, *J* = 7.5 Hz, 1 H), 6.93 (broad t, 1 H), 6.75 (d, *J* = 8.1 Hz, 1 H), 6.73–6.68 (broad t, 1 H), 6.68 (d, *J* = 8.4 Hz, 2 H), 6.58–6.46 (m, 3 H, CH=C*H*CO and 2 aromatic C*H*), 5.95 (s, 1 H, C*H*N), 4.76 (d, *J* = 16.8 Hz, 1 H, C*H*HAr), 4.47 (d, *J* = 16.8 Hz, 1 H, C*H*HAr), 3.77 (s, 3 H, OC*H*_3_), 1.25 (s, 9 H, C(C*H*_3_)_3_).

*(R,S)-(E)-3-(4-(Allyloxy)-3-methoxyphenyl)-N-(2-(allyloxy)benzyl)-N-(1-(4-(allyloxy)phenyl)-2-(tert-butylamino)-2-oxoethyl)acrylamide* (**6d**). This compound was prepared starting from 194 mg of aldehyde **2**, following the same procedure above described for **6c**. Pure **6d** was obtained after chromatography with PE/EtOAc 70:30 + 2% EtOH. Yield: 471 mg (63%). Also 59 mg of unreacted aldehyde was recovered from the column (yield from unrecovered starting aldehyde = 91%). Slightly yellow foam. R*_f_* 0.36 (PE/EtOAc 70:30). ^1^H-NMR (CDCl_3_): δ 7.67 (d, *J* = 15.3 Hz, 1 H, C*H*=CHCO), 7.29 (d, *J* = 8.2 Hz, 2 H), 7.19 (broad s, 1 H), 7.09 (t, *J* = 7.0 Hz, 1 H), 6.93 (d, *J* = 8.3 Hz, 1 H), 6.85–6.67 (m, 6 H), 6.56 (d, *J* = 15.3 Hz, 1 H, CH=C*H*CO), 6.52 (s, 1 H), 6.12–5.92 (m, 3 H, C*H*=CH_2_), 5.86 (s, 1 H, C*H*N), 5.73 (s, 1 H, N*H*), 5.43–5.23 (m, 6 H, CH=C*H*_2_), 4.86 (d, *J* = 18.4 Hz, 1 H, C*H*HAr), 4.69 (d, *J* = 18.4 Hz, 1 H, C*H*HAr), 4.60 (dt, *J* = 5.4 (d), 1.2 (t) Hz, 2 H, C*H*_2_CH=CH_2_), 4.55–4.40 (m, 4 H, C*H*_2_CH=CH_2_), 3.78 (s, 3 H, OC*H*_3_), 1.33 (s, 9 H, C(C*H*_3_)_3_). ^13^C-NMR (CDCl_3_): δ 169.1, 168.3 (*C*=O), 158.4, 155.0, 149.4, 149.3, 128.6, 127.5, 120.6 (quat.), 143.1 (*C*H=CHCO), 133.2, 133.0, 132.9 (*C*H=CH_2_), 131.0 (×2), 127.9, 127.8, 121.6, 120.6, 114.5 (×2), 112.8, 110.9, 110.6 (aromatic *C*H), 118.2, 117.7, 117.4 (CH=*C*H_2_), 116.5 (CH=*C*HCO), 69.7, 68.7 (×2) (*C*H_2_CH=CH_2_), 63.1 (*C*HN), 55.9 (O*C*H_3_), 51.4 (*C*(CH_3_)_3_), 44.9 ((*C*H_2_Ar), 28.7 (C(*C*H_3_)_3_). HRMS: *m*/*z* (ESI+): 625.3270 (M + H^+^). C_38_H_45_N_2_O_6_ requires 625.3263.

*(R,S)-(E)-3-(4-(Acetoxy)-3-methoxyphenyl)-N-(2-(acetoxy)benzyl)-N-(1-(4-(acetoxy)phenyl)-2-(tert-butylamino)-2-oxoethyl)acrylamide* (**7d**). It was prepared from 438 mg of **6d** following the same procedure employed for **7c**. Pure **7d** was obtained after chromatography (PE/EtOAc 60:40 + 1% EtOH) (267 mg, 60%). White foam. R*_f_* 0.27 (PE/EtOAc 60:40). IR: υ_max_ 3322, 2967, 2930, 1759, 1681, 1649, 1602, 1542, 1506, 1454, 1418, 1367, 1302, 1259, 1190, 1156, 1122, 1093, 1032, 1011, 980, 951, 907, 826, 751, 664, 645 cm^−1^. ^1^H-NMR (CDCl_3_): δ 7.68 (d, *J* = 15.3 Hz, 1 H, C*H*=CHCO), 7.45 (d, *J* = 8.3 Hz 2 H), 7.16 (broad t, 1 H), 7.10–6.85 (m, 8 H), 6.53 (d, *J* = 15.3 Hz, 1 H, CH=C*H*CO), 6.15 (s, 1 H, C*H*N), 5.63 (s, 1 H, N*H*), 4.79 (d, *J* = 18.4 Hz, 1 H, C*H*HAr), 4.56 (d, *J* = 18.4 Hz, 1 H, C*H*HAr), 3.74 (s, 3 H, OC*H*_3_), 2.30, 2.28, 2.27 (3 s, 3 × 3 H, C*H*_3_CO), 1.35 (s, 9 H, C(C*H*_3_)_3_). ^13^C-NMR (CDCl_3_): δ 169.22, 169.16, 168.8, 168.5, 168.1 (C=O), 151.2, 150.7, 150.7, 147.4, 141.0, 134.0, 132.7 (quat.), 143.5 (*C*H=CHCO), 130.8 (×2), 130.2, 128.1, 127.5, 126.2, 122.9, 121.9 (×2), 121.8, 111.0 (aromatic *C*H), 117.7 (CH=*C*HCO), 61.8 (*C*HN), 55.8 (O*C*H_3_), 51.8 (*C*(CH_3_)_3_), 44.4 (*C*H_2_Ar), 28.6 (*C*(CH_3_)_3_), 21.1 (×2), 20.6 (*C*H_3_CO). HRMS: *m*/*z* (ESI+): 631.2678 (M + H^+^). C_35_H_39_N_2_O_9_ requires 631.2656.

*(R,S)-(E)-N-(2-(tert-Butylamino)-1-(4-(hydroxy)phenyl)-2-oxoethyl)-3-(4-hydroxy-3-methoxyphenyl)-N-(2-(hydroxy)benzyl)acrylamide* (**8d**). It was prepared from triacetate **7c** (100 mg, 160 µmol) following the same procedure used for **8c**, Yield: 74.7 mg (93%). The purity by HPLC (for conditions see the general remarks) was 92%. R*_f_* 0.18 (PE/EtOAc 50:50) ^1^H-NMR (DMSO-*d*_6_, 90 °C): δ 9.33 (broad s, 1 H, O*H*), 9.08 (broad s, 2 H, O*H*), 7.40 (d, *J* = 15.3 Hz, 1 H, C*H*=CHCO), 7.13 (d, *J* = 8.5 Hz, 2 H), 7.03–6.58 (m, 9 H), 5.95 (s, 1 H, C*H*N), 4.75 (d, *J* = 17.0 Hz, 1 H, C*H*HAr), 4.50 (d, *J* = 17.0 Hz, 1 H, C*H*HAr), 3.77 (s, 3 H, OC*H*_3_), 1.26 (s, 9 H, C(C*H*_3_)_3_).

*(R,S)-(E)-3-(4-(Acetoxy)-3-methoxyphenyl)-N-(1-(4-(acetoxy)phenyl)-2-((4-(acetoxy)phenyl)amino)-2-oxoethyl)-N-benzylacrylamide* (**7e**). Triallyl derivative **6e** was prepared from 97 mg of aldehyde **3** (600 µmol), 105 mg of 4-(allyloxy)phenyl isocyanide [25] (660 µmol), 154.5 mg of acid **5** (660 µmol) and 72 µL of benzylamine (660 µmol) following the same procedure employed for **7c**. However, after 48 h, both aldehyde **3** and the starting isocyanide were visible at TLC. The reaction was worked out anyway. Chromatography (PE/EtOAc 60:40 + 1 % EtOH) gave pure **6e** (127 mg, 33%). Starting aldehyde (43 mg) was also recovered. Yield based on non-recovered starting aldehyde = 59%. This triallyl derivative **6e**, pure at TLC, was not fully characterized, but directly converted into **7e**, following the same procedure described for **7c**. Chromatography (PE/EtOAc 70:30 + 3% EtOH) gave pure **7e** (94 mg, 73%). White foam. R*_f_* 0.50 (PE/EtOAc 50:50). IR: υ_max_ 3282, 3070, 2988, 1756, 1697, 1621, 1595, 1546, 1505, 1494, 1453, 1409, 1367, 1310, 1187, 1163, 1106, 1075, 1046, 1014, 966, 909, 846, 757, 737, 700, 675, 634, 611 cm^−1^. ^1^H-NMR (CDCl_3_): δ 8.52 (s, 1 H, N*H*), 7.66 (d, *J* = 15.2 Hz, 1 H, C*H*=CHCO), 7.50 (d, *J* = 8.7 Hz 2 H), 7.45 (d, *J* = 8.4 Hz 2 H), 7.25–7.14 (m, 3 H), 7.12–6.89 (m, 8 H), 6.82 (s, 1 H), 6.68 (d, *J* = 15.3 Hz, 1 H, CH=C*H*CO), 6.30 (s, 1 H, C*H*N), 4.93 (d, *J* = 17.7 Hz, 1 H, C*H*HPh), 4.73 (d, *J* = 17.7 Hz, 1 H, C*H*HPh), 3.74 (s, 3 H, OC*H*_3_), 2.29, 2.27 (×2) (3 s, 3 x 3 H, C*H*_3_CO). ^13^C-NMR (CDCl_3_): δ 169.5, 169.1, 168.8, 168.4, 168.0 (C=O), 151.2, 150.8, 146.9, 141.0, 137.7, 135.5, 133.8, 131.8 (quat.), 143.7 (*C*H=CHCO), 130.8 (×2), 128.6 (×2), 127.3, 126.2 (×2), 123.1, 121.9 (×2), 121.8 (×2), 120.9 (×2), 120.8, 111.5 (aromatic *C*H), 118.1 (CH=*C*HCO), 63.1 (*C*HN), 55.8 (O*C*H_3_), 50.1 (*C*H_2_Ph), 21.1 (×2), 20.6 (*C*H_3_CO). HRMS: *m*/*z* (ESI+): 651.2371 (M + H^+^). C_37_H_35_N_2_O_9_ requires 631.2343.

*(R,S)-(E)-N-Benzyl-3-(4-hydroxy-3-methoxyphenyl)-N-(1-(4-hydroxyphenyl)-2-((4-hydroxyphenyl)-amino)-2-oxoethyl)acrylamide* (**8e**). It was prepared from triacetate **7e** (110 mg, 169 µmol) following the same procedure used for **8c**, Yield: 74.2 mg (84%). The purity by HPLC (for conditions see the general remarks) was 96%. R*_f_* 0.17 (PE/EtOAc 50:50) ^1^H-NMR (DMSO-*d*_6_, 90 °C): δ 9.67 (s, 1 H, O*H*), 9.14 (s, 1 H, O*H*), 8.99 (broad s, 1 H, N*H*), 8.84 (s, 1 H, O*H*), 7.42 (d, *J* = 15.3 Hz, 1 H, C*H*=CHCO), 7.32 (d, *J* = 9.0 Hz, 2 H), 7.22–7.05 (m, 7 H), 6.96 (broad s, 1 H), 6.90 (broad d, *J* = 8.1 Hz, 1 H), 6.78–6.65 (m, 6 H), 6.20 (s, 1 H, C*H*N), 4.89 (d, *J* = 17.1 Hz, 1 H, C*H*HAr), 4.63 (d, *J* = 17.1 Hz, 1 H, C*H*HAr), 3.75 (s, 3 H, OC*H*_3_).

*(R,S)-(E)-3-(4-(Acetoxy)-3-methoxyphenyl)-N-benzyl-N-(2-(tert-butylamino)-1-isopropyl-2-oxoethyl)acrylamide* (**7f**). Allyl derivative **6f** was prepared from 223.2 mg of acid **5** (1.05 mmol) and 1.1 eq. of benzylamine, isobutyraldehyde and *tert*-butyl isocyanide, following the same procedure employed for **7c**. However, after 48 h, acid **5** was still visible at TLC. The reaction was worked out anyway. Chromatography (PE/EtOAc 60:40 + 1% EtOH) gave pure **6f** (155 mg, 34%) This allyl derivative **6f**, pure at TLC, was not fully characterized, but directly converted into **7f**, following the same procedure described for **7c**. Chromatography (PE/EtOAc 70:30) gave pure **7f** (103.8 mg, 67%). White foam. R*_f_* 0.29 (PE/AcOEt 70:30). ^1^H-NMR (CDCl_3_): δ 7.63 (d, *J* = 15.3 Hz, 1 H, C*H*=CHCO), 7.35–7.18 (m, 5 H), 6.99–6.88 (m, 2 H), 6.79 (s, 1 H), 6.61 (d, *J* = 15.3 Hz, 1 H, CH=C*H*CO), 6.27 (broad s, 1 H, N*H*), 4.93 (d, *J* = 17.2 Hz, 1 H, C*H*HPh), 4.74 (d, *J* = 17.2 Hz, 1 H, C*H*HPh), 4.56 (broad d, *J* = 6.9 Hz, 1 H, C*H*N), 3.74 (s, 3 H, OC*H*_3_), 2.51–2.34 (m, 1 H, C*H*(CH_3_)_2_), 2.29 (s, 3 H, C*H*_3_CO), 1.29 (s, 9 H, C(C*H*_3_)_3_), 0.99 (d, *J* = 6.4 Hz, 3 H, C*H*_3_CH), 0.87 (d, *J* = 6.5 Hz, 3 H, C*H*_3_CH). ^13^C-NMR (CDCl_3_): δ 169.2, 168.8, 168.2 (C=O), 151.2, 140.9, 138.3, 134.1 (quat.), 142.5 (*C*H=CHCO), 128.6 (×2), 127.2, 126.4 (×2), 123.1, 120.8, 111.2 (aromatic *C*H), 118.8 (CH=*C*HCO), 65.6 (very broad) *C*HN), 55.8 (O*C*H_3_), 51.4 (*C*(CH_3_)_3_), 48.5 (very broad) (*C*H_2_Ph), 28.6 (*C*(CH_3_)_3_), 27.5 (*C*H(CH_3_)_2_, 20.6 (*C*H_3_CO), 19.7, 19.0 (*C*H_3_CH). HRMS: *m*/*z* (ESI+): 481.2708 (M + H^+^). C_28_H_37_N_2_O_5_ requires 481.2702.

*(R,S)-(E)-N-benzyl-N-(2-(tert-butylamino)-1-isopropyl-2-oxoethyl)-3-(4-hydroxy-3-methoxyphenyl)-acrylamide* (**8f**). Prepared from acetate **7f** (89.6 mg, 186 µmol) following the same procedure used for **8c**, Yield: 59.1 mg (73%). The purity by HPLC (for conditions see the general remarks) was 96%. R*_f_* 0.32 (PE/EtOAc 60:40). ^1^H-NMR (DMSO-*d*_6_, 90 °C; some signals were still rather broad at this temperature): δ 8.96 (broad s, 1 H, O*H*), 7.41 (d, *J* = 15.3 Hz, 1 H, C*H*=CHCO), 7.41 (broad s, 1 H, N*H*), 7.32–7.24 (m, 5 H), 7.23–7.11 (m, 1 H), 7.10–6.87 (broad m, 2 H), 6.77 (d, *J* = 8.1 Hz, 1 H), 4.94 (broad d, *J* = 16.2 Hz, 1 H, C*H*HPh), 4.75 (d, *J* = 16.2 Hz, 1 H, C*H*HPh), 3.79 (s, 3 H, OC*H*_3_), 2.37–2.23 (m, 1 H, C*H*(CH_3_)_2_), 1.22 (s, 9 H, C(C*H*_3_)_3_), 0.95 (d, *J* = 6.5 Hz, 3 H, C*H*_3_CH), 0.82 (d, *J* = 6.6 Hz, 3 H, C*H*_3_CH).

*(R,S)-(E)-N-(2-(4-(Acetoxy)benzyl)-1-isopropyl-2-oxoethyl)-3-(4-(Acetoxy)-3-methoxyphenyl)-N-benzylacrylamide* (**7g**). Diallyl derivative **6g** was prepared from 357 mg of acid **5** (1.52 mmol), 1 eq. each of benzylamine and isobutyraldehyde and 1.3 eq. of 4-(allyloxy)benzyl isocyanide [32], following the same procedure employed for **7c**. The reaction was worked out as usual after 48 h. As in our previous paper [32], the isocyanide was not stripped at high vacuum due to its volatility/lability and thus the actual amount used was slightly lower. Chromatography (PE/EtOAc 60:40) gave pure **6g** (382 mg, 44%) This diallyl derivative **6g**, pure at TLC, was not fully characterized, but directly converted into **7g**, following the same procedure described for **7c**. Chromatography (PE/EtOAc 70:30) gave pure **7g** (253 mg, 66%). White foam. R*_f_* 0.37 (PE/EtOAc 60:40). IR: υ_max_ 3290, 3065, 2965, 2874, 1759, 1673, 1647, 1600, 1507, 1453, 1418, 1367, 1301, 1257, 1213, 1190, 1156, 1121, 1080, 1031, 1013, 972, 941, 908, 830, 731, 697, 645 cm^−1^. ^1^H-NMR (CDCl_3_): δ 7.62 (d, *J* = 15.3 Hz, 1 H, C*H*=CHCO), 7.31–7.16 (m, 8 H), 7.02 (d, *J* = 8.7 Hz, 2 H), 6.99–6.90 (m, 2 H), 6.81 (d, *J* = 1.2 Hz, 1 H), 6.63 (d, *J* = 15.3 Hz, 1 H, CH=C*H*CO), 4.83, 4.77 (AB syst., *J* = 17.4 Hz, 2 H, C*H*_2_N), 4.62 (broad d, *J* = 10.5 Hz, 1 H, C*H*N), 4.43 (dd, *J* = 14.9, 6.1 Hz, 1 H C*H*HNH), 4.27 (dd, *J* = 14.9, 5.7 Hz, 1 H C*H*HNH), 3.76 (s, 3 H, OC*H*_3_), 2.59–2.43 (m, 1 H, C*H*(CH_3_)_2_), 2.30 (s, 3 H, C*H*_3_CO), 2.28 (s, 3 H, C*H*_3_CO), 1.00 (d, *J* = 6.4 Hz, 3 H, C*H*_3_CH), 0.87 (d, *J* = 6.6 Hz, 3 H, C*H*_3_CH). ^13^C-NMR (CDCl_3_): δ 170.1, 169.4, 168.8, 168.5 (C=O), 151.2, 149.8, 141.0, 137.7, 135.8, 133.9 (quat.), 143.0 (*C*H=CHCO), 128.8 (×2), 128.7 (×2), 127.4, 126.4 (×2), 123.1, 121.7 (×2), 120.8, 111.3 (aromatic *C*H), 118.4 (CH=*C*HCO), 65.8 (very broad) (*C*HN), 55.8 (O*C*H_3_), 49.2 (very broad) (*C*H_2_Ph), 42.7 (*C*H_2_NH), 27.1 (*C*H(CH_3_)_2_, 21.1 (*C*H_3_CO), 20.6 (*C*H_3_CO), 19.9, 19.2 (*C*H_3_CH). HRMS: *m*/*z* (ESI+): 573.2608 (M + H^+^). C_33_H_37_N_2_O_7_ requires 573.2601.

*(R,S)-(E)-N-benzyl-N-(2-(4-hydroxybenzyl)-1-isopropyl-2-oxoethyl)-3-(4-hydroxy-3-methoxyphenyl)-acrylamide* (**8g**). Prepared from acetate **7g** (104.2 mg, 182 µmol) following the same procedure used for **8c**, Yield: 71.9 mg (73%). The purity by HPLC (for conditions see the general remarks) was 96%. R*_f_* 0.35 (PE/EtOAc 50:50). ^1^H-NMR (DMSO-*d*_6_, 90 °C; some signals were still rather broad at this temperature): δ 9.00 (broad s, 1 H, O*H*), 8.93 (s, 1 H, N*H*), 8.27 (s, 1 H, O*H*), 7.40 (d, *J* = 15.2 Hz, 1 H, C*H*=CHCO), 7.36–7.14 (m, 7 H), 7.05 (d, *J* = 8.4 Hz, 2 H), 7.05–6.93 (broad m, 2 H), 6.77 (d, *J* = 7.8 Hz, 1 H), 6.70 (d, *J* = 8.4 Hz, 2 H), 4.95–4.55 (m, 3 H, C*H*_2_Ph and C*H*N), 4.12 (d, *J* = 6.0 Hz, 2 H, C*H*_2_NH), 3.78 (s, 3 H, OC*H*_3_), 2.41–2.22 (m, 1 H, C*H*(CH_3_)_2_), 0.92 (d, *J* = 6.6 Hz, 3 H, C*H*_3_CH), 0.81 (d, *J* = 6.6 Hz, 3 H, C*H*_3_CH).

*N-3-(Allyloxy)benzyl formamide*. Amine **2**, prepared as previously described [25] (1.10 g, 6.74 mmol) was dissolved in ethyl formate (27 mL) and heated at reflux for 16 h. Evaporation of the solvent gave the title compound in quantitative yield (1.289 g) as a white solid. M.p.: 39.3–41.5 °C. R*_f_* 0.12 (PE/AcOEt 50:50). IR: υ_max_ 3271, 3044, 2933, 2914, 2888, 2863, 2775, 1642, 1614, 1587, 1539, 1486, 1452, 1423, 1388, 1367, 1347, 1310, 1295, 1261, 1236, 1216, 1156, 1098, 1035, 992, 946, 913, 884, 813, 772, 730, 693, 658, 625 cm^−1^. ^1^H NMR (CDCl_3_) (two conformers A and B in 87:13 ratio, are visible): δ: δ 8.24 (A) (s, 0.87 H, C*H*=O), 8.15 (B) (d, *J* = 11.9 Hz, 0.13 H, C*H*=O), 7.30–7.19 (A + B) (m, 1 H), 6.90–6.77 (A + B) (m, 3 H), 6.04 (A + B) (ddt, *J* = 17.1, 10.5 (d), 5.3 Hz (t), 1 H, C*H*=CH_2_), 5.99 (A + B) (broad s, N*H*), 5.28 (A + B) (dq, *J* = 10.5 (d), 1.2 Hz (q), 1 H CH=C*H*H), 4.56–4.49 (A + B) (m, 2 H, C*H*_2_CH=CH_2_), 4.44 (A) (d, *J* = 5.9 Hz, 1.74 H, C*H*_2_Ar), 4.36 (B) (d, *J* = 6.5 Hz, 0.26 H, C*H*_2_Ar). ^13^C-NMR (CDCl_3_) only the peaks of major conformer are listed): δ 161.0, 158.9 (*C*=O), 139.1, 129.8 (quat.), 133.0 (*C*=CH_2_), 129.8, 120.1, 114.2, 113.8 (aromatic *C*H), 117.7 (CH=*C*H_2_), 68.7 (*C*H_2_CH=CH_2_), 42.0 (Ar*C*H_2_). HRMS: *m*/*z* (ESI+): 192.1017 (M + H^+^). C_11_H_14_NO_2_ requires 192.1025.

*3-(Allyloxy)benzyl isocyanide*. *N*-3-(Allyloxy)benzyl formamide (386 mg, 2.02 mmol) was dissolved in dry CH_2_Cl_2_ (20 mL), cooled to −30 °C and treated with triethylamine (1.30 mL, 9.29 mmol) and POCl_3_(282 µL, 3.05 mmol). After stirring for 3 h at the same temperature, the brown solution was poured into saturated aqueous NaHCO_3_, and extracted three times with CH_2_Cl_2_. The organic extracts were washed with brine, evaporated to dryness and chromatographed (PE/EtOAc 95:5) to give the pure title compound as a colorless liquid. Due to its partial volatility, it was evaporated only at 15 mbar and 25 °C. The yield (331 mg, 95%) is therefore slightly overestimated, since it probably contains few amounts of solvent. Ths isocyanide was directly used for the next Ugi reaction to give **6h** (see below).

*(R,S)-(E)-N-(2-(3-(Acetoxy)benzyl)-1-(4-acetoxyphenyl)-2-oxoethyl)-3-(4-(Acetoxy)-3-methoxyphenyl)-N-benzylacrylamide* (**7h**). Triallyl derivative **6h** was prepared from 243 mg of aldehyde **3** (1.50 mmol), 387 mg of acid **5** (1.65 mmol), 1.1 eq. of benzylamine and and freshly prepared 3-(allyloxy)benzyl isocyanide (330 mg, 1.91 mmol), following the same procedure employed for **7c**. The reaction was worked out as usual after 48 h. Chromatography (PE/EtOAc 60:40) gave pure **6h** (580 mg, 59%). Chromatography gave also mg 90 of recovered starting aldehyde **3**. Thus, the yield from unrecovered starting aldehyde is 93%. This triallyl derivative **6h**, pure at TLC, was not fully characterized, but directly converted into **7h**, following the same procedure described for **7c**. Chromatography (PE/EtOAc 40:60) gave pure **7h** (560 mg, 96%). White foam. R*_f_* 0.51 (PE/EtOAc 50:50). IR: υ_max_ 3297, 3064, 2938, 1759, 1677, 1648, 1601, 1506, 1451, 1418, 1367, 1299, 1257, 1189, 1155, 1121, 1081, 1014, 976, 956, 907, 829, 793, 731, 696, 635 cm^−1^. ^1^H-NMR (CDCl_3_): δ 7.70 (d, *J* = 15.3 Hz, 1 H, C*H*=CHCO), 7.42 (d, *J* = 8.4 Hz, 2 H), 7.31 (t, *J* = 7.7 Hz, 1 H), 7.25–6.94 (m, 12 H), 6.84 (s, 1 H), 6.64 (d, *J* = 15.3 Hz, 1 H, CH=C*H*CO), 6.33 (broad t, *J* = 5.7 Hz, 1 H, N*H*), 6.08 (s, 1 H, C*H*N), 4.89, 4.65 (AB syst., *J* = 17.8 Hz, 2 H, C*H*_2_Ph), 4.51, 4.46 (AB part of an ABX syst, J_AB_ = 14.6, J_AX_ = 5.0, J_BX_ = 6.0 Hz, C*H*_2_NH), 3.76 (s, 3 H, OC*H*_3_), 2.29 (s, 3 H, C*H*_3_CO), 2.27 (s, 3 H, C*H*_3_CO), 2.26 (s, 3 H, C*H*_3_CO). ^13^C-NMR (CDCl_3_): δ 169.5, 169.4, 169.1, 168.8, 168.0 (C=O), 151.1, 150.8, 141.0, 139.7, 137.8, 134.0, 132.2 (quat.), 143.2 (*C*H=CHCO), 131.0 (×2), 129.6, 128.6 (×2), 127.1, 126.2 (×2), 125.1, 123.0, 121.9 (×2), 120.84, 120.81, 120.6, 111.3 (aromatic *C*H), 118.3 (CH=*C*HCO), 62.3 (*C*HN), 55.8 (O*C*H_3_), 50.0 (*C*H_2_Ph), 43.2 (*C*H_2_NH), 21.1 (*C*H_3_CO), 21.0 (*C*H_3_CO), 20.6 (*C*H_3_CO). HRMS: *m*/*z* (ESI+): 665.2522 (M + H^+^). C_38_H_37_N_2_O_9_ requires 665.2499.

*(R,S)-(E)-N-Benzyl-N-(2-(3-(hydroxy)benzyl)-1-(4-hydroxyphenyl)-2-oxoethyl)-3-(4-hydroxy-3-methoxyphenyl)acrylamide* (**8h**). Prepared from acetate **7h** (100.0 mg, 150 µmol) following the same procedure used for **8c**. Yield: 76.2 mg (94%). The purity by HPLC (for conditions see the general remarks) was 94%. R*_f_* 0.32 (PE/EtOAc 60:40). ^1^H-NMR (DMSO-*d*_6_, 90 °C) (some signals were still rather broad at this temperature): δ 9.09 (s, 1 H), 8.90 (s, 2 H), 8.27 (broad s, 1 H), 7.41 (d, *J* = 15.3 Hz, 1 H, C*H*=CHCO), 7.25–7.02 (m, 9 H), 6.96 (s, 1 H), 6.90 (d, *J* = 8.1 Hz, 1 H), 6.75 (d, *J* = 8.1 Hz, 1 H), 6.72–6.61 (m, 5 H), 6.14 (broad s, C*H*N), 4.88 (d, *J* = 17.1 Hz, 1 H, C*H*HPh), 4.61 (d, *J* = 17.1 Hz, 1 H, C*H*HPh), 4.26, 4.21 (AB part of an ABX syst, J_AB_ = 15.2, J_AX_ = 5.9, J_BX_ = 6.2 Hz, C*H*_2_NH), 3.76 (s, 3 H, OC*H*_3_).

*N-Benzyl-N-(2-(tert-butylamino)-2-oxo-1-phenylethyl)cinnamamide* (**9**). Benzaldehyde (153 µL, 1.50 mmol), benzylamine (180 µL, 1.65 mmol), cinnamic acid (245 mg, 1.65 mmol) and *tert*-butyl isocyanide (185 µL, 1.65 mmol) were reacted as described for the synthesis of **6c**. Chromatography (PE: AcOEt 75:25) gave pure **9** as a white foam (571 mg, 89%). R*_f_* 0.36 (PE/EtOAc 75:25). IR: υ_max_ 3316, 3063, 3030, 2971, 2926, 1650, 1596, 1547, 1496, 1470, 1450, 1411, 1392, 1363, 1351, 1331, 1303, 1284, 1253, 1220, 1201, 1189, 1174, 1078, 1032, 997, 976, 947, 915, 892, 860, 841, 804, 768, 758, 740, 723, 695, 642, 622, 615 cm^−1^. ^1^H-NMR (CDCl_3_): δ 7.77 (d, *J* = 15.3 Hz, 1 H, C*H*=CHCO), 7.43–7.08 (m, 14 H), 7.01 (broad d, *J* = 6.9 Hz, 1 H), 6.72 (d, *J* = 15.3 Hz, 1 H, PhC*H*=CH), 6.11 (s, 1 H, C*H*N), 5.68 (s, 1 H, N*H*), 4.93, 4.69 (AB syst., *J* = 17.9 Hz, 2 H, C*H*_2_Ph), 1.35 (s, 9 H, C(C*H*_3_)_3_). ^13^C-NMR (CDCl_3_): δ 168.9, 168.2 (C=O), 138.2, 135.3, 135.1 (quat.), 143.7 (*C*H=CHCO), 129.7 (×3), 128.7 (×2), 128.6 (×2), 128.3 (×3), 127.9, 126.9 (×2), 126.2 (×2) (aromatic *C*H), 118.2 (CH=*C*HCO), 62.9 (*C*HN), 51.7 (*C*(CH_3_)_3_), 49.7 (*C*H_2_Ph), 28.6 (C(*C*H_3_)_3_). HRMS: *m*/*z* (ESI+): 427.2378 (M + H^+^). C_28_H_31_N_2_O_2_ requires 427.2386.

*(E)-N-(1-(4-Acetoxyphenyl)-2-(tert-butylamino)-2-oxoethyl))-3-(4-acetoxy-3-methoxyphenyl)-N-((S)-1-phenylethyl)acrylamide* (**12a**) *and (E)-N-(1-(4-Acetoxyphenyl)-2-(tert-butylamino)-2-oxoethyl))-3-(4-acetoxy-3-methoxyphenyl)-N-((S)-1-phenylethyl)acrylamide* (**12b**). Aldehyde **3** (243 mg, 1.50 mmol), protected ferulic acid **5** (387 mg, 1.65 mmol), (*S*)-α-methylbenzylamine (212.7 µL, 1.65 mmol) and *tert*-butyl isocyanide (185 µL, 1.65 mmol) were reacted as described for the synthesis of **6c**. Chromatography (PE/EtOAc 70:30) gave: the faster running diastereomer **11a** (182.1 mg) (R_*f*_ = 0.27, PE/EtOAc 70:30), the slower running diastereomer **11b** (190.4 mg) (R_*f*_ = 0.19, PE/EtOAct 70:30), and some mixed fractions (37.6 mg). A second chromatography of the mixed fractions gave additional **11a** (21.7 mg) and **11b** (15.9 mg). Overall yield: 420.1 mg (44%). Diastereomeric ratio = 50:50. The realtive configuration was not established. Diallyl derivatives **11a** and **11b**, pure at TLC, were not fully characterized, but directly independently converted into **12a** and **12b**, following the same procedure described for **7c**.

**12a**. Obtained in 45% yield (96.3 mg from 212.1 mg of **11a**) after chromatography with PE/EtOAc 60:40). R*_f_* 0.34 (PE/EtOAc 60:40). IR: υ_max_ 3300, 2965, 2930, 1768, 1689, 1644, 1603, 1547, 1508, 1452, 1434, 1416, 1389, 1368, 1337, 1260, 1193, 1152, 1123, 1034, 1014, 978, 946, 909, 883, 846, 828, 794 cm^−1^. ^1^H-NMR (CDCl_3_) (due to the presence of conformers around the tertiary amide, the signals are rather broad and splitting of some signal is present): δ 7.72 (d, *J* = 15.0 Hz, 1 H, C*H*=CHCO), 7.64–7.25 (m, 7 H), 7.10 (d, *J* = 8.7 Hz, 2 H), 6.97–6.78 (m, 2 H), 6.73–6.53 (m, 1 H), 6.44 (d, *J* = 15.0 Hz, 1 H, CH=C*H*CO), 6.59 (minor conformer) and 5.43 (major conformer) (2 very broad s, 1 H, C*H*CH_3_), 5.00 (s, 1 H, C*H*N), 4.78 (s, 1 H, N*H*), 3.72 (s, 3 H, OC*H*_3_), 2.29 (s, 6 H, C*H*_3_CO), 1.53–1.17 (m, 6 H, C*H*_3_CH and C(C*H*_3_)_3_ (minor conformer)), 0.94 (s, 6 H, C(C*H*_3_)_3_, major conformer). ^13^C-NMR (CDCl_3_): δ 169.2, 168.8, 168.3, 167.6 (C=O), 151.1, 150.0, 142.7, 141.0, 140.9, 134.0, 133.3 (quat.), 142.7 (*C*H=CHCO), 129.7 (×2), 129.0 (×2), 128.6, 127.9 (×2), 123.0, 121.7, 121.1 (×2), 111.3 (aromatic *C*H), 119.9 (CH=*C*HCO), 63.0 (*C*(CH_3_)_3_), 59.7 (*C*HN), 55.7 (O*C*H_3_), 51.5 (*C*HPh), 28.3 (C(*C*H_3_)_3_), 21.1, 20.6 (*C*H_3_CO), 16.3 (CH*C*H_3_). HRMS: *m*/*z* (ESI+): 587.2774 (M + H^+^). C_34_H_39_N_2_O_7_ requires 587.2757.

**12b**. Obtained in 67% yield (145.5 mg from 216.5 mg of **11a**) after chromatography with PE/EtOAc 60:40). R*_f_* 0.30 (PE/EtOAc 60:40). IR: υ_max_ 3300, 2970, 2936, 1761, 1685, 1646, 1600, 1545, 1506, 1452, 1431, 1417, 1393, 1366, 1339, 1260, 1191, 1154, 1121, 1031, 1012, 975, 945, 908, 882, 844, 827, 792 cm^−1^. ^1^H-NMR (CDCl_3_) (due to the presence of conformers around the tertiary amide, the signals are rather broad and splitting of some signal is present): δ 7.64 (d, *J* = 15.0 Hz, 1 H, C*H*=CHCO), 7.50–7.12 (m, 6 H), 7.10–6.85 (m, 4 H), 6.82 (d, *J* = 15.0 Hz, 1 H, CH=C*H*CO), 6.52 (s, 1 H, N*H*), 5.38 (C*H*CH_3_), 5.05 (C*H*N), 3.80 (s, 3 H, OC*H*_3_), 2.31 (s, 3 H, C*H*_3_CO), 2.25 (s, 3 H, C*H*_3_CO), 1.92–1.60 (m, 3 H, C*H*_3_CH), 1.35 (s, 9 H, C(C*H*_3_)_3_). ^13^C-NMR (CDCl_3_): δ 169.5, 169.1, 168.8, 167.3 (C=O), 151.2, 150.0, 140.9, 139.9, 134.2, 133.9 (quat.), 142.6 (*C*H=CHCO), 129.7 (×2), 128.5 (×2), 127.8, 127.3 (×2), 123.1, 121.4 (×2), 120.7 (×2), 111.2 (aromatic *C*H), 119.3 (CH=*C*HCO), 64.2 (*C*HN), 60.4 (*C*(CH_3_)_3_), 55.8 (O*C*H_3_ and *C*HPh), 28.3 (C(*C*H_3_)_3_), 21.1, 20.6 (*C*H_3_CO), 16.3 (CH*C*H_3_). HRMS: *m*/*z* (ESI+): 587.2768 (M + H^+^). C_34_H_39_N_2_O_7_ requires 587.2757.

*(E)-N-(2-(tert-Butylamino)-1-(4-hydroxyphenyl)-2-oxoethyl))-3-(4-hydroxy-3-methoxyphenyl)-N-((S)-1-phenylethyl)acrylamide* (**13a**) and *(E)-N-(2-(tert-Butylamino)-1-(4-hydroxyphenyl)-2-oxoethyl))-3-(4-hydroxy-3-methoxyphenyl)-N-((S)-1-phenylethyl)acrylamide* (**13b**). They were independently prepared respectively from diacetates **12a** and **12b**, following the same procedure used for **8c**.

**13a**. Yield: 90%. The purity by HPLC (for conditions see the general remarks) was 94%. R*_f_* 0.29 (PE/EtOAc 50:50). ^1^H-NMR (DMSO-*d*_6_, 90 °C): δ 9.09 (broad s, 2 H, O*H*), 7.50 (d, *J* = 7.5 Hz, 2 H), 7.36–7.17 (m, 6 H, aromatic C*H* and C*H*=CHCO), 6.94 (s, 1 H, N*H*), 6.81–6.69 (m, 5 H, aromatic C*H*), 6.50 (d, *J* = 15.3 Hz, CH=C*H*CO), 5.79 (broad m, 1 H, C*H*CH_3_), 5.45 (s, 1 H, C*H*N), 3.74 (s, 3 H, OC*H*_3_), 1.49 (d, *J* = 7.0 Hz, 3 H, C*H*_3_CH), 1.14 (s, 9 H, C(C*H*_3_)_3_).

**13b**. Yield: 85%. The purity by HPLC (for conditions see the general remarks) was 98%. R*_f_* 0.34 (PE/EtOAc 50:50). ^1^H-NMR (DMSO-*d*_6_, 90 °C): δ 9.02 (broad s, 2 H, O*H*), 7.65 (s, 1 H, N*H*), 7.41–7.16 (m, 7 H, aromatic C*H* and C*H*=CHCO), 6.86 (d, *J* = 7.9 Hz, 2 H), 6.81–6.67 (m, 3 H, aromatic C*H*), 6.60 (d, *J* = 8.6 Hz, 1 H), 6.51 (d, *J* = 15.3 Hz, CH=C*H*CO), 5.68 (broad m, 1 H, C*H*CH_3_), 5.35 (s, 1 H, C*H*N), 3.75 (s, 3 H, OC*H*_3_), 1.66 (d, *J* = 7.0 Hz, 3 H, C*H*_3_CH), 1.35 (s, 9 H, C(C*H*_3_)_3_).

*(R,S)-(E)-3-(4-(Allyloxy)-3-methoxyphenyl)-N-benzyl-N-(1-(tert-butyl)-2-oxoazetidin-3-yl)acrylamide* (**16a**). A solution of glycolaldehyde dimer (135 mg, 1.125 mmol) in dry ethanol (7.5 mL), was treated with benzylamine (246 µL, 2.25 mmol) and freshly activated powdered 3 Å molecular sieves (115 mg). The suspension was stirred for 6 h at rt. Then, allylated ferulic acid **5** (350 mg, 1.50 mmol) and *tert*-butyl isocyanide (220 µL, 1.94 mmol) were added. After stirring for 48 h at rt, the mixture was diluted with CH_2_Cl_2_/MeOH 1:1 and filtered through a Celite cake. After evaporation of the solvent, the crude was taken up in EtOAc and washed with saturated aqueous NaHCO_3_ to remove excess of **5**, and then with brine. Evaporation of the organic phase to dryness, followed by chromatography (PE/EtOAc 50:50 + 2% EtOH) gave pure **15a** as a brownish foam (292 mg, 42%). This compound (277 mg, 0.59 mmol) was taken up in dry DMF (2.0 mL) cooled at 0 °C and treated with sulfonyl diimidazole (177 mg, 0.89 mmol) and NaH (60% in mineral oil) (36 mg, 0.89 mmol). After stirring for 2.5 h at rt, the mixture was diluted with saturated aqueous NH_4_Cl (30 mL) and Et_2_O/DCM 20:1. The phases were separated, and the aqueous one re-extracted three times with Et_2_O/DCM 20:1. The organic phases were washed with brine (×4), evaporated to dryness and chromatographed (PE/EtOAc 3:2 + 2% EtOH) to give pure **16a** as a yellowish oil (115 mg, 43%). R*_f_* 0.49 (PE/EtOAc 50:50 + 2% EtOH). ^1^H- NMR (CDCl_3_): δ 7.72 (d, *J* = 15.3 Hz, 1 H, C*H*=CHCO), 7.41–7.24 (m, 5 H), 7.01 (d, *J* = 7.8 Hz, 1 H), 6.91 (s, 1 H), 6.82 (d, *J* = 8.4 Hz, 1 H), 6.66 (d, *J* = 15.3 Hz, 1 H, CH=C*H*CO), 6.06 (ddt, *J* = 5.4 (t), 10.6, 17.6 Hz (d), 1 H, C*H*=CH_2_), 5.40 (dq, *J* = 17.6 (d), 0.9 Hz (q), 1 H, C*H*H=CH), 5.30 (dq, *J* = 10.6 (d), 0.9 Hz (q), 1 H, C*H*H=CH), 4.97 (broad s, 1 H, C*H*N), 4.86, 4.78 (AB syst., *J* = 17.4 Hz, 2 H, C*H*_2_Ph), 4.62 (d, *J* = 5.4 Hz, 2 H, C*H*_2_CH=CH_2_), 3.85 (s, 3 H, OC*H*_3_), 3.39 (t, *J* = 5.1 Hz., C*H*HCO), 3.18 (broad s, 1 H, C*H*HCO), 1.24 (s, 9 H, C(C*H*_3_)_3_). ^13^C-NMR (CDCl_3_): δ 167.7, 164.8 (*C*=O), 149.8, 149.4, 137.6, 128.2 (quat.), 144.2 (*C*H=CHCO), 132.8 (*C*H=CH_2_), 128.9 (×2), 127.8, 126.7 (×2), 121.9, 112.9, 110.4 (aromatic *C*H), 118.3 (CH=*C*H_2_), 114.7 (CH=*C*HCO), 69.7 (*C*H_2_CH=CH_2_), 61.3 (*C*HN), 56.0 (O*C*H_3_), 53.2 (*C*(CH_3_)_3_), 51.0 (*C*H_2_Ph), 43.7 ((*C*H_2_CO), 27.4 (C(*C*H_3_)_3_). HRMS: *m*/*z* (ESI+): 449.2443 (M + H^+^). C_27_H_33_N_2_O_4_ requires 449.2440.

*(R,S)-(E)-3-(4-(Allyloxy)-3-methoxyphenyl)-N-(1-(4-allyloxyphenyl)-2-oxoazetidin-3-yl)-N-benzyl-acrylamide* (**16b**). Prepared following the same procedure above described for **16a**. Starting from 350 mg of allylated ferulic acid **5** (1.50 mmol), pure **15b** was obtained after chromatography (PE/EtOAc from 50:50 + 1% AcOH to 50:50 + 2% EtOH) as yellow-green oil (222 mg, 28%). Then, this alcohol (210 mg, 0.40 mmol) was converted into **16b** as described for **16a**. Chromatography: PE/EtOAc from 75:25 + 2% EtOH to 50:50 + 4% EtOH. Yield: 139 mg, 66%. R*_f_* 0.35 (PE/EtOAc 70:30 + 2% EtOH). ^1^H-NMR (CDCl_3_): δ 7.73 (d, *J* = 15.2 Hz, 1 H, C*H*=CHCO), 7.40–7.22 (m, 7 H), 7.00 (d, *J* = 8.3 Hz, 1 H), 6.92–6.78 (m, 4 H), 6.65 (d, *J* = 15.2 Hz, 1 H, CH=C*H*CO), 6.04 (ddt, *J* = 5.4 (t), 10.6, 17.6 Hz (d), 1 H, C*H*=CH_2_), 6.03 (ddt, *J* = 5.4 (t), 10.6, 17.6 Hz (d), 1 H, C*H*=CH_2_), 5.39 (dq, *J* = 17.4 (d), 1.5 Hz (q), 2 H, C*H*H=CH), 5.30 (dq, *J* = 10.6 (d), 0.9 Hz (q), 1 H, C*H*H=CH), 5.28 (dq, *J* = 10.6 (d), 0.9 Hz (q), 1 H, C*H*H=CH), 5.25 (broad s, 1 H, C*H*N), 4.83 (broad s, 2 H, C*H*_2_Ph), 4.62 (dt, *J* = 5.4 (d), 1.4 Hz (t), 2 H, C*H*_2_CH=CH_2_), 4.50 (dt, *J* = 5.3 (d), 1.5 Hz (t), 2 H, C*H*_2_CH=CH_2_), 3.83 (s, 3 H, OC*H*_3_), 3.79 (t, *J* = 5.7 Hz., C*H*HCO), 3.63 (broad s, 1 H, C*H*HCO). ^13^C-NMR (CDCl_3_): δ 167.7, 162.9 (*C*=O), 155.2, 149.9, 149.4, 137.1, 131.8, 128.0 (quat.), 144.6 (*C*H=CHCO), 133.1, 132.7 (*C*H=CH_2_), 129.0 (×2), 127.9, 126.6 (×2), 122.1, 117.9 (×2), 115.3 (×2), 112.9, 110.3 (aromatic *C*H), 118.3, 117.7 (CH=*C*H_2_), 114.3 (CH=*C*HCO), 69.7, 69.1 (*C*H_2_CH=CH_2_), 62.6 (*C*HN), 55.9 (O*C*H_3_), 51.4 (*C*H_2_Ph), 45.7 ((*C*H_2_CO). HRMS: *m*/*z* (ESI+): 525.2398 (M + H^+^). C_32_H_33_N_2_O_5_ requires 525.2389.

*(R,S)-(E)-N-Benzyl-N-(1-(tert-butyl)-2-oxoazetidin-3-yl)-3-(4-(hydroxy)-3-methoxyphenyl)-acrylamide* (**17a**). A solution of allyl derivative **16a** (84 mg, 187 µmol), Pd(PPh_3_)_2_Cl_2_ (2.0 mg, 3.68 µmol, 0.015 eq. calculated on the number of allyl groups), and ammonium formate (18 mg, 280 µmol, 1.5 eq. calculated on the number of allyl groups) in dry CH_3_CN (2.0 mL) in a pressure tube was first flushed with argon and then sealed and heated at 81 °C for 24 h. A tlc showed that the reaction was complete. The resulting dark solution was diluted with EtOAc and saturated aqueous NaHCO_3_. The phases were separated and the aqueous one re-extracted three times with EtOAc. The organic phases were washed with brine and evaporated to dryness. Chromatography (PE/EtOAc 2:3 + 2% EtOH) gave pure **17a** as a white foam (71 mg, 93%). The purity by HPLC (for conditions see the general remarks) was >99%. R*_f_* 0.30 (PE/EtOAc 50:50 + 2% EtOH). ^1^H-NMR (CDCl_3_): δ 7.70 (d, *J* = 15.3 Hz, 1 H, C*H*=CHCO), 7.41–7.25 (m, 5 H), 7.01 (d, *J* = 7.5 Hz, 1 H), 6.89 (s, 1 H), 6.88 (d, J not measurable, 1 H), 6.63 (d, *J* = 15.3 Hz, 1 H, CH=C*H*CO), 5.97 (s, 1 H, O*H*), 4.96 (broad s, 1 H, C*H*N), 4.84, 4.77 (AB syst., *J* = 17.3 Hz, 2 H, C*H*_2_Ph), 3.87 (s, 3 H, OC*H*_3_), 3.38 (t, *J* = 5.3 Hz., C*H*HCO), 3.18 (broad s, 1 H, C*H*HCO), 1.24 (s, 9 H, C(C*H*_3_)_3_). ^13^C-NMR (CDCl_3_): δ 167.8, 164.9 (*C*=O), 147.7, 146.7, 137.6, 127.5 (quat.), 144.3 (*C*H=CHCO), 128.9 (×2), 127.8, 126.7 (×2), 122.3, 114.8, 109.9 (aromatic *C*H), 114.2 (CH=*C*HCO), 61.3 (*C*HN), 56.0 (O*C*H_3_), 53.2 (*C*(CH_3_)_3_), 51.0 (*C*H_2_Ph), 43.7 ((*C*H_2_CO), 27.4 (C(*C*H_3_)_3_). HRMS: *m*/*z* (ESI+): 409.2133 (M + H^+^). C_24_H_29_N_2_O_4_ requires 409.2127.

*(R,S)-(E)-N-Benzyl-3-(4-(hydroxy)-3-methoxyphenyl)-N-(1-(4-hydroxyphenyl)-2-oxoazetidin-3-yl)acrylamide* (**17b**). It was prepared from **16b** (93 mg, 177 µmol), following the same procedure described above for **17a**, but in this case the workup was only the evaporation to dryness due to the low solubility of the product. Chromatography (DCM/MeOH 100:4) gave pure **17b** as a white solid (71 mg, 90%). The purity by HPLC (for conditions see the general remarks) was 99%. R*_f_* 0.42 (PE/ EtOAc 40:60 + 2% EtOH). ^1^H-NMR (DMSO-*d*_6_, 90 °C): δ 9.06 (s, 1 H, O*H*), 8.98 (s, 1 H, O*H*), (7.48 (d, *J* = 15.2 Hz, 1 H, C*H*=CHCO), 7.39–7.22 (m, 5 H), 7.21–7.14 (m, 3 H), 7.03 (dd, *J* = 8.2, 1.9 Hz, 1 H), 6.88 (d, *J* = 15.2 Hz, 1 H, CH=C*H*CO), 6.81–6.73 (m, 3 H), 5.26 (broad s, 1 H, C*H*N), 4.92, 4.77 (AB syst., *J* = 16.4 Hz, 2 H, C*H*_2_Ph), 3.83 (t, *J* = 5.6 Hz, C*H*HCO), 3.79 (s, 3 H, OC*H*_3_), 3.64–3.57 (m, 1 H, C*H*HCO). ^13^C-NMR (DMSO*-d*_6_, 90 °C): δ 166.7, 163.2 (*C*=O), 153.4, 148.8, 147.7, 138.5, 130.6, 126.2 (quat.), 143.3 (*C*H=CHCO), 128.6 (×2), 127.2, 126.6 (×2), 122.6, 117.4 (×2), 115.4 (×3), 111.2 (aromatic *C*H), 114.2 (CH=*C*HCO), 63.2 (*C*HN), 55.6 (O*C*H_3_), 51.5 (*C*H_2_Ph), 44.8 ((*C*H_2_CO). HRMS: *m*/*z* (ESI+): 445.1766 (M + H^+^). C_26_H_25_N_2_O_5_ requires 445.1763.

### 3.3. Thioflavin Experiments

One milliliter of DMSO was added to 1 mg of lyophilized synthetic peptide (Aβ1-42, AβpE3-42, AnaSpec, Fremont, CA, USA), to reach a final concentration of 1 mg mL^−1^. Aliquots of 75 μL were lyophilized and stored at −20 °C until being used. For all experiments, stock peptides were reconstituted as reported [37]. For the preparation of the working samples, a stock solution of each peptide was divided into two or more aliquots. One was diluted to 5 μM in PBS containing 1% (*v*/*v*) DMSO to have a reference sample, and the others were diluted in PBS containing the appropriate quantity of polyphenol stock solution in DMSO in such a manner that each sample contains 1% of DMSO. The final pH was measured and eventually corrected to 7.4 using a few μL of 1 M HCl. Aβ peptides (5 μM) were incubated at 37 °C in the presence/absence of polyphenols as previously described and analyzed in parallel. ThT fluorescence was followed in time during aggregation. For this purpose, 47.5 μL of Aβ with and without test compounds were mixed with 2.5 μL ThT (400 μM) in a 3 mm path length fluorescence cuvette. ThT fluorescence was measured by using a luminescence spectrometer (LS50B, PerkinElmer, Waltham, MA, USA) at excitation and emission wavelengths of 440 nm (slit width = 5 nm) and 482 nm (slit width = 10 nm), respectively. ThT fluorescence data were plotted as a function of time and fitted by a sigmoidal curve described by the following equation: [38] $y=y_{i}+\frac{y_{f-}y_{i}}{1+e^{\left(t-t_{0}\right)k_{fib}}}$ where *y_i_* and *y_f_* are the initial and final ThT fluorescence, respectively and *k_fib_* is the fibril growing rate, *t* is time and *t_0_* is the time to 50% of maximal fluorescence. The lag time (T) is derived as *t_0_* – 2/*k_fib_*.

## 4. Conclusions

In conclusion, the present study has demonstrated that replacement of the various pharmacophores in our previous leads may be critical, only small changes being permitted. A new possible hit has been selected from the second-generation library produced during this work. From the synthetic point of view, the feasibility of the overall strategy was again demonstrated, as well as the possibility to use the Ugi reaction with glycolaldehyde dimer for the synthesis of β-lactam-polyphenol hybrids. Due to the wide range of biological activities of polyphenols, we plan to investigate the many compounds synthesized through this strategy on other biological targets or in phenotypic assays. Studies towards this goal are in progress.

## Figures and Tables

**Table 1:** Solubility and Thioflavin assays on compounds **8a**,**g**, **9**, **13a**,**b**, **17a**,**b.**

Entry	Compound	Solubility ^1^	Plateau Aβ1-42 ^2^	Plateau AβpE3-42 ^2^
1	control	-	100%	100%
2	**8a**	100 µM	50%	90%
3	**8b**	250 µM	65%	53%
4	**8c**	400 µM	74%	51%
5	**8d**	200 µM	68%	89%
6	**8e**	250 µM	68%	72%
7	**8f**	>500 µM	86%	192%
8	**8g**	100 µM	102%	127%
9	**8h**	200 µM	117%	72%
10	**9**	100 µM	136%	104%
11	**13a**	150 µM	87%	105%
12	**13b**	100 µM	86%	77%
13	**17a**	250 µM	96%	96%
14	**17b**	250 µM	70%	72%

^1^ Concentration where the absorbance curve at about 330 nm starts to deflect from linearity and where the curve of turbidimetry at 405 nm starts to increase. ^2^ ThT Fluorescence in percentage respect to the control sample, after 24 h of aggregation at 37 °C, the concentration was 5 µM for β-amyloids and 25 µM for polyphenols in PBS + 1% DMSO.

## Supplementary Materials

The following are available online: copies of all NMR spectra.

## References

[1] Abbas M., Saeed F., Anjum F.M., Afzaal M., Tufail T., Bashir M.S., Ishtiaq A., Hussain S., Suleria H.A.R. (2017). Natural polyphenols: An overview. Int. J. Food Prop..

[2] Ganesan K., Xu B.J. (2017). A critical review on polyphenols and health benefits of black soybeans. Nutrients.

[3] Quideau S., Deffieux D., Douat-Casassus C., Pouysegu L. (2011). Plant polyphenols: Chemical properties, biological activities, and synthesis. Angew. Chem. Int. Ed. Engl..

[4] De Lucia D., Lucio O.M., Musio B., Bender A., Listing M., Dennhardt S., Koeberle A., Garscha U., Rizzo R., Manfredini S. (2015). Design, synthesis and evaluation of semi-synthetic triazole-containing caffeic acid analogues as 5-lipoxygenase inhibitors. Eur. J. Med. Chem..

[5] Imai K., Nakanishi I., Ohkubo K., Ohba Y., Arai T., Mizuno M., Fukuzumi S., Matsumoto K., Fukuhara K. (2017). Synthesis of methylated quercetin analogues for enhancement of radical-scavenging activity. RSC Adv..

[6] Khandelwal A., Hall J.A., Blagg B.S.J. (2013). Synthesis and structure-activity relationships of EGCG analogues, a recently identified Hsp90 inhibitor. J. Org. Chem..

[7] Benchekroun M., Romero A., Egea J., Leon R., Michalska P., Buendia I., Jimeno M.L., Jun D., Janockova J., Sepsova V. (2016). The antioxidant additive approach for Alzheimer’s disease therapy: New ferulic (lipoic) acid plus melatonin modified tacrines as cholinesterases inhibitors, direct antioxidants, and nuclear factor (erythroid-derived 2)-like 2 activators. J. Med. Chem..

[8] Minassi A., Cicione L., Koeberle A., Bauer J., Laufer S., Werz O., Appendino G. (2012). A multicomponent carba-betti strategy to alkylidene heterodimers—Total synthesis and structure-activity relationships of arzanol. Eur. J. Org. Chem..

[9] Montanari S., Bartolini M., Neviani P., Belluti F., Gobbi S., Pruccoli L., Tarozzi A., Falchi F., Andrisano V., Miszta P. (2016). Multitarget strategy to address Alzheimer’s disease: Design, synthesis, biological evaluation, and computational studies of coumarin-based derivatives. ChemMedChem.

[10] Vo D.D., Elofsson M. (2017). Synthesis of 4-formyl-2-arylbenzofuran derivatives by PdCl(C_3_H_5_)dppb-catalyzed tandem sonogashira coupling-cyclization under microwave irradiation—Application to the synthesis of viniferifuran analogues. ChemistrySelect.

[11] Zhang Z., Su P., Li X., Song T., Chai G., Yu X., Zhang K. (2015). Novel Mcl-1/Bcl-2 uual inhibitors created by the structure-based hybridization of drug-divided building blocks and a fragment deconstructed from a known two-face BH3 mimetic. Arch. Pharm..

[12] Tassano E., Alama A., Basso A., Dondo G., Galatini A., Riva R., Banfi L. (2015). Conjugation of hydroxytyrosol with other natural phenolic fragments: From waste to antioxidants and antitumour compounds. Eur. J. Org. Chem..

[13] Espley R.V., Butts C.A., Laing W.A., Martell S., Smith H., McGhie T.K., Zhang J., Paturi G., Hedderley D., Bovy A. (2014). Dietary flavonoids from modified apple reduce inflammation markers and modulate gut microbiota in mice. J. Nutr..

[14] Daglia M., Di Lorenzo A., Nabavi S.F., Talas Z.S., Nabavi S.M. (2014). Polyphenols: Well beyond the antioxidant capacity: Gallic acid and related compounds as neuroprotective agents: You are what you eat!. Curr. Pharm. Biotech..

[15] Fresco P., Borges F., Marques M.P.M., Diniz C. (2010). The anticancer properties of dietary polyphenols and its relation with apoptosis. Curr. Pharm. Design.

[16] Coppo E., Marchese A. (2014). Antibacterial activity of polyphenols. Curr. Pharm. Biotech..

[17] Bahadoran Z., Mirmiran P., Azizi F. (2013). Dietary polyphenols as potential nutraceuticals in management of diabetes: A review. J. Diabetes Metab. Disord..

[18] Ngoungoure V.L.N., Schluesener J., Moundipa P.F., Schluesener H. (2015). Natural polyphenols binding to amyloid: A broad class of compounds to treat different human amyloid diseases. Mol. Nutr. Food Res..

[19] Liu Y., Wang S.H., Dong S.Z., Chang P., Jiang Z.F. (2015). Structural characteristics of (−)-epigallocatechin-3-gallate inhibiting amyloid A beta 42 aggregation and remodeling amyloid fibers. RSC Adv..

[20] Pate K.M., Rogers M., Reed J.W., van der Munnik N., Vance S.Z., Moss M.A. (2017). Anthoxanthin polyphenols attenuate a oligomer-induced neuronal responses associated with Alzheimer’s disease. CNS Neurosci. Therap..

[21] Randino R., Grimaldi M., Persico M., De Santis A., Cini E., Cabri W., Riva A., D’Errico G., Fattorusso C., D’Ursi A.M. (2016). Investigating the neuroprotective effects of turmeric extract: Structural interactions of beta-amyloid peptide with single curcuminoids. Sci. Rep..

[22] Over B., Wetzel S., Gruetter C., Nakai Y., Renner S., Rauh D., Waldmann H. (2013). Natural-product-derived fragments for fragment-based ligand discovery. Nat. Chem..

[23] Banfi L., Riva R., Basso A. (2010). Coupling isocyanide-based multicomponent reactions with aliphatic or acyl nucleophilic substitution processes. Synlett.

[24] Banfi L., Basso A., Lambruschini C., Moni L., Riva R. (2017). Synthesis of seven-membered nitrogen heterocycles through the Ugi multicomponent reaction. Chem. Heterocycl. Comp..

[25] Lambruschini C., Galante D., Moni L., Ferraro F., Gancia G., Riva R., Traverso A., Banfi L., D’Arrigo C. (2017). Multicomponent, fragment-based synthesis of polyphenol-containing peptidomimetics and their inhibiting activity on beta-amyloid oligomerization. Org. Biomol. Chem..

[26] Tomaselli S., Balducci C., Pagano K., Galante D., D’Arrigo C., Lambruschini L., Moni L., Banfi L., Molinari H., Forloni G. (2019). Biophysical and in vivo studies identify a new natural-based polyphenol, counteracting Aβ oligomerization in vitro and Aβ oligomer-mediated memory impairment and neuroinflammation in an acute mouse model of Alzheimer’s disease. ACS Chem. Neurosci..

[27] Banfi L., Basso A., Guanti G., Lecinska P., Riva R. (2008). Multicomponent synthesis of benzoxazinones via tandem Ugi/Mitsunobu reactions: An unexpected cine-substitution. Mol. Div..

[28] Kim Y.B., Choi E.H., Keum G., Kang S.B., Lee D.H., Koh H.Y., Kim Y.S. (2001). An efficient synthesis of morpholin-2-one derivatives using glycolaldehyde dimer by the Ugi multicomponent reaction. Org. Lett..

[29] Mossetti R., Pirali T., Tron G.C. (2009). Synthesis of passerini-Ugi hybrids by a four-component reaction using the glycolaldehyde dimer. J. Org. Chem..

[30] Hanessian S., Couture C., Wiss H. (1985). Design and reactivity of organic functional groups–Utility of imidazolylsulfonates in the synthesis of monobactams and 3-amino nocardicinic acid. Can. J. Chem..

[31] Hanessian S., McNaughton-Smith G., Lombart H.-G., Lubell W.D. (1997). Design and synthesis of conformationally constrained amino acids as versatile scaffolds and peptide mimetics. Tetrahedron.

[32] Lambruschini C., Basso A., Moni L., Pinna A., Riva R., Banfi L. (2018). Bicyclic heterocycles from levulinic acid through a fast and operationally simple diversity-oriented multicomponent approach. Eur. J. Org. Chem..

[33] Banfi L., Basso A., Guanti G., Kielland N., Repetto C., Riva R. (2007). Ugi multicomponent reaction followed by an intramolecular nucleophilic substitution: Convergent multicomponent synthesis of 1-sulfonyl 1,4-diazepan-5-ones and of their benzo-fused derivatives. J. Org. Chem..

[34] Galante D., Ruggeri F.S., Dietler G., Pellistri F., Gatta E., Corsaro A., Florio T., Perico A., D’Arrigo C. (2016). A critical concentration of *N*-terminal pyroglutamylated amyloid beta drives the misfolding of Ab1-42 into more toxic aggregates. Int. J. Biochem. Cell Biol..

[35] Ding F., Borreguero J.M., Buldyrey S.V., Stanley H.E., Dokholyan N.V. (2003). Mechanism for the alpha-helix to beta-hairpin transition. Proteins.

[36] Sanguinetti M., Sanfilippo S., Castagnolo D., Sanglard D., Posteraro B., Donzellini G., Botta M. (2013). Novel macrocyclic amidinoureas: Potent non-azole antifungals active against wild-type and resistant Candida species. Acs Med. Chem. Lett..

[37] Galante D., Corsaro A., Florio T., Vella S., Pagano A., Sbrana F., Vassalli M., Perico A., D’Arrigo C. (2012). Differential toxicity, conformation and morphology of typical initial aggregation states of A beta 1-42 and A beta py3-42 beta-amyloids. Int. J. Biochem. Cell Biol..

[38] Nielsen L., Khurana R., Coats A., Frokjaer S., Brange J., Vyas S., Uversky V.N., Fink A.L. (2001). Effect of environmental factors on the kinetics of insulin fibril formation: Elucidation of the molecular mechanism. Biochemistry.

