# Supplementary material for: Multicomponent Synthesis of Polyphenols and Their In Vitro Evaluation as Potential β-Amyloid Aggregation Inhibitors
Source: Molecules. 2019 Jul 19;24(14):2636. doi: 10.3390/molecules24142636 (PMC6680962; doi:10.3390/molecules24142636)
Supplement: Supplementary file 1 [file molecules-24-02636-s001.pdf]

Supporting Information

Multicomponent Synthesis of Polyphenols and their *in vitro* Evaluation as Potential Inhibitors of β-Amyloid Aggregation

Denise Galante, Luca Banfi, Giulia Baruzzo, Andrea Basso, Cristina D'Arrigo, Dario Lunaccio, Lisa Moni, Renata Riva, and Chiara Lambruschini*

Contents

1. NMR Spectra of all new compounds S12

1) ^{1}H AND ^{13}C NMR SPECTRA OF ALL NEW COMPOUNDS

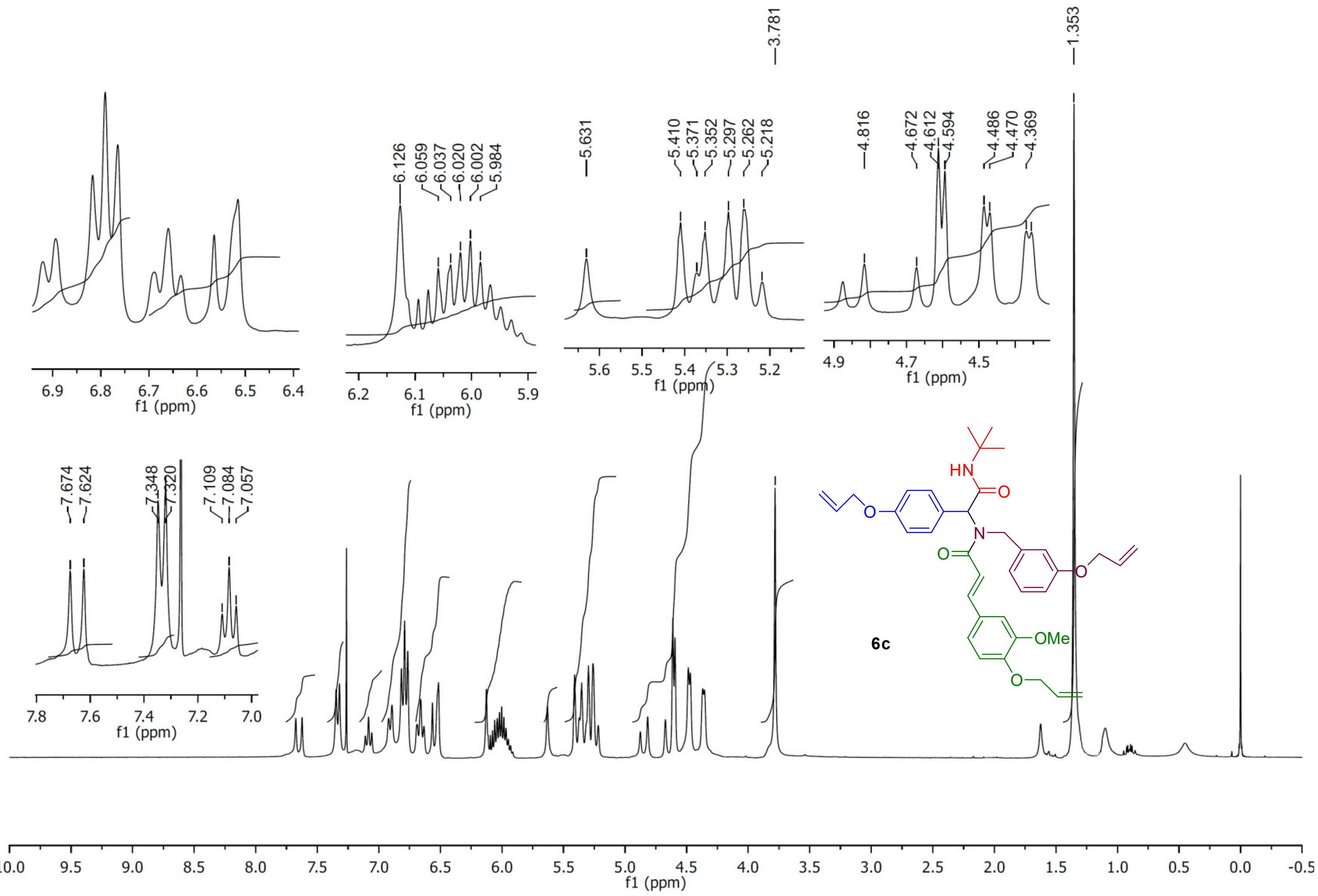

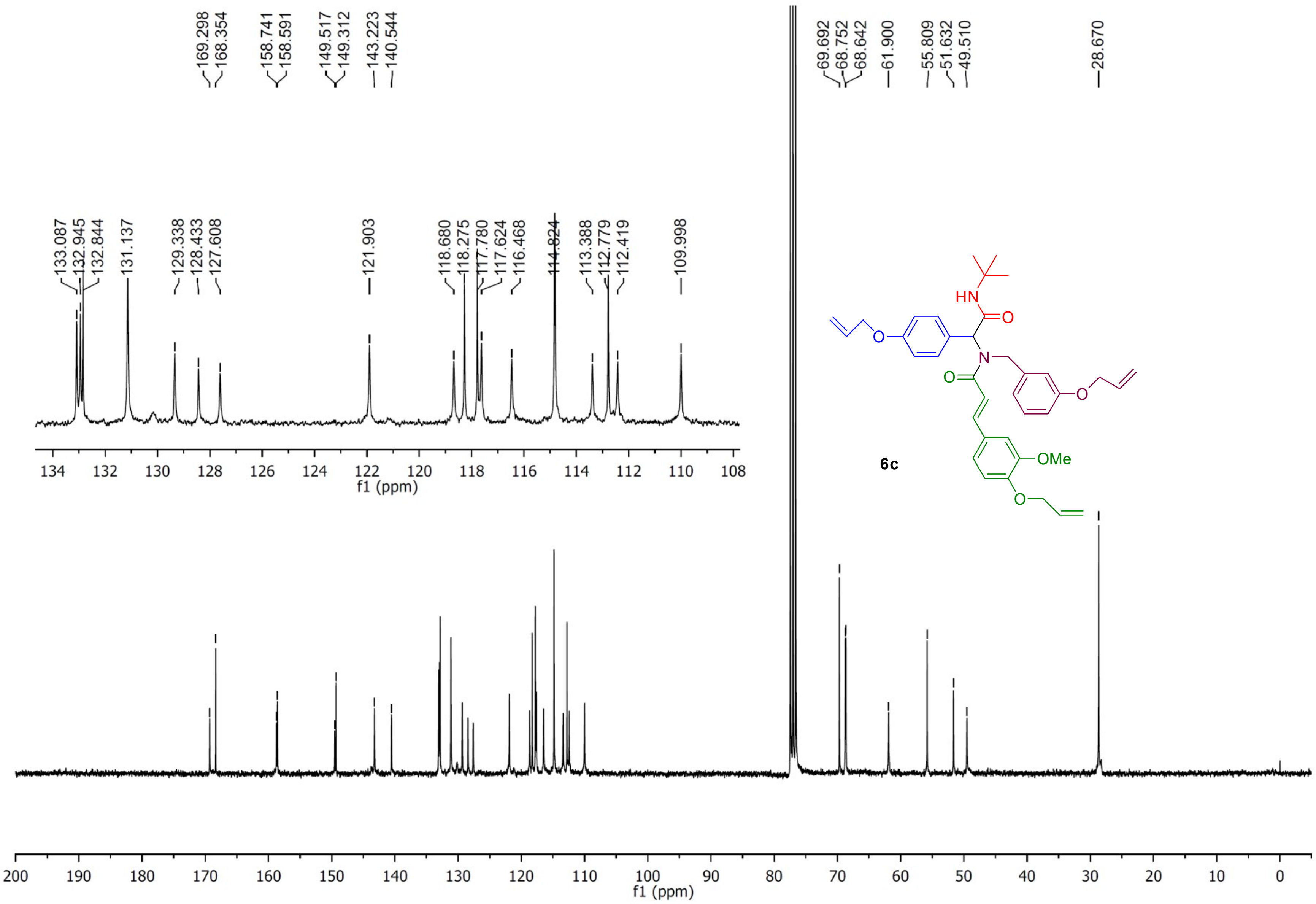
169.298
168.354
158.741
158.591
149.517
149.312
143.223
140.544
69.692
68.752
68.642
61.900
55.809
51.632
49.510
28.670
133.087
132.945
132.844
131.137
129.338
128.433
127.608
121.903
118.680
118.275
117.780
117.624
116.468
114.824
113.388
112.779
112.419
109.998
f1 (ppm)
HN
O
N
OMe
6c
f1 (ppm)

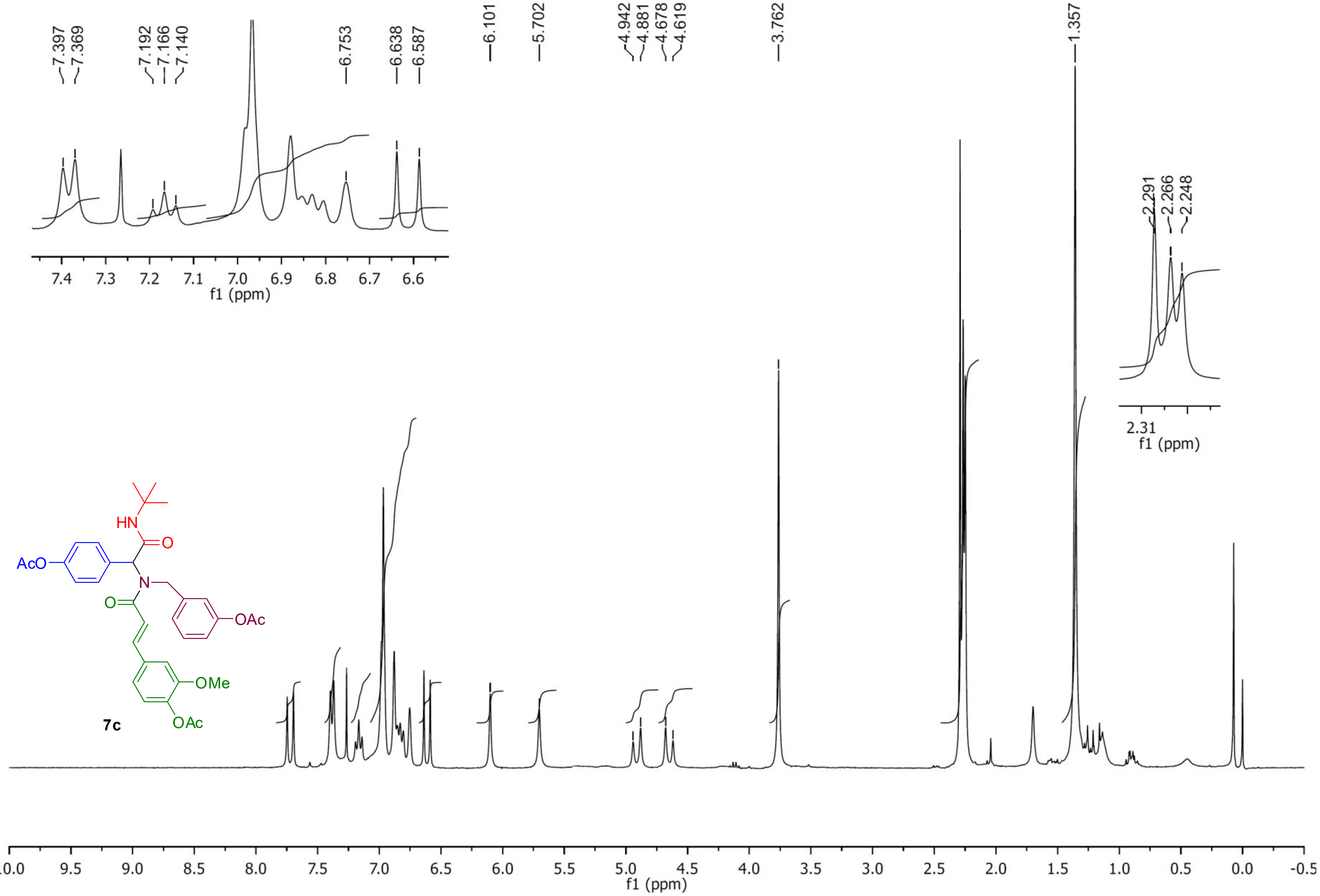

7.397
7.369
7.192
7.166
7.140
6.753
6.638
6.587
6.101
5.702
4.942
4.881
4.678
4.619
3.762
1.357
2.291
2.266
2.248
f1 (ppm)
HN
O
AcO
N
O
OAc
OMe
OAc
7c

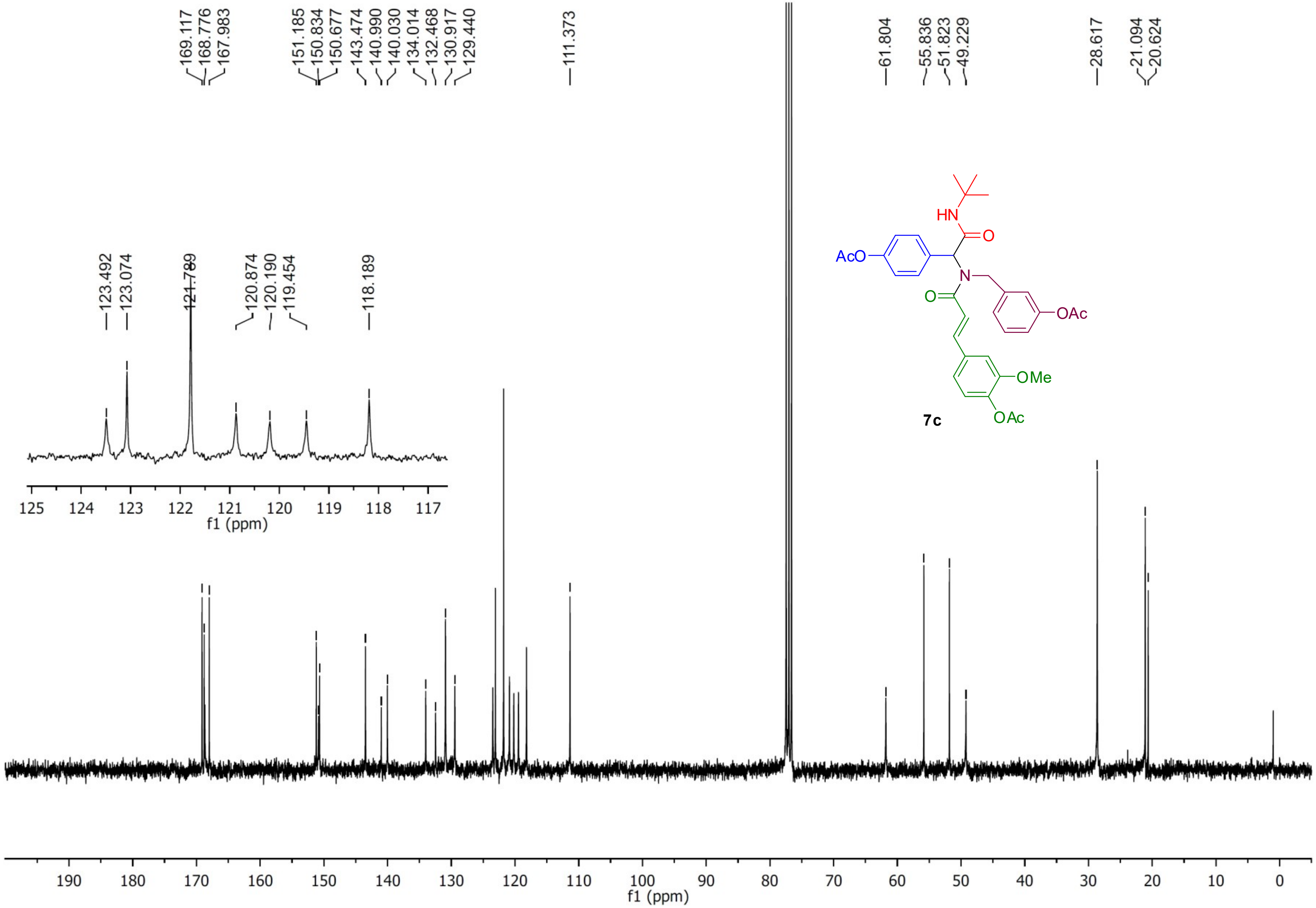
169.117
168.776
167.983
151.185
150.834
150.677
143.474
140.990
140.030
134.014
132.468
130.917
129.440
111.373
61.804
55.836
51.823
49.229
28.617
21.094
20.624
123.492
123.074
121.789
120.874
120.190
119.454
118.189
f1 (ppm)
AcO
HN
O
N
O
OAc
OMe
OAc
7c
f1 (ppm)

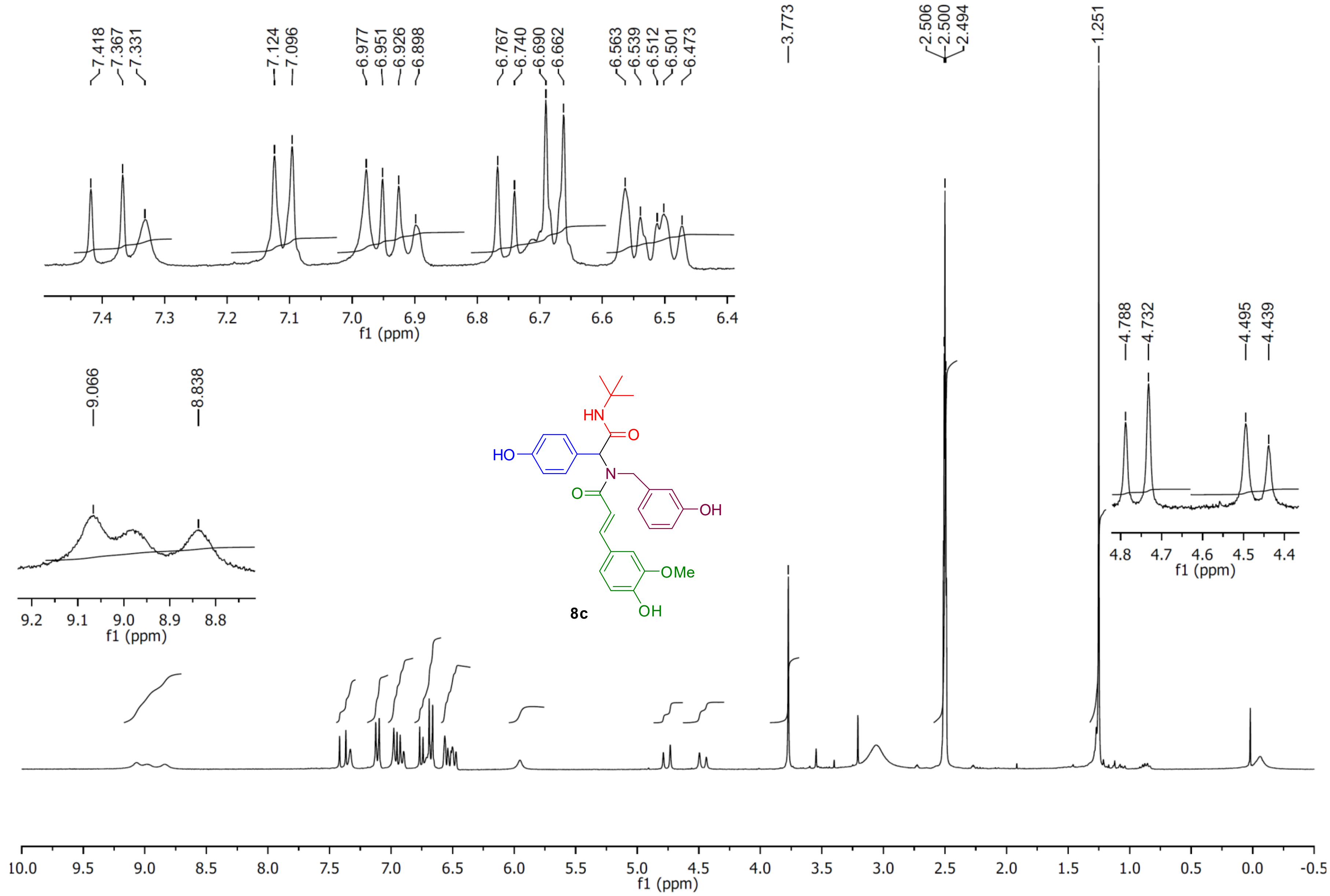
7.418
7.367
7.331
7.124
7.096
6.977
6.951
6.926
6.898
6.767
6.740
6.690
6.662
6.563
6.539
6.512
6.501
6.473
3.773
2.506
2.500
2.494
1.251
4.788
4.732
4.495
4.439
9.066
8.838
HN
O
HO
N
O
OH
OMe
OH
8c
f1 (ppm)

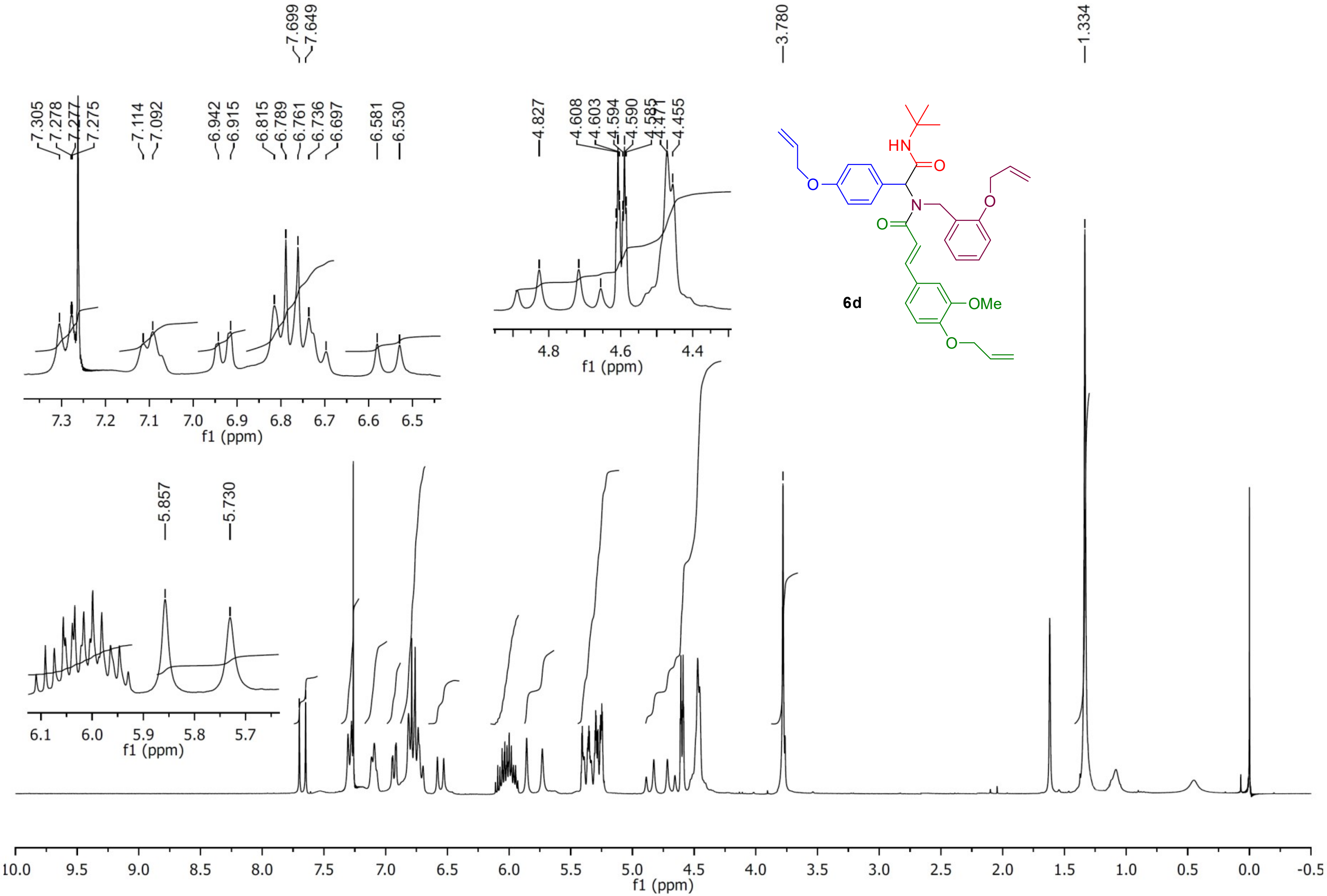

7.699
7.649
3.780
1.334
7.305
7.278
7.277
7.275
7.114
7.092
6.942
6.915
6.815
6.789
6.761
6.736
6.697
6.581
6.530
4.827
4.608
4.603
4.594
4.590
4.585
4.471
4.455
5.857
5.730
HN
O
N
O
O
OMe
O
O
6d
f1 (ppm)

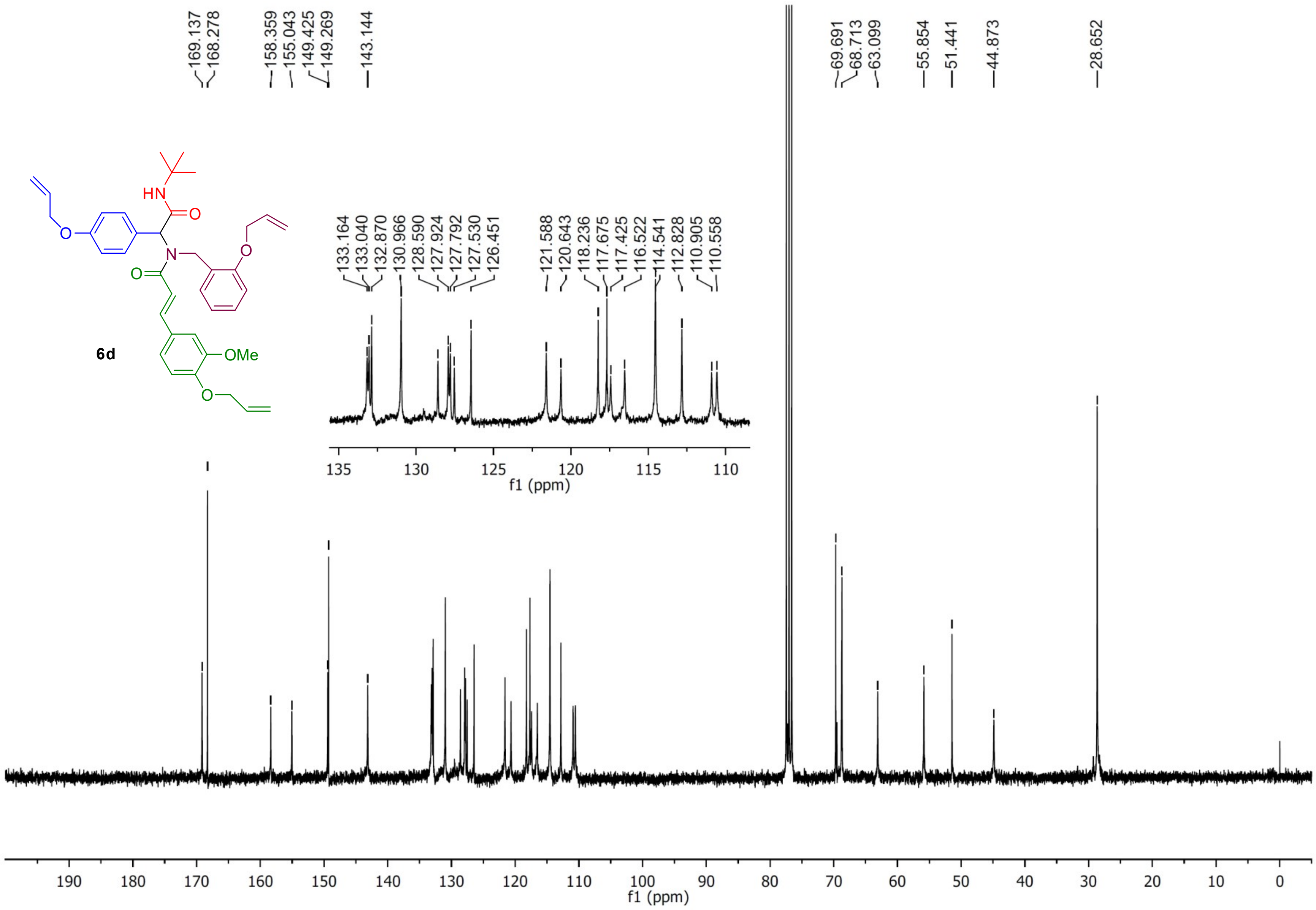

169.137
168.278
158.359
155.043
149.425
149.269
143.144
69.691
68.713
63.099
55.854
51.441
44.873
28.652
HN
O
N
O
O
O
OMe
O
6d
133.164
133.040
132.870
130.966
128.590
127.924
127.792
127.530
126.451
121.588
120.643
118.236
117.675
117.425
116.522
114.541
112.828
110.905
110.558
135 130 125 120 115 110
f1 (ppm)
190 180 170 160 150 140 130 120 110 100 90 80 70 60 50 40 30 20 10 0
f1 (ppm)

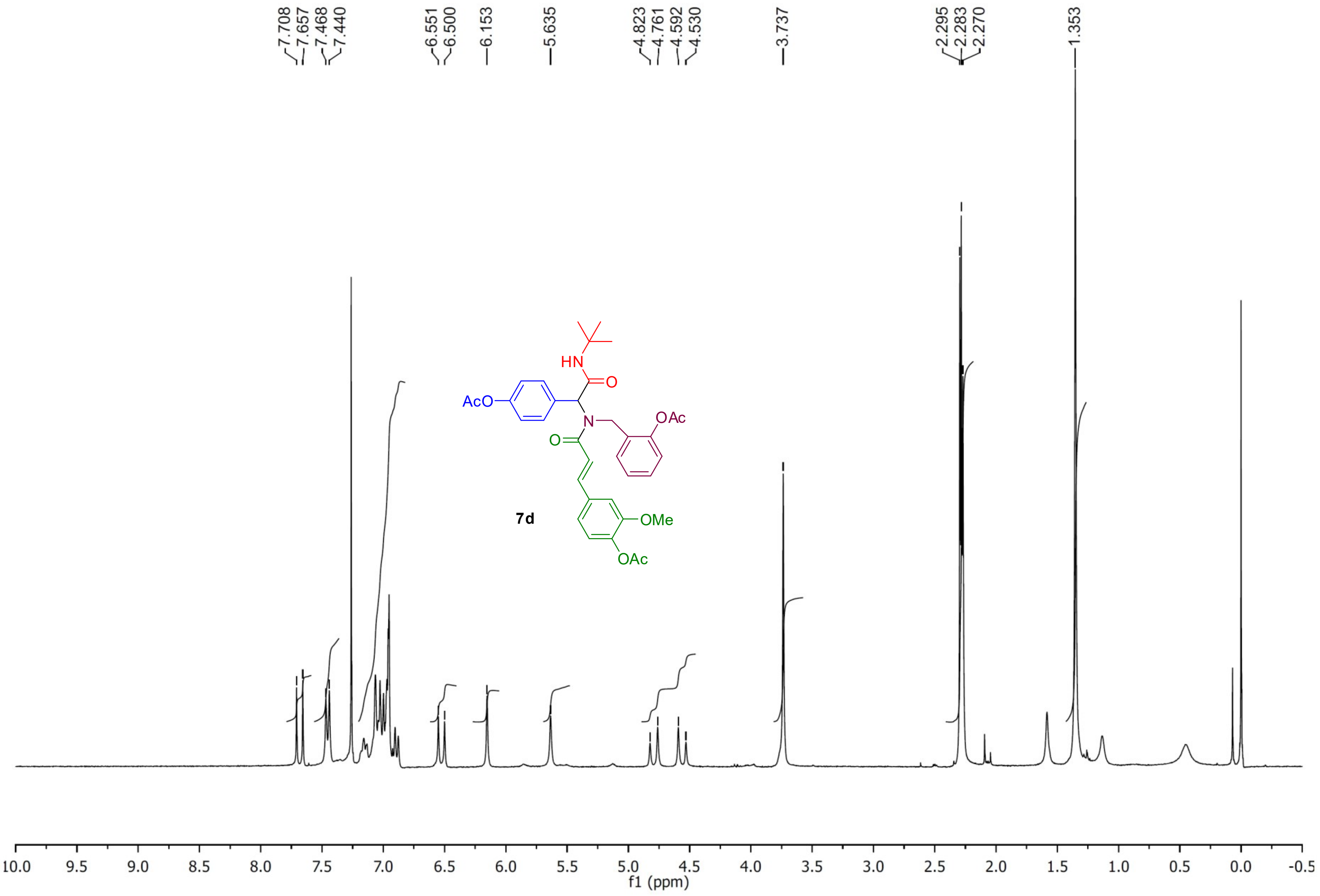

7.708
7.657
7.468
7.440
6.551
6.500
6.153
5.635
4.823
4.761
4.592
4.530
3.737
2.295
2.283
2.270
1.353
HN
O
AcO
N
OAc
O
7d
OMe
OAc
10.0
9.5
9.0
8.5
8.0
7.5
7.0
6.5
6.0
5.5
5.0
4.5
4.0
3.5
3.0
2.5
2.0
1.5
1.0
0.5
0.0
-0.5
f1 (ppm)

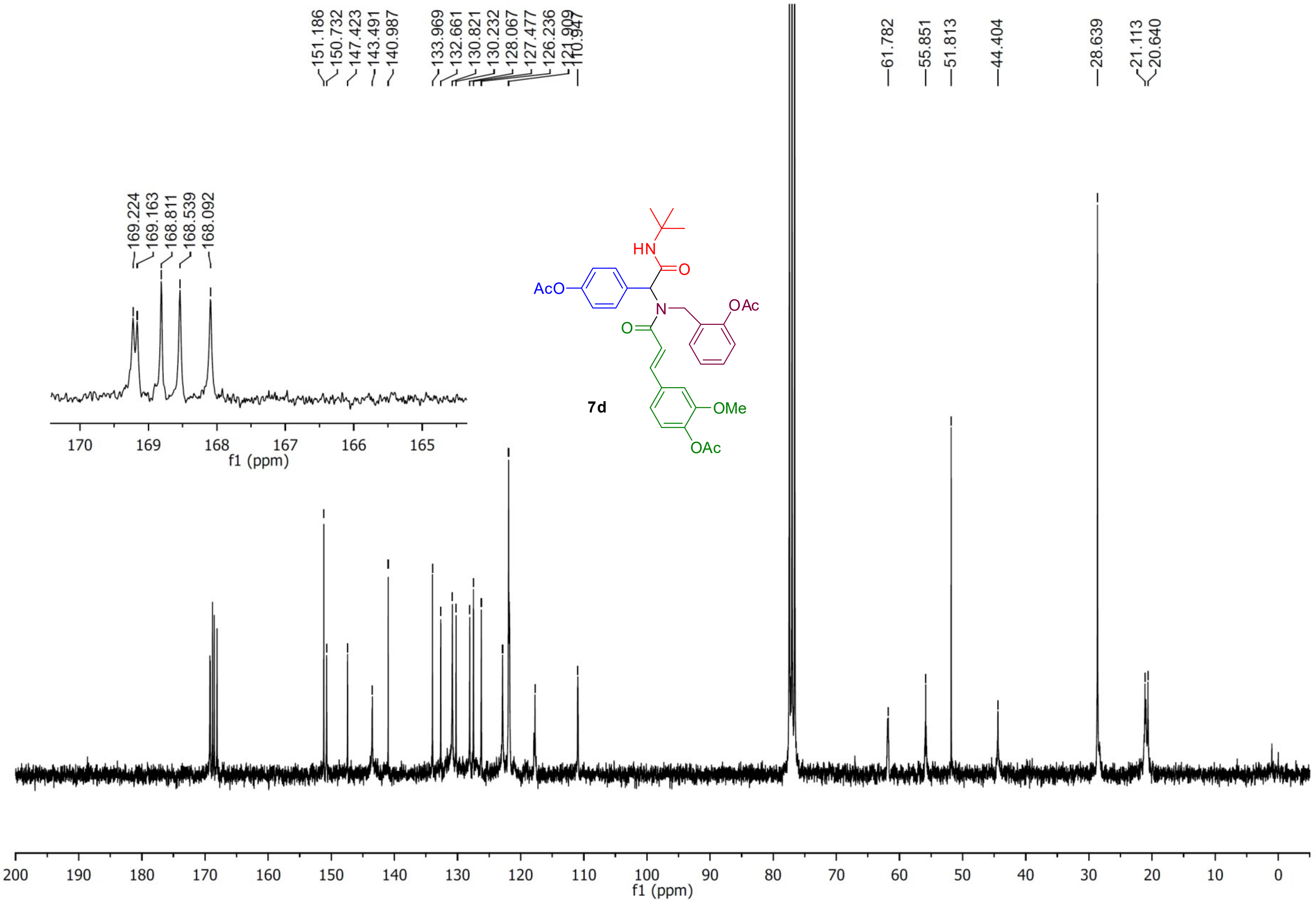
151.186
150.732
147.423
143.491
140.987
133.969
132.661
130.821
130.232
128.067
127.477
126.236
121.909
110.947
61.782
55.851
51.813
44.404
28.639
21.113
20.640
169.224
169.163
168.811
168.539
168.092
170 169 168 167 166 165
f1 (ppm)
HN
O
AcO
N
OAc
O
OMe
OAc
7d
200 190 180 170 160 150 140 130 120 110 100 90 80 70 60 50 40 30 20 10 0
f1 (ppm)

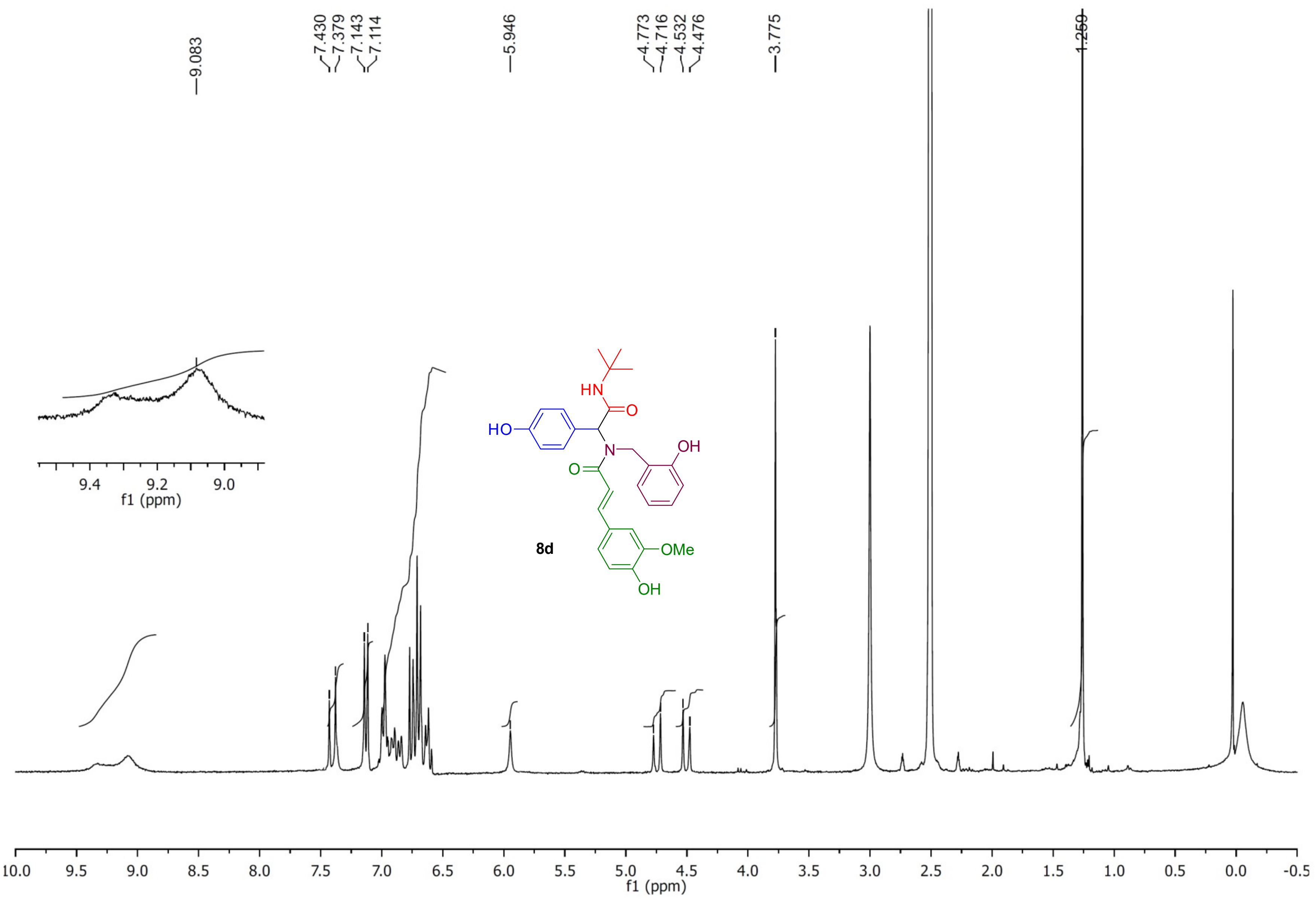
9.083
7.430
7.379
7.143
7.114
5.946
4.773
4.716
4.532
4.476
3.775
1.259
HN
O
HO
N
OH
O
OMe
OH
8d
9.4
9.2
9.0
f1 (ppm)
10.0
9.5
9.0
8.5
8.0
7.5
7.0
6.5
6.0
5.5
5.0
4.5
4.0
3.5
3.0
2.5
2.0
1.5
1.0
0.5
0.0
-0.5
f1 (ppm)

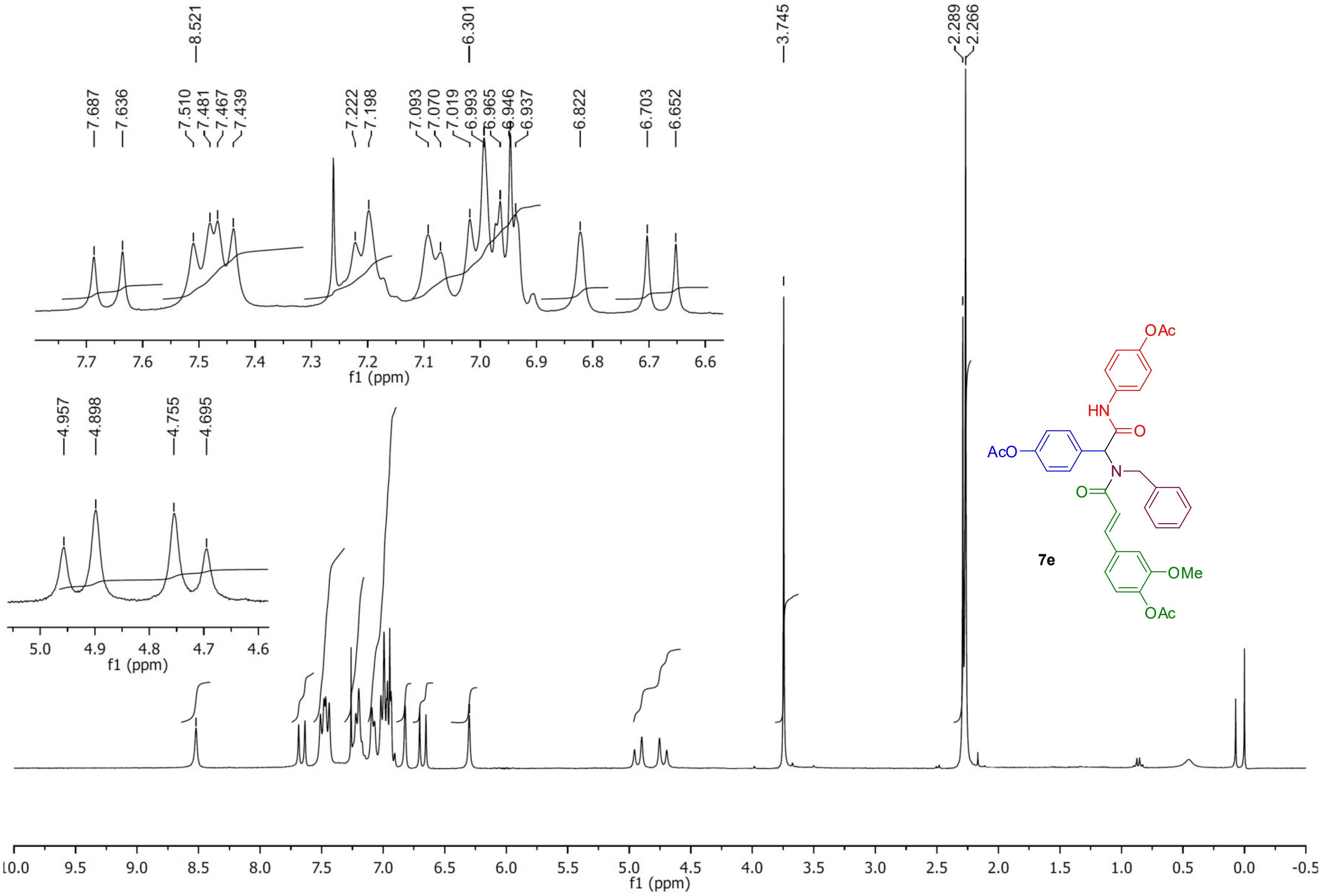
8.521
7.687
7.636
7.510
7.481
7.467
7.439
7.222
7.198
7.093
7.070
7.019
6.993
6.965
6.946
6.937
6.822
6.703
6.652
6.301
3.745
2.289
2.266
4.957
4.898
4.755
4.695
f1 (ppm)
OAc
HN
O
AcO
N
O
OMe
OAc
7e

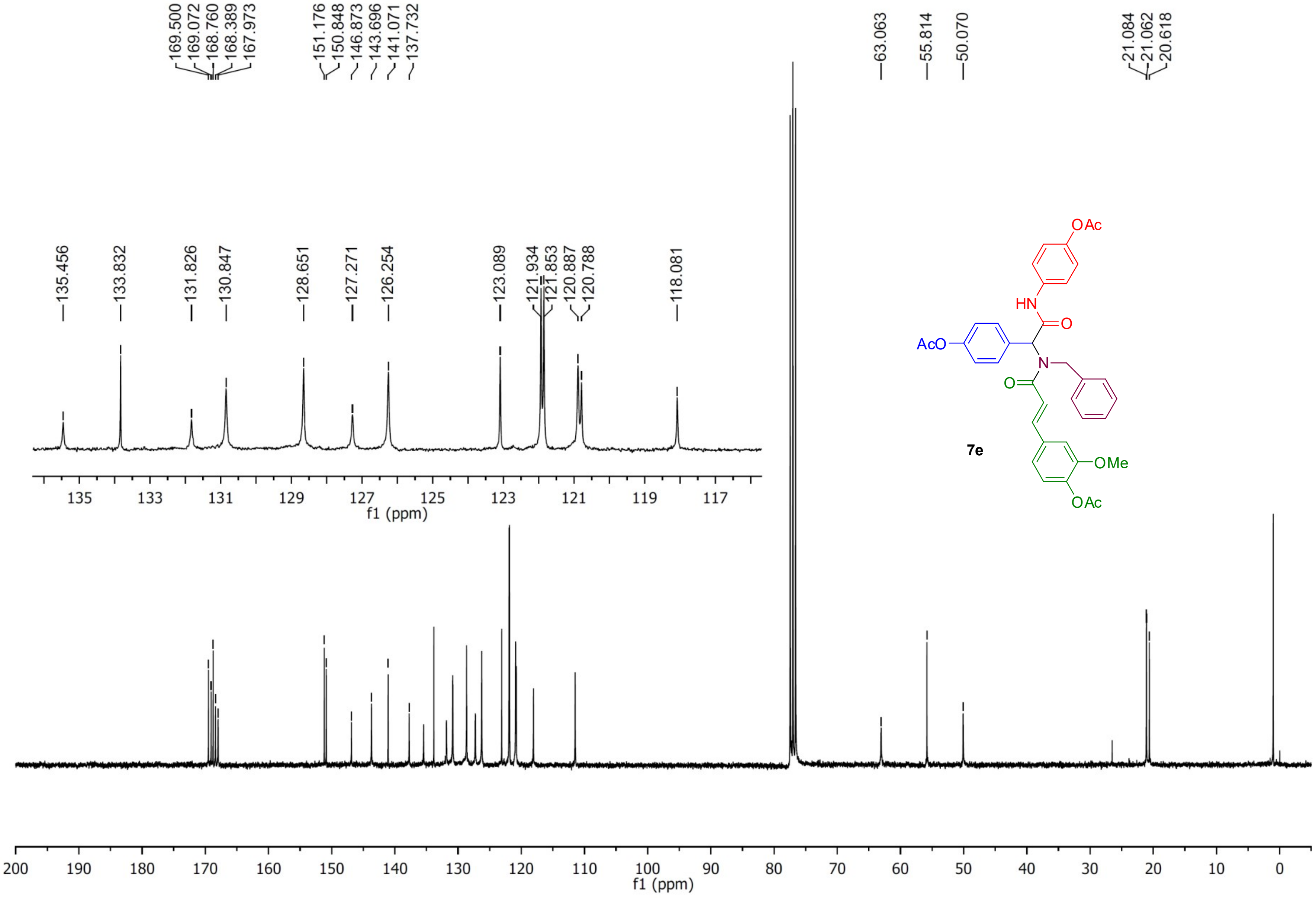
169.500
169.072
168.760
168.389
167.973
151.176
150.848
146.873
143.696
141.071
137.732
63.063
55.814
50.070
21.084
21.062
20.618
135.456
133.832
131.826
130.847
128.651
127.271
126.254
123.089
121.934
121.853
120.887
120.788
118.081
f1 (ppm)
OAc
HN
O
AcO
N
O
OMe
OAc
7e
f1 (ppm)

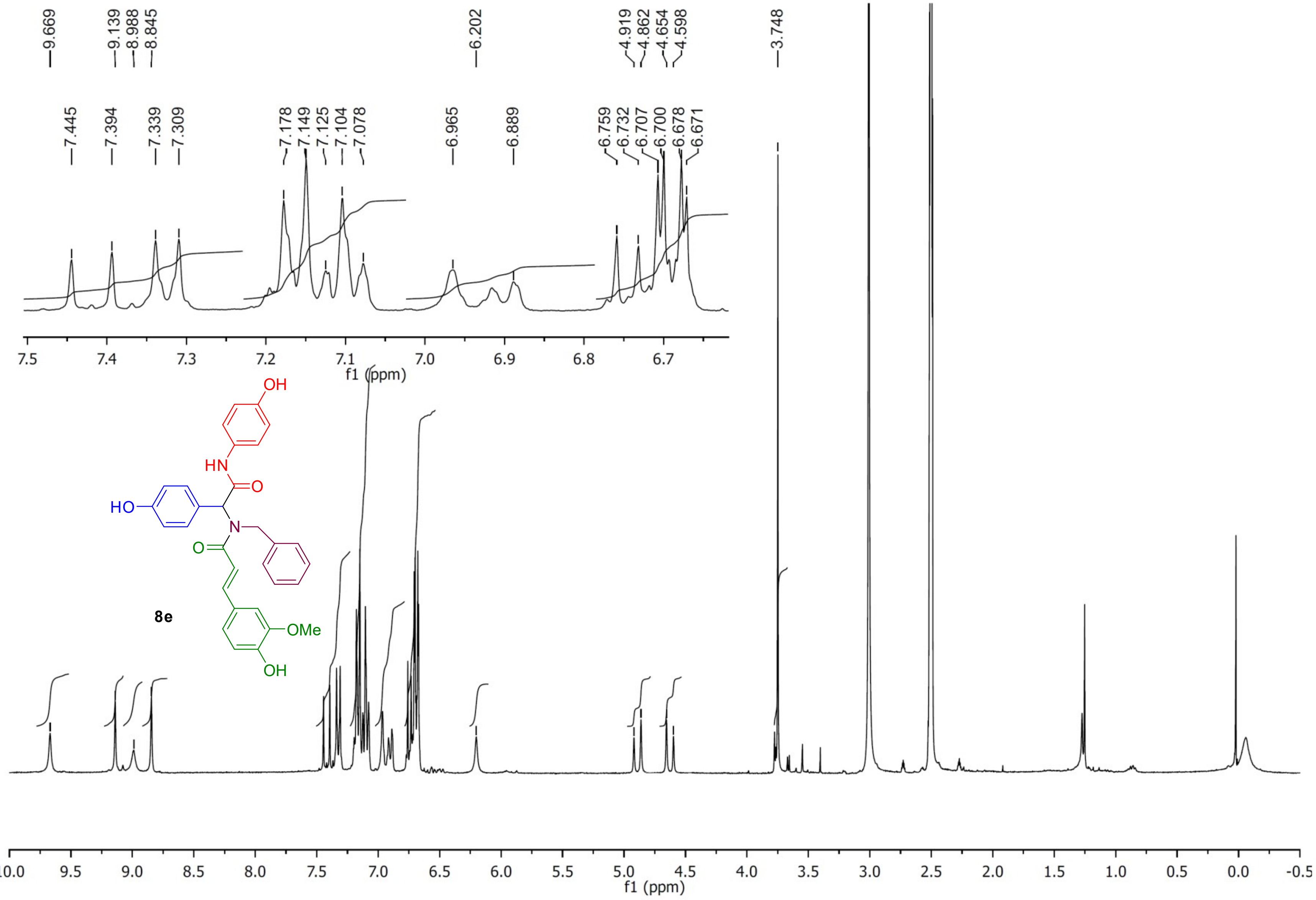
9.669
9.139
8.988
8.845
6.202
4.919
4.862
4.654
4.598
3.748
7.445
7.394
7.339
7.309
7.178
7.149
7.125
7.104
7.078
6.965
6.889
6.759
6.732
6.707
6.700
6.678
6.671
f1 (ppm)
OH
HN
O
HO
N
O
8e
OMe
OH
f1 (ppm)

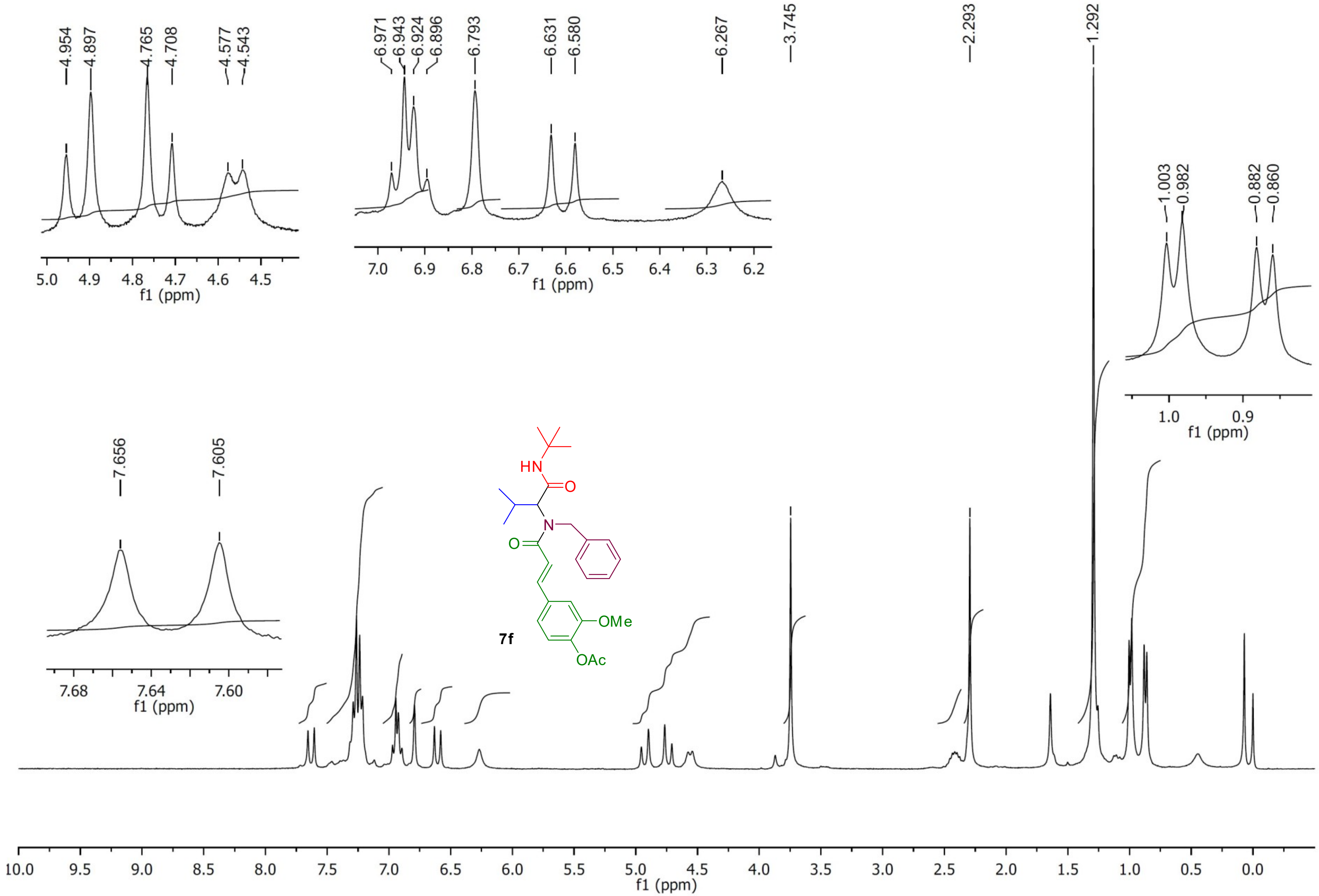

4.954
4.897
4.765
4.708
4.577
4.543
6.971
6.943
6.924
6.896
6.793
6.631
6.580
6.267
3.745
2.293
1.292
1.003
0.982
0.882
0.860
7.656
7.605
HN
O
N
O
OMe
OAc
7f
f1 (ppm)

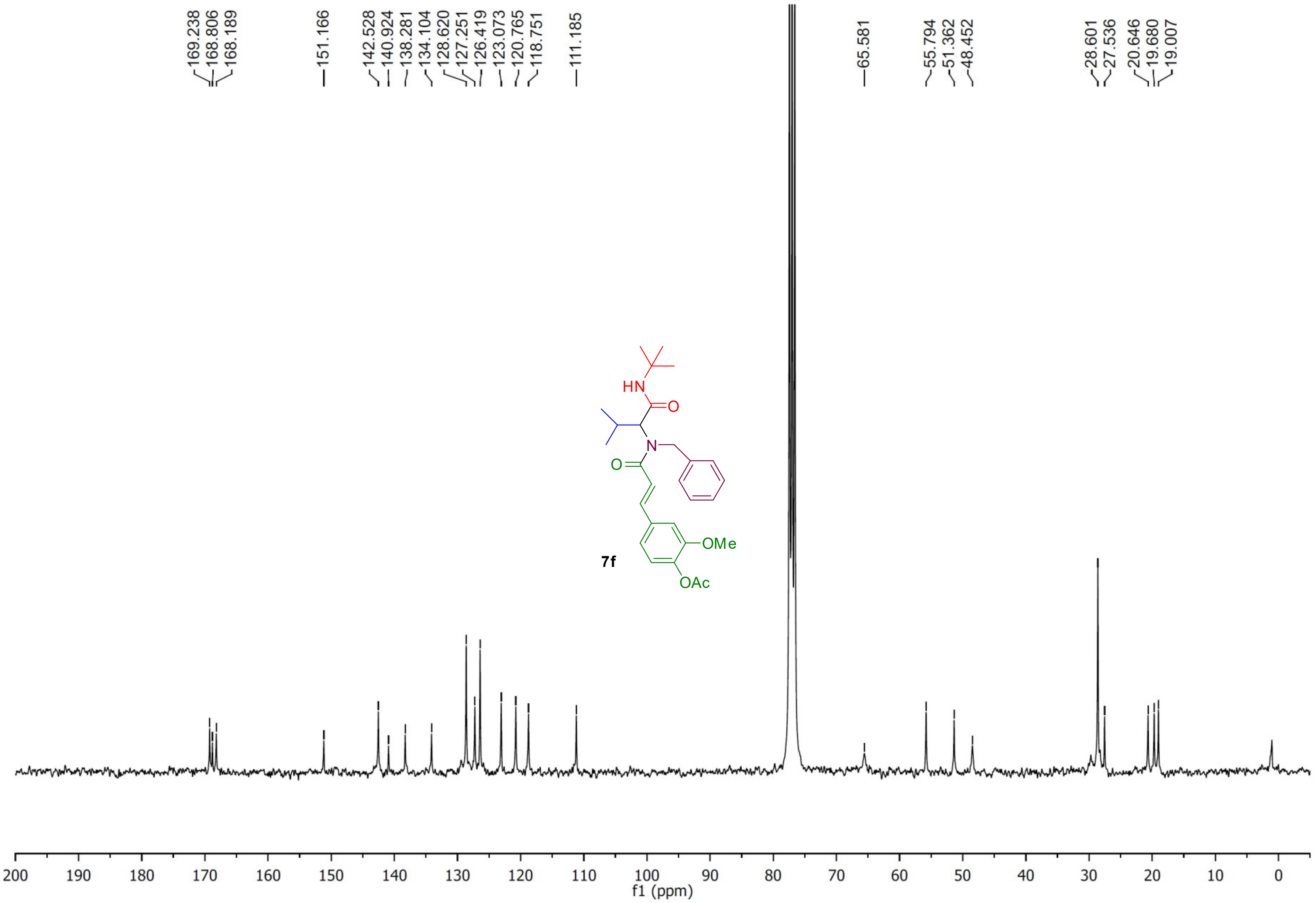
169.238
168.806
168.189
151.166
142.528
140.924
138.281
134.104
128.620
127.251
126.419
123.073
120.765
118.751
111.185
65.581
55.794
51.362
48.452
28.601
27.536
20.646
19.680
19.007
HN
O
N
O
OMe
OAc
7f
200 190 180 170 160 150 140 130 120 110 100 90 80 70 60 50 40 30 20 10 0
f1 (ppm)

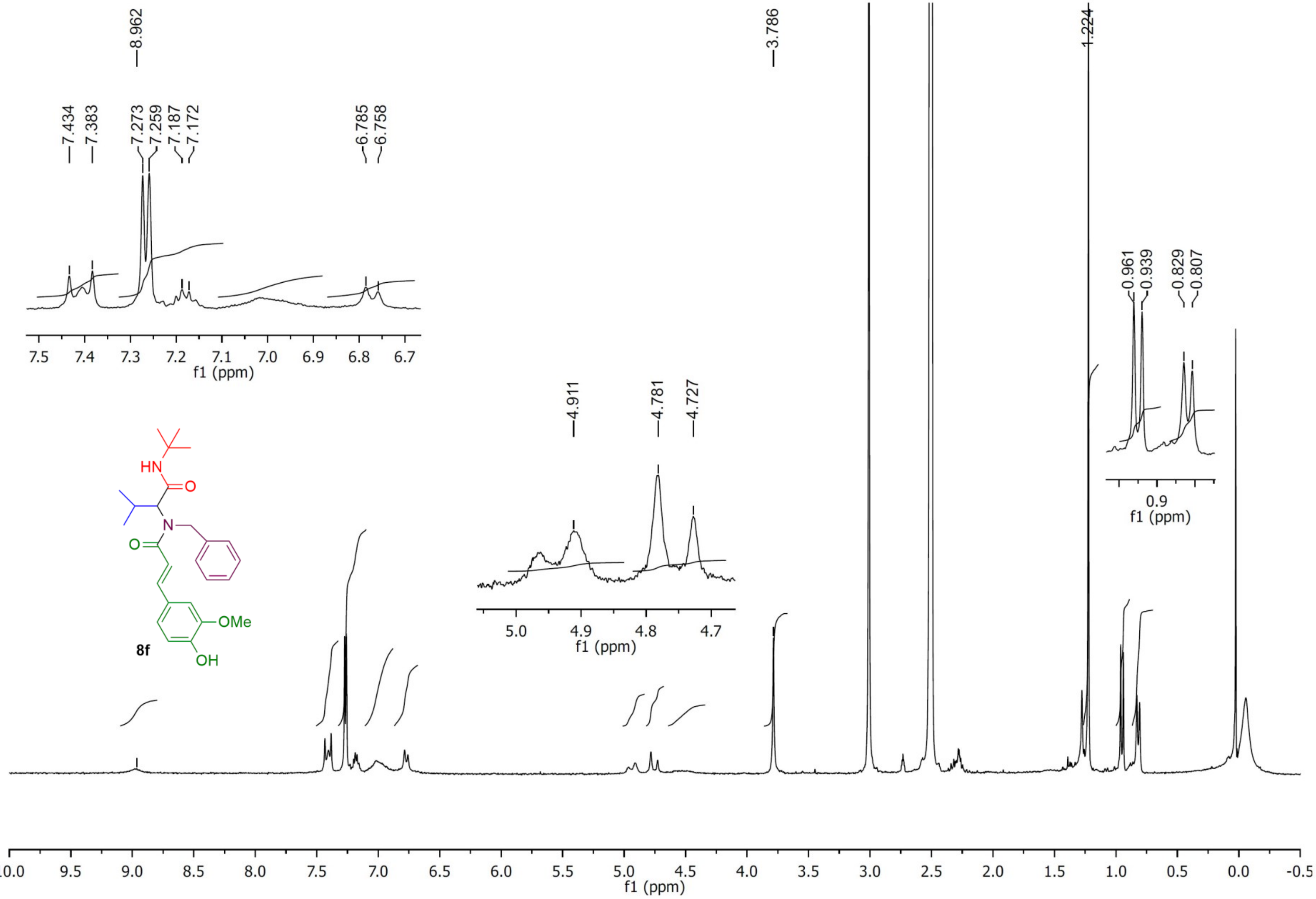
8.962
7.434
7.383
7.273
7.259
7.187
7.172
6.785
6.758
f1 (ppm)
3.786
1.224
0.961
0.939
0.829
0.807
4.911
4.781
4.727
HN
O
N
OMe
OH
8f
f1 (ppm)

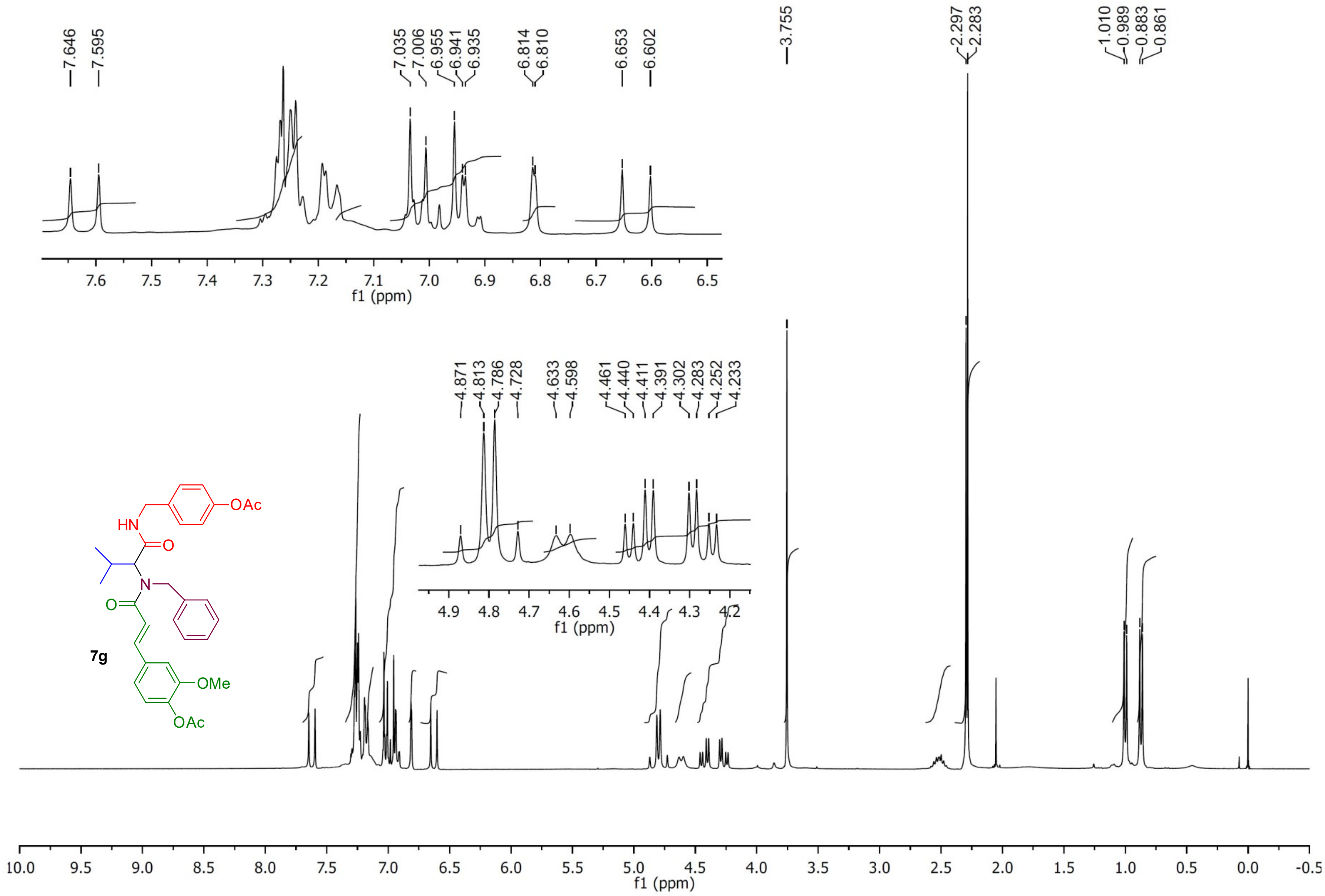
7.646
7.595
7.035
7.006
6.955
6.941
6.935
6.814
6.810
6.653
6.602
3.755
2.297
2.283
1.010
0.989
0.883
0.861
4.871
4.813
4.786
4.728
4.633
4.598
4.461
4.440
4.411
4.391
4.302
4.283
4.252
4.233
f1 (ppm)
f1 (ppm)
f1 (ppm)
HN
OAc
O
N
O
OMe
OAc
7g

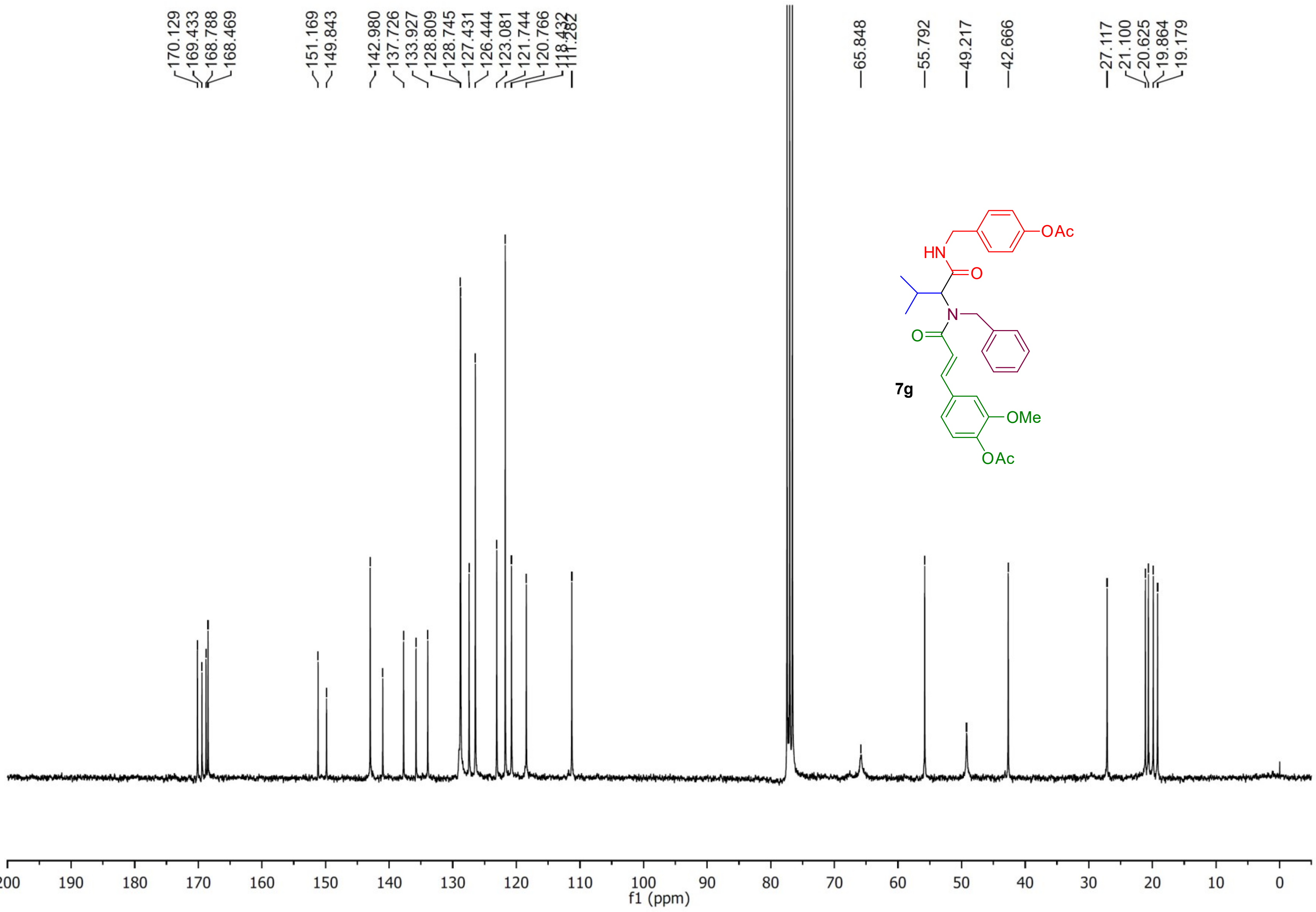
170.129
169.433
168.788
168.469
151.169
149.843
142.980
137.726
133.927
128.809
128.745
127.431
126.444
123.081
121.744
120.766
118.432
111.282
65.848
55.792
49.217
42.666
27.117
21.100
20.625
19.864
19.179
OAc
HN
O
N
O
7g
OMe
OAc
200 190 180 170 160 150 140 130 120 110 100 90 80 70 60 50 40 30 20 10 0
f1 (ppm)

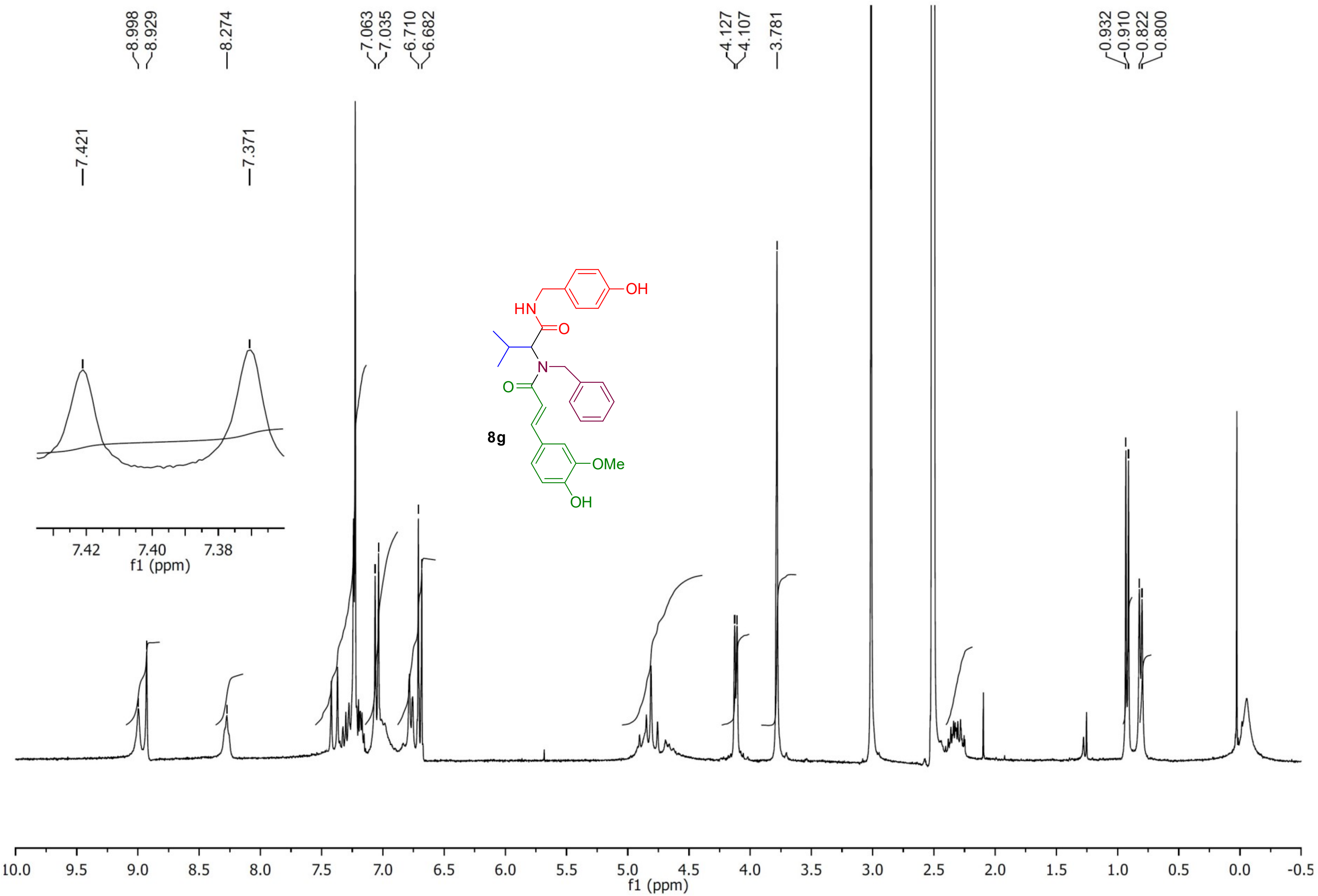

8.998
8.929
8.274
7.063
7.035
6.710
6.682
4.127
4.107
3.781
0.932
0.910
0.822
0.800
7.421
7.371
7.42
7.40
7.38
f1 (ppm)
HN
OH
O
N
O
8g
OMe
OH
10.0
9.5
9.0
8.5
8.0
7.5
7.0
6.5
6.0
5.5
5.0
4.5
4.0
3.5
3.0
2.5
2.0
1.5
1.0
0.5
0.0
-0.5
f1 (ppm)

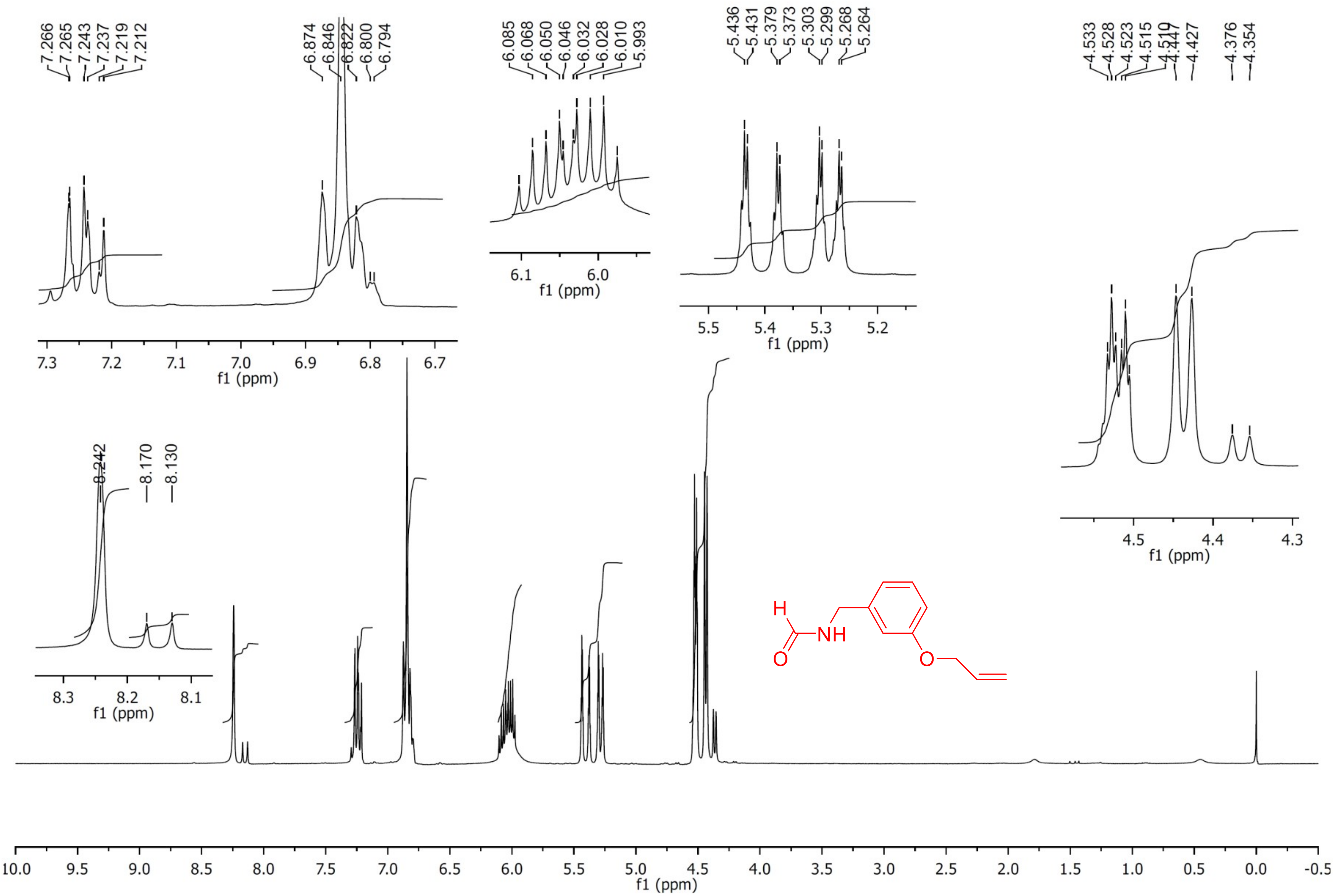

7.266
7.265
7.243
7.237
7.219
7.212
6.874
6.846
6.822
6.800
6.794
6.085
6.068
6.050
6.046
6.032
6.028
6.010
5.993
5.436
5.431
5.379
5.373
5.303
5.299
5.268
5.264
4.533
4.528
4.523
4.515
4.510
4.447
4.427
4.376
4.354
8.242
8.170
8.130
f1 (ppm)
H
NH
O
O

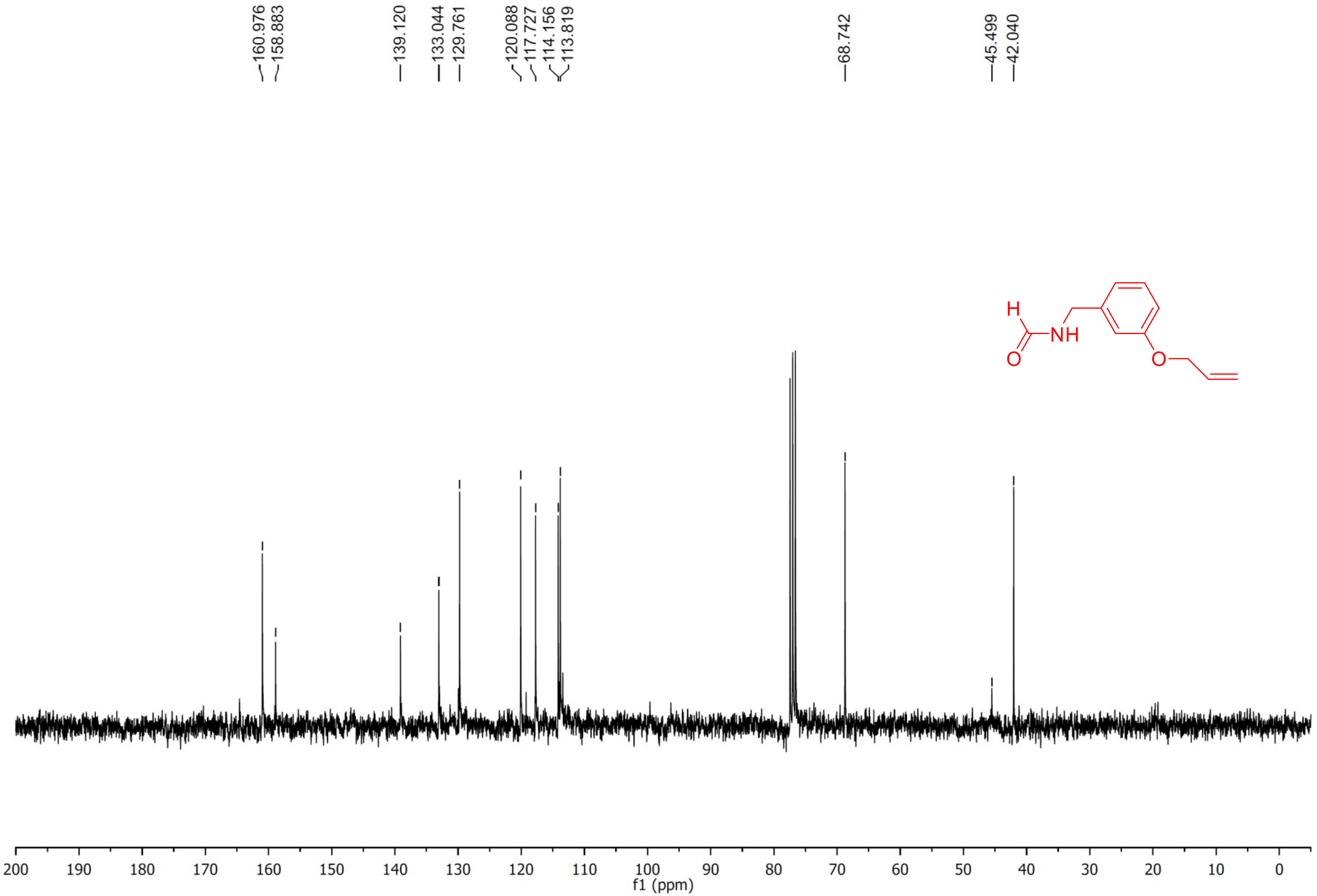

160.976
158.883
139.120
133.044
129.761
120.088
117.727
114.156
113.819
68.742
45.499
42.040
H
NH
O
O
200 190 180 170 160 150 140 130 120 110 100 90 80 70 60 50 40 30 20 10 0
f1 (ppm)

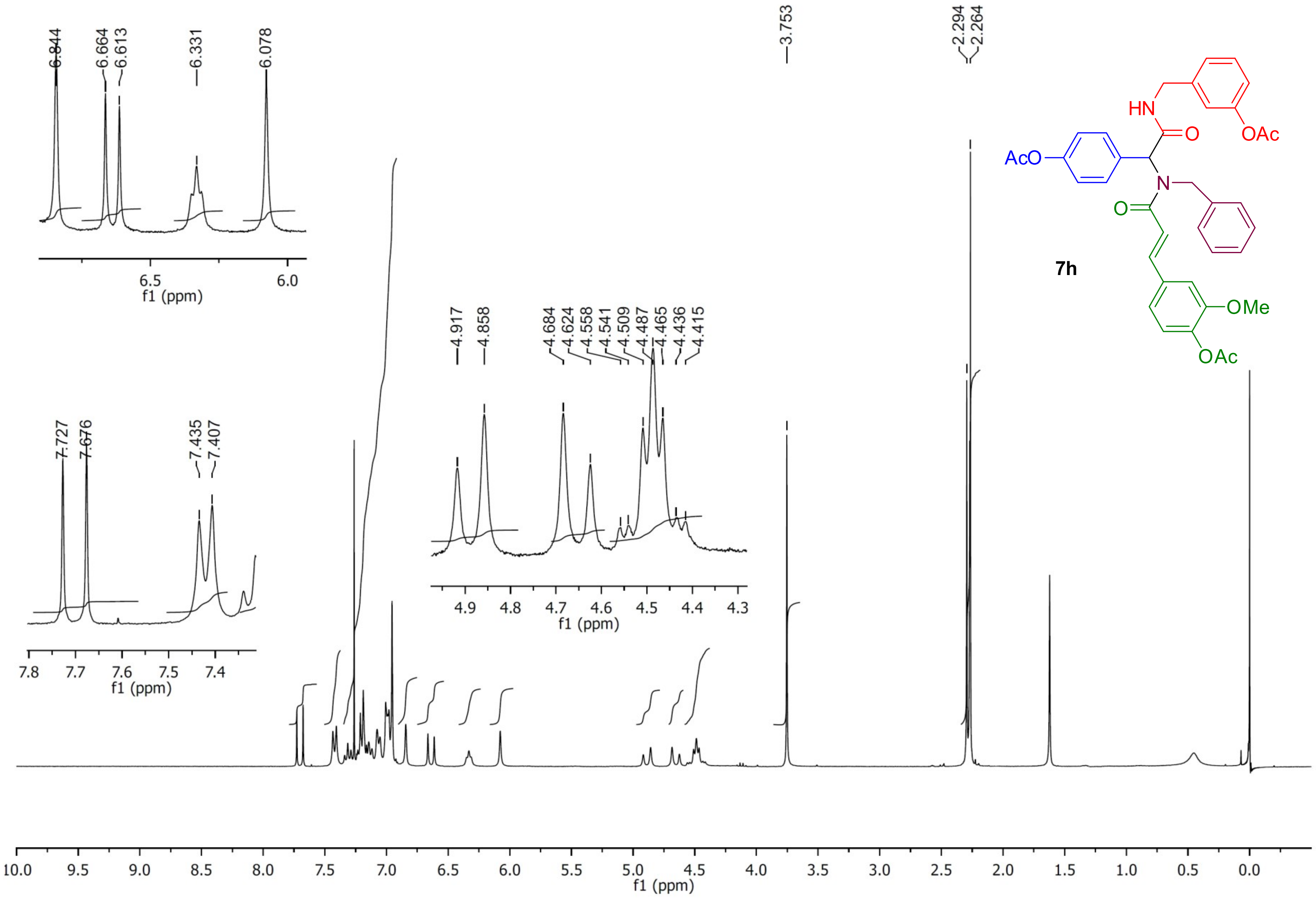

6.844
6.664
6.613
6.331
6.078
6.5
6.0
f1 (ppm)
7.727
7.676
7.435
7.407
7.8
7.7
7.6
7.5
7.4
f1 (ppm)
4.917
4.858
4.684
4.624
4.558
4.541
4.509
4.487
4.465
4.436
4.415
4.9
4.8
4.7
4.6
4.5
4.4
4.3
f1 (ppm)
3.753
2.294
2.264
HN
O
OAc
AcO
N
O
OMe
OAc
10.0
9.5
9.0
8.5
8.0
7.5
7.0
6.5
6.0
5.5
5.0
4.5
4.0
3.5
3.0
2.5
2.0
1.5
1.0
0.5
0.0
f1 (ppm)

7h

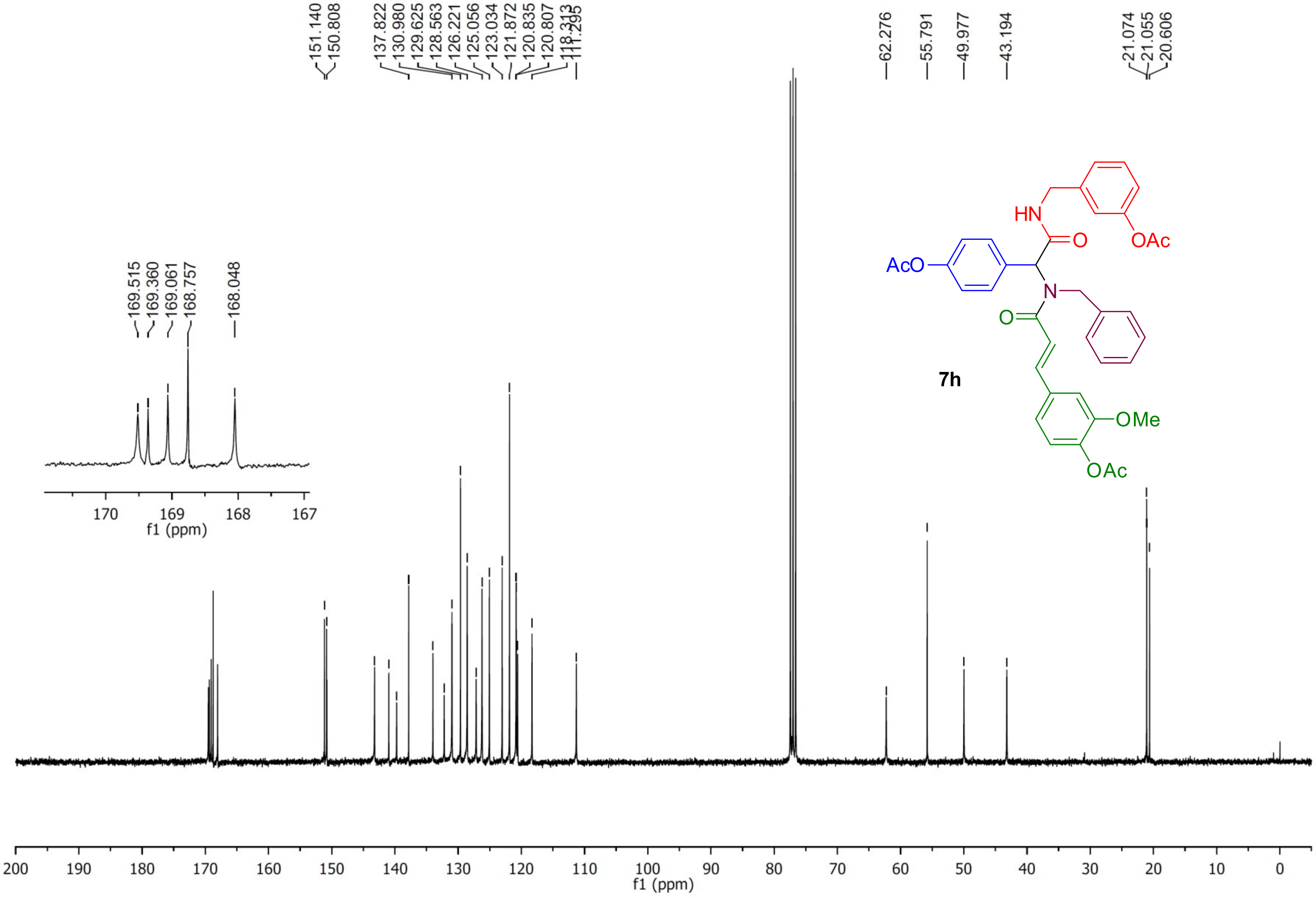
151.140
150.808
137.822
130.980
129.625
128.563
126.221
125.056
123.034
121.872
120.835
120.807
118.313
111.295
62.276
55.791
49.977
43.194
21.074
21.055
20.606
169.515
169.360
169.061
168.757
168.048
170
169
168
167
f1 (ppm)
HN
O
OAc
AcO
N
O
7h
OMe
OAc
200 190 180 170 160 150 140 130 120 110 100 90 80 70 60 50 40 30 20 10 0
f1 (ppm)

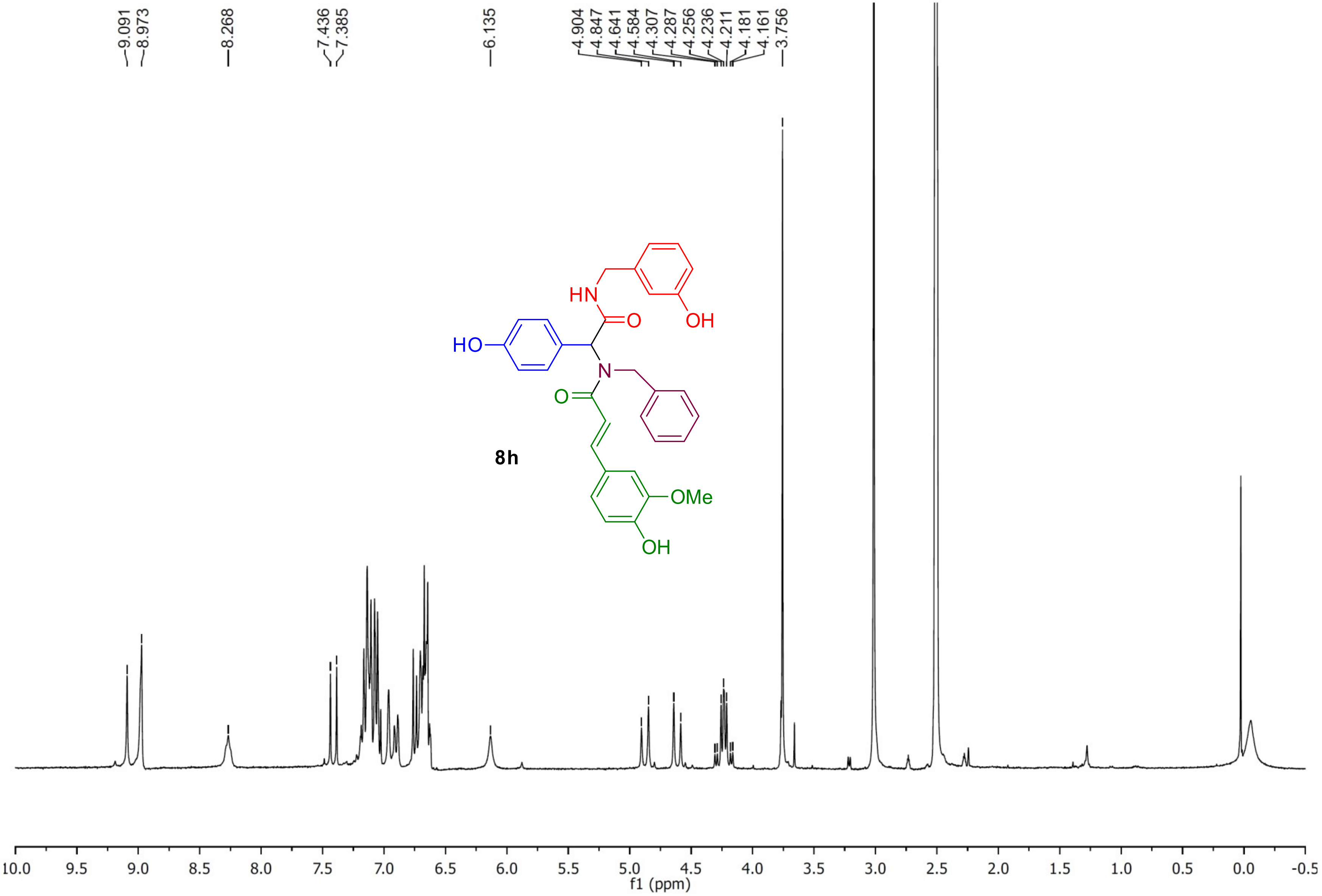

9.091
8.973
8.268
7.436
7.385
6.135
4.904
4.847
4.641
4.584
4.307
4.287
4.256
4.236
4.211
4.181
4.161
3.756
HN
O
OH
HO
N
O
8h
OMe
OH
10.0 9.5 9.0 8.5 8.0 7.5 7.0 6.5 6.0 5.5 5.0 4.5 4.0 3.5 3.0 2.5 2.0 1.5 1.0 0.5 0.0 -0.5
f1 (ppm)

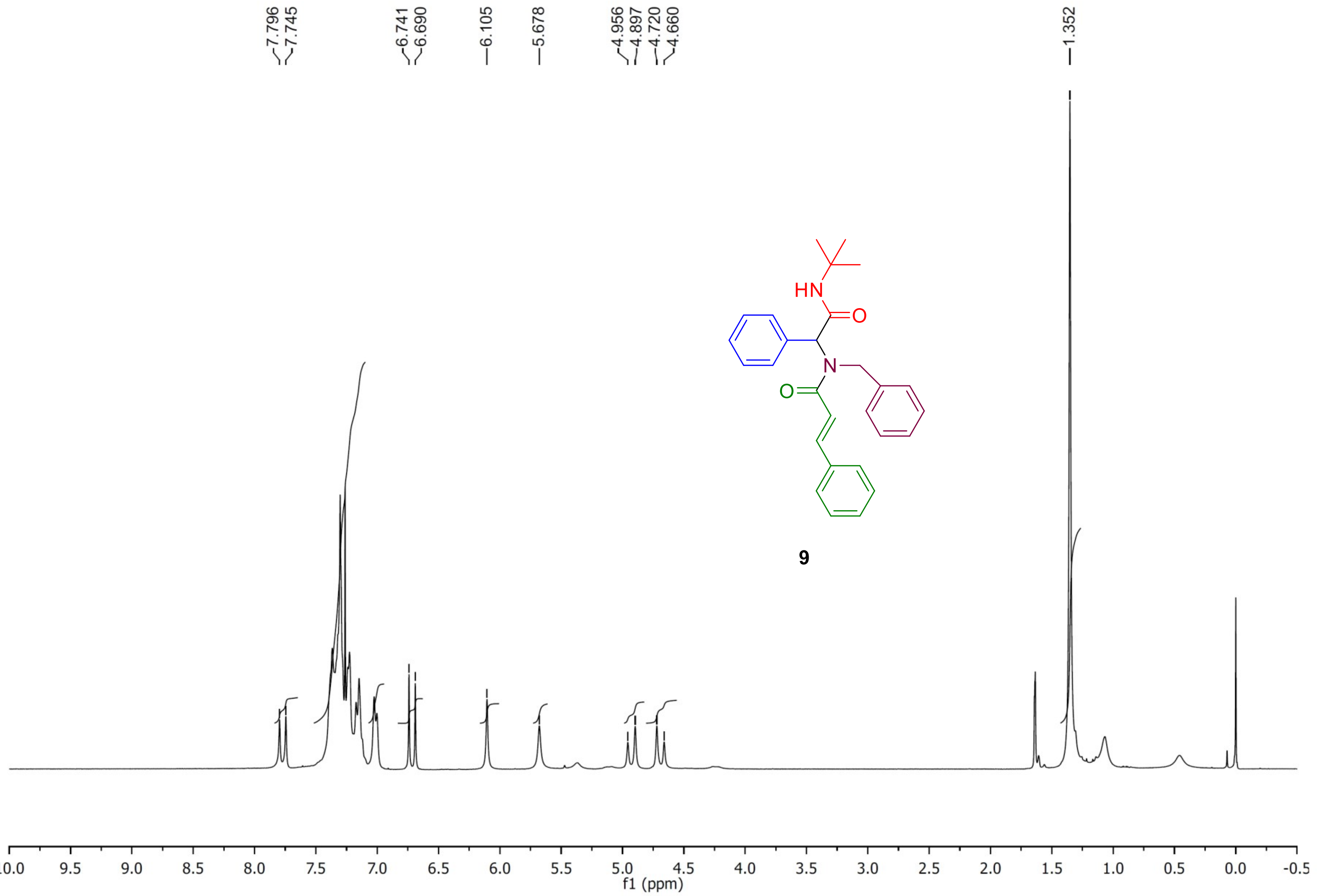
7.796
7.745
6.741
6.690
6.105
5.678
4.956
4.897
4.720
4.660
1.352
HN
O
N
O
9
10.0 9.5 9.0 8.5 8.0 7.5 7.0 6.5 6.0 5.5 5.0 4.5 4.0 3.5 3.0 2.5 2.0 1.5 1.0 0.5 0.0 -0.5
f1 (ppm)

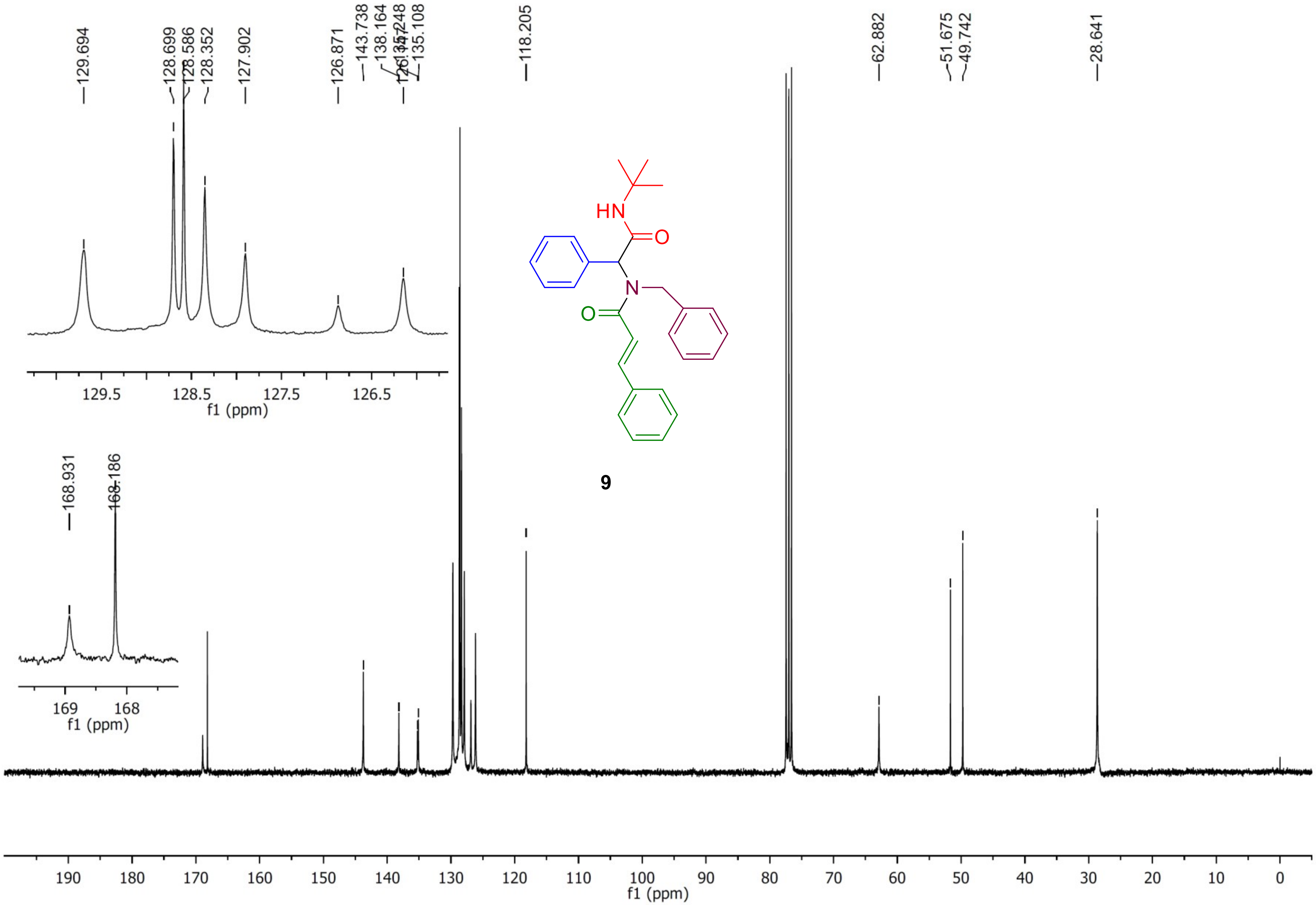

129.694
128.699
128.586
128.352
127.902
126.871
143.738
138.164
135.248
135.108
118.205
62.882
51.675
49.742
28.641
168.931
168.186
HN
O
N
O
9
f1 (ppm)

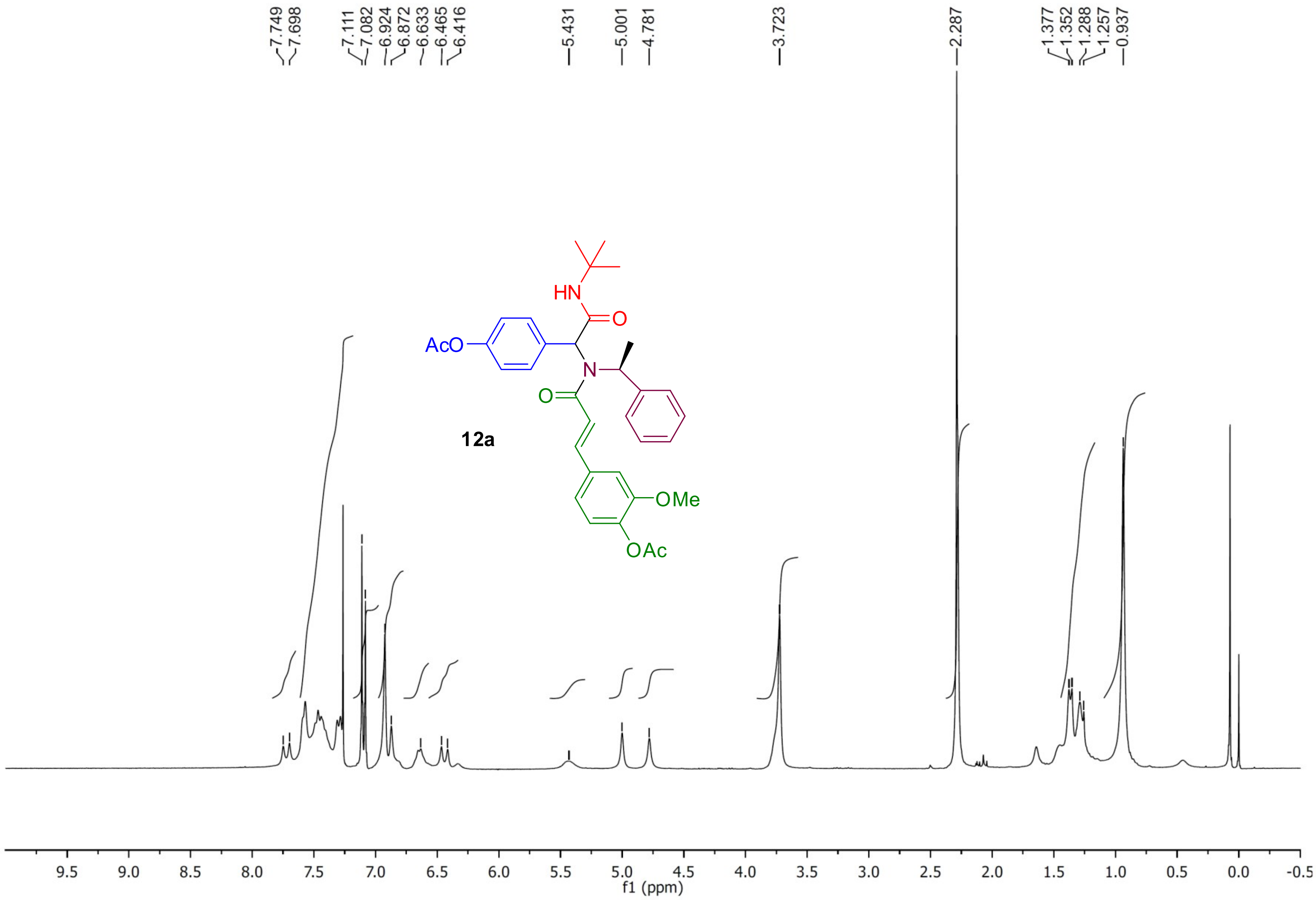

7.749
7.698
7.111
7.082
6.924
6.872
6.633
6.465
6.416
5.431
5.001
4.781
3.723
2.287
1.377
1.352
1.288
1.257
0.937
AcO
HN
O
N
O
OMe
OAc
12a
9.5
9.0
8.5
8.0
7.5
7.0
6.5
6.0
5.5
5.0
4.5
4.0
3.5
3.0
2.5
2.0
1.5
1.0
0.5
0.0
-0.5
f1 (ppm)

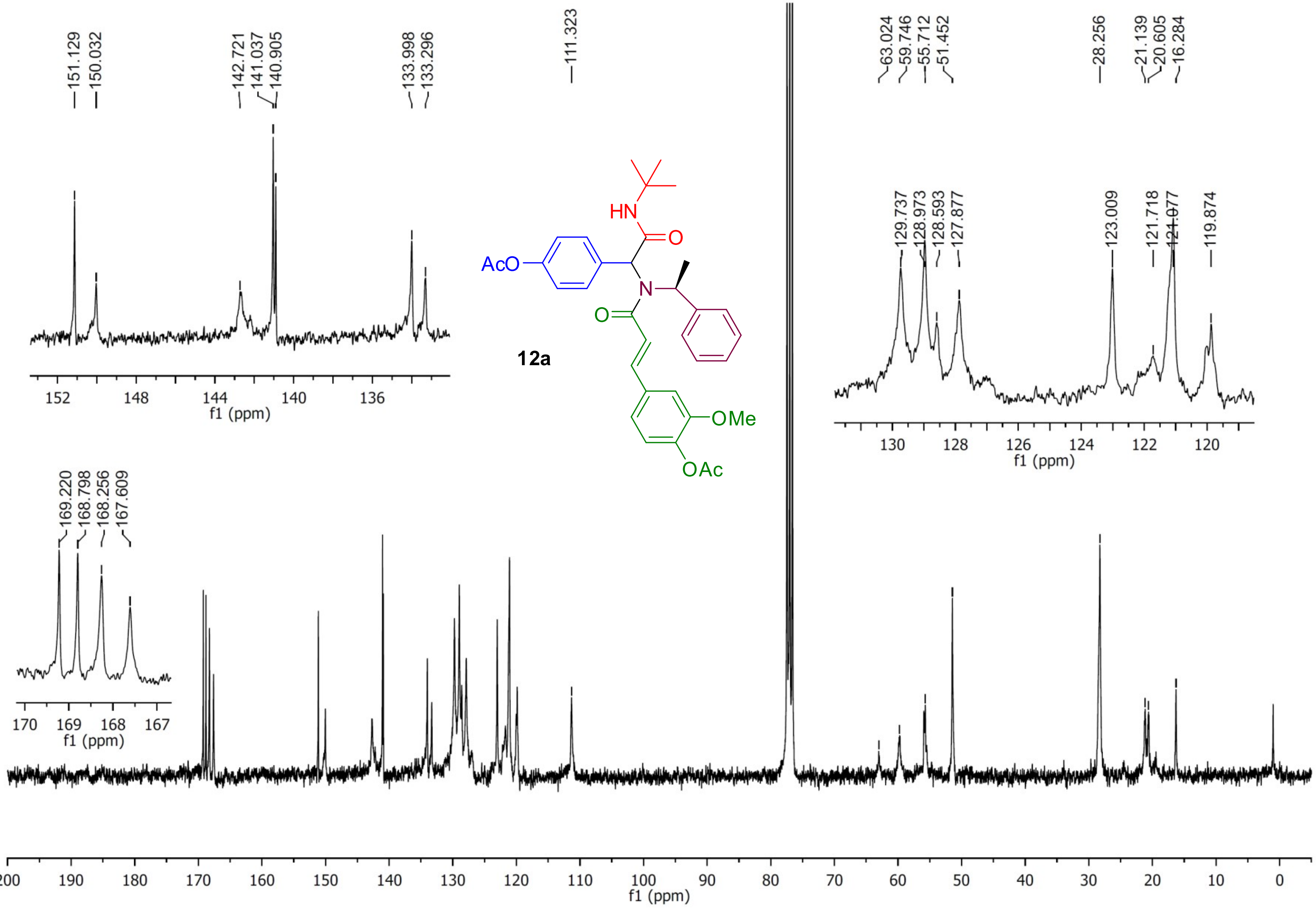
151.129
150.032
142.721
141.037
140.905
133.998
133.296
111.323
63.024
59.746
55.712
51.452
28.256
21.139
20.605
16.284
129.737
128.973
128.593
127.877
123.009
121.718
121.077
119.874
169.220
168.798
168.256
167.609
HN
O
AcO
N
O
OMe
OAc
12a
f1 (ppm)

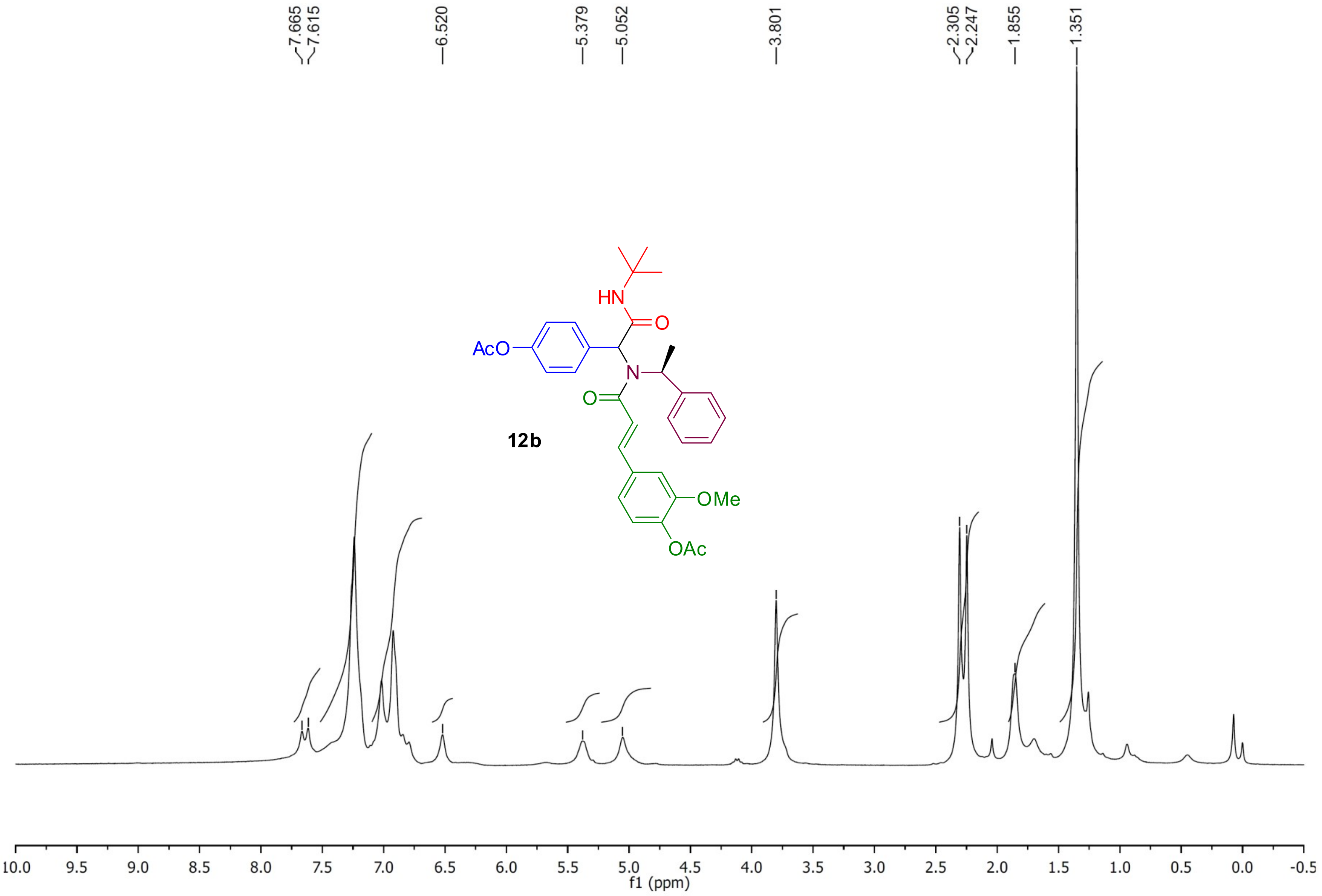
7.665
7.615
6.520
5.379
5.052
3.801
2.305
2.247
1.855
1.351
AcO
HN
O
N
O
OMe
OAc
12b
10.0 9.5 9.0 8.5 8.0 7.5 7.0 6.5 6.0 5.5 5.0 4.5 4.0 3.5 3.0 2.5 2.0 1.5 1.0 0.5 0.0 -0.5
f1 (ppm)

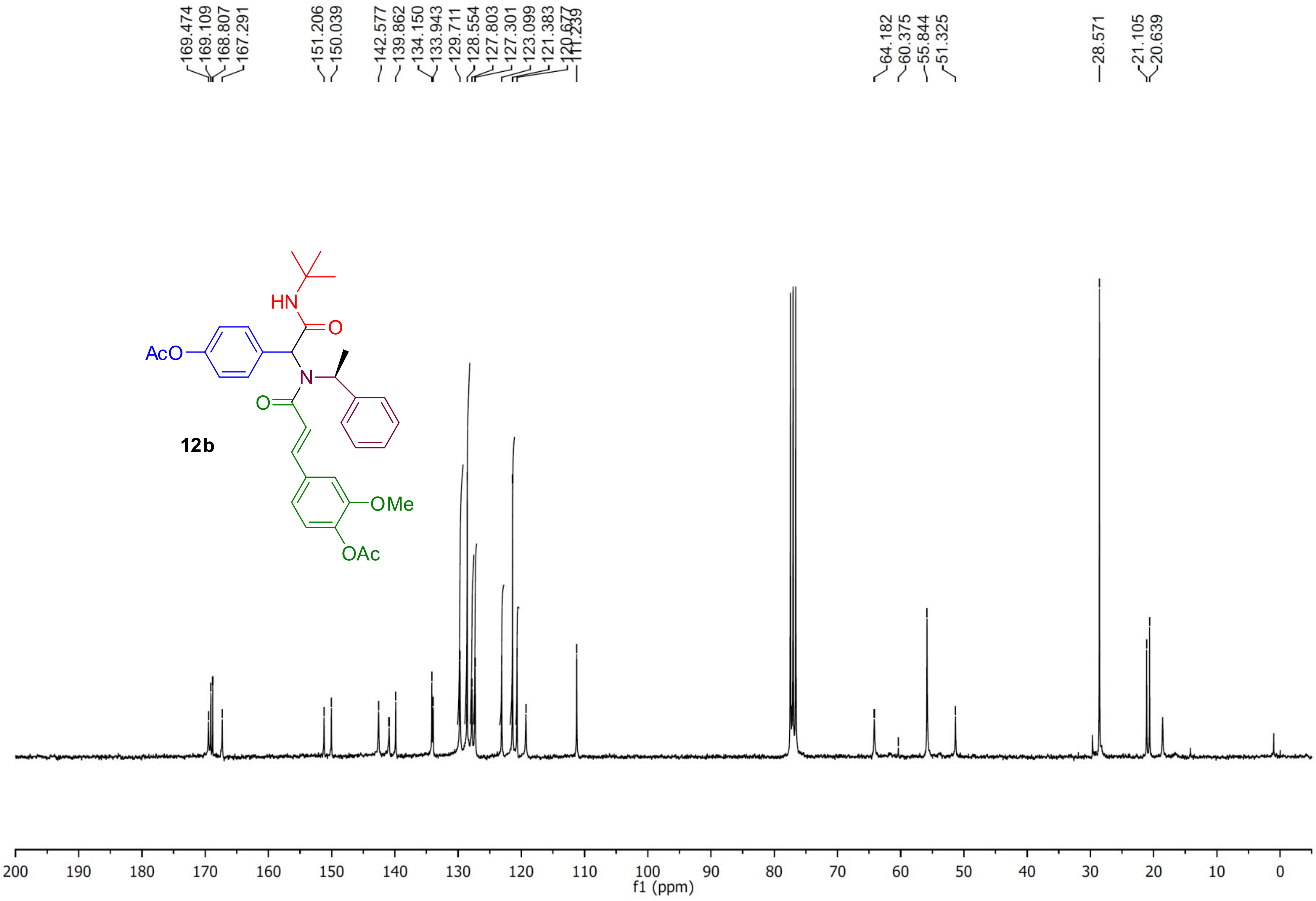
169.474
169.109
168.807
167.291
151.206
150.039
142.577
139.862
134.150
133.943
129.711
128.554
127.803
127.301
123.099
121.383
111.239
64.182
60.375
55.844
51.325
28.571
21.105
20.639
HN
O
AcO
N
O
OMe
OAc
12b
200 190 180 170 160 150 140 130 120 110 100 90 80 70 60 50 40 30 20 10 0
f1 (ppm)

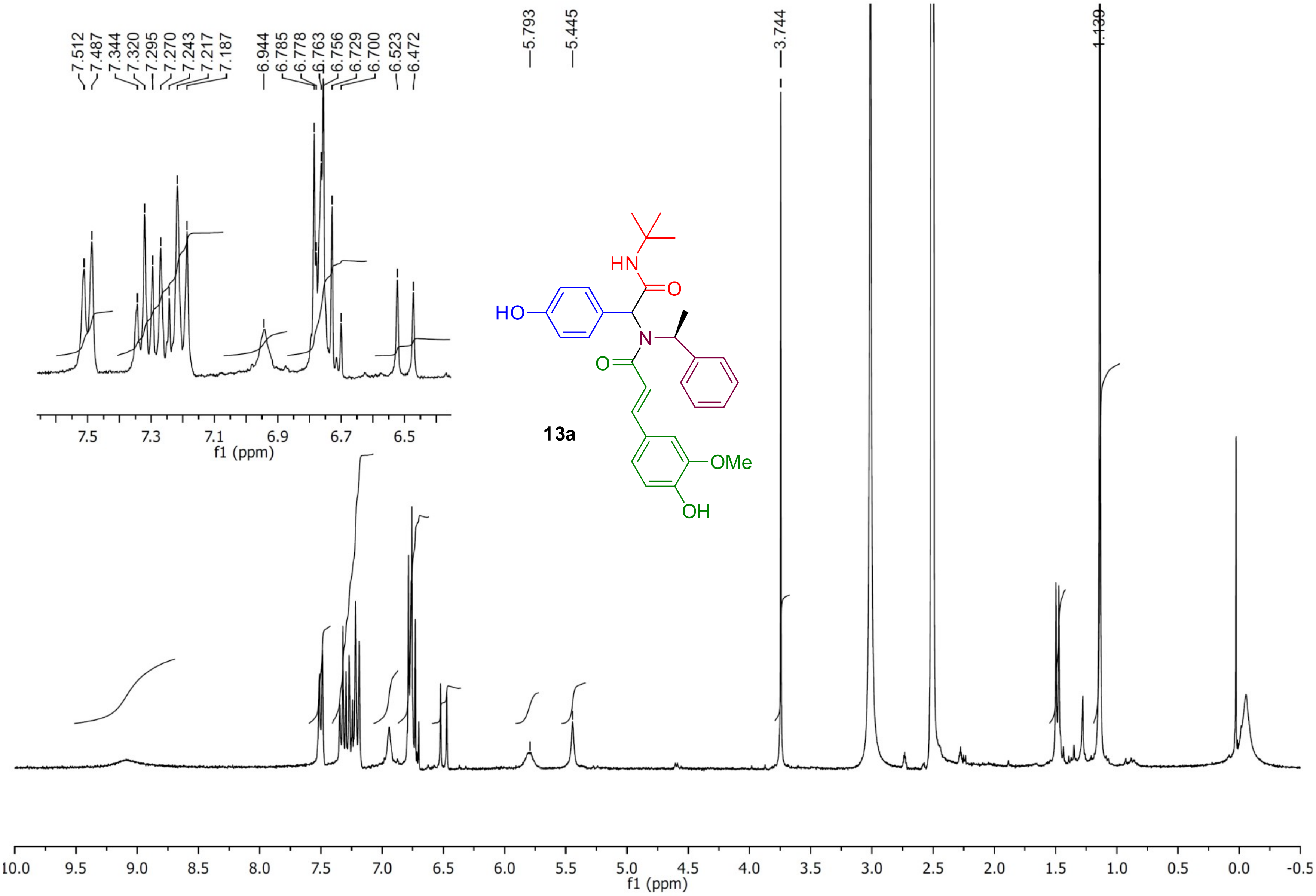
7.512
7.487
7.344
7.320
7.295
7.270
7.243
7.217
7.187
6.944
6.785
6.778
6.763
6.756
6.729
6.700
6.523
6.472
5.793
5.445
3.744
1.139
HN
O
HO
N
O
OMe
OH
13a
f1 (ppm)

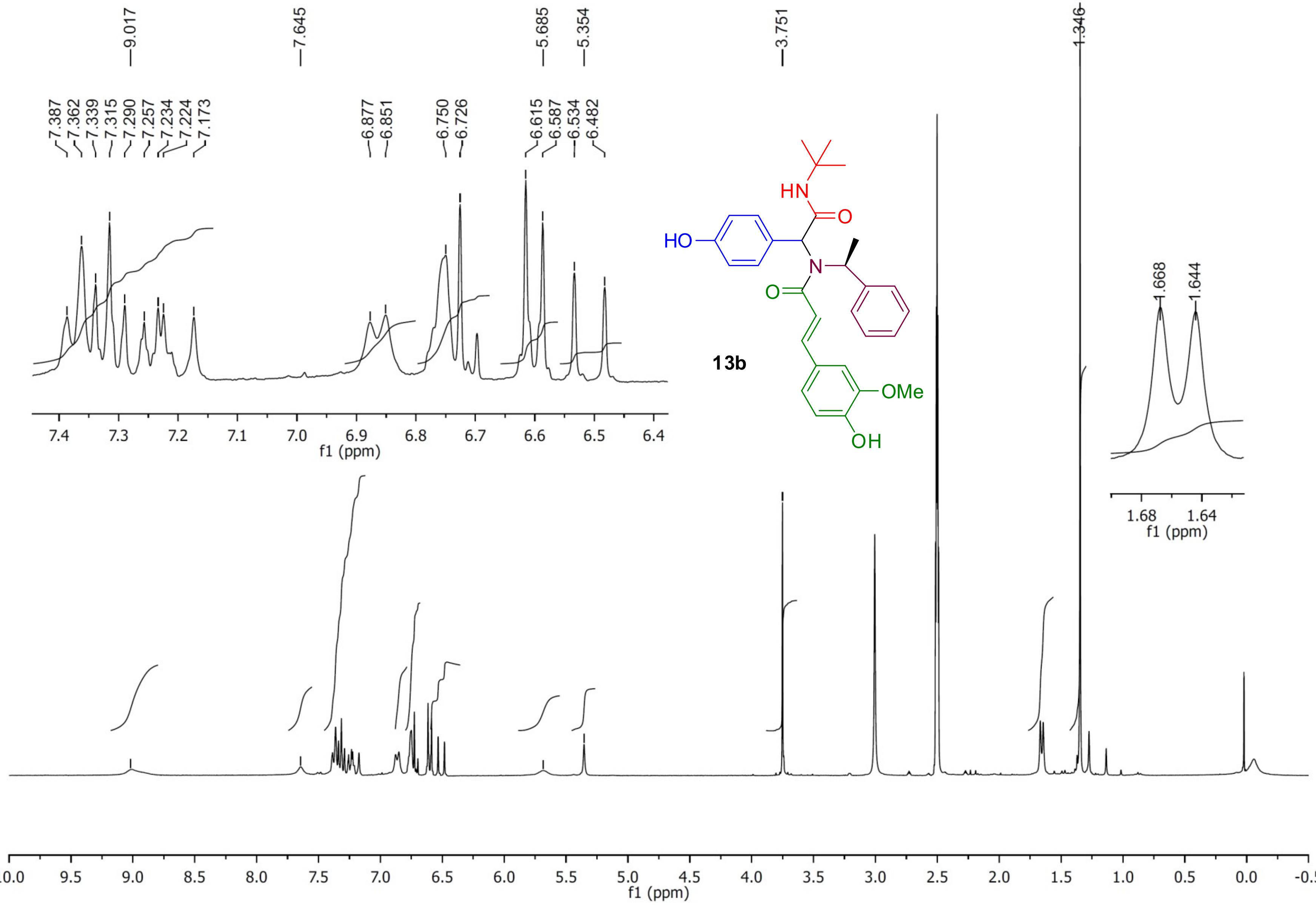
9.017
7.645
5.685
5.354
3.751
1.346
7.387
7.362
7.339
7.315
7.290
7.257
7.234
7.224
7.173
6.877
6.851
6.750
6.726
6.615
6.587
6.534
6.482
HN
O
HO
N
O
13b
OMe
OH
1.668
1.644
f1 (ppm)
f1 (ppm)
f1 (ppm)

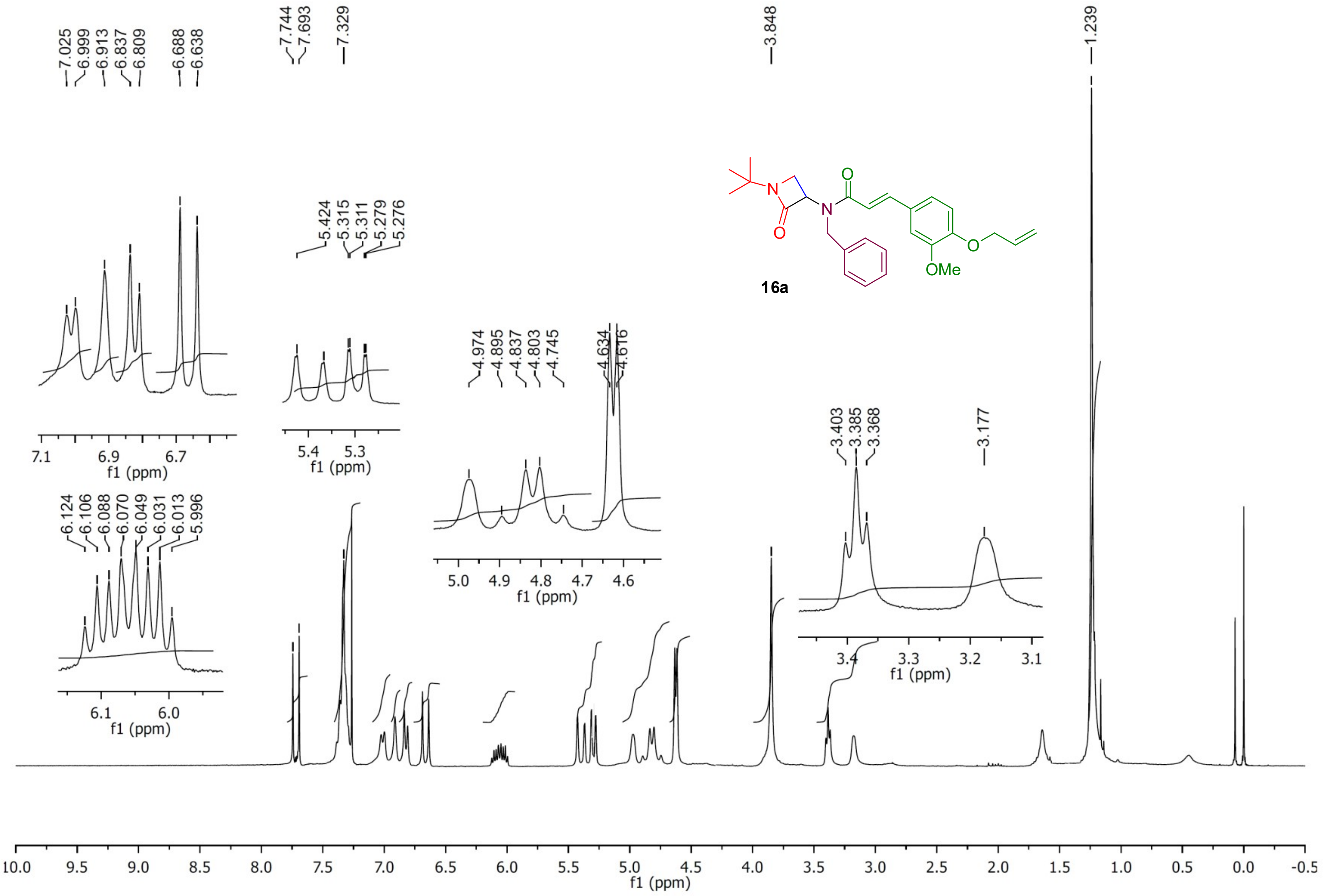

16a
OMe

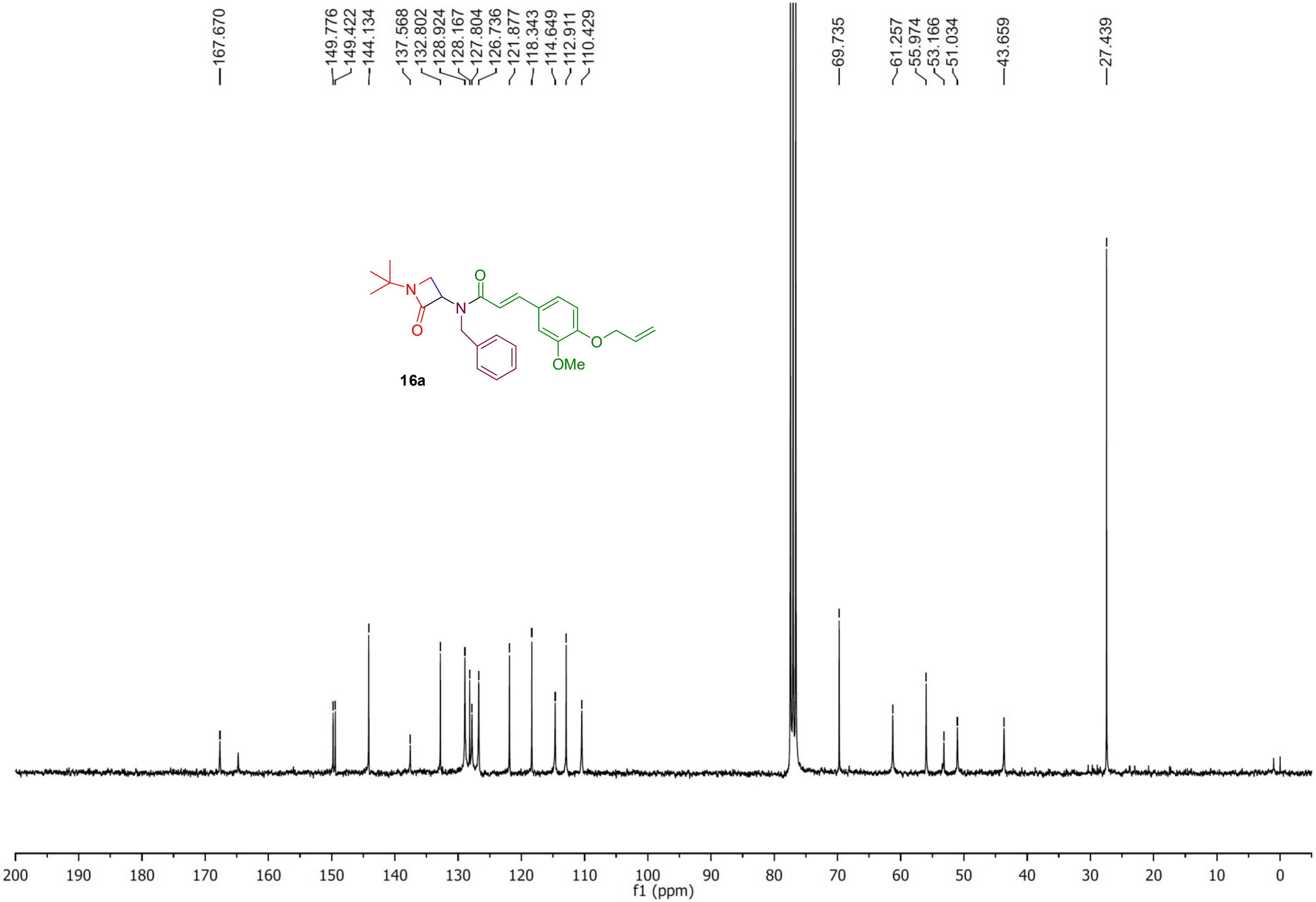

167.670
149.776
149.422
144.134
137.568
132.802
128.924
128.167
127.804
126.736
121.877
118.343
114.649
112.911
110.429
69.735
61.257
55.974
53.166
51.034
43.659
27.439
O
N
N
O
O
OMe
200
190
180
170
160
150
140
130
120
110
100
90
80
70
60
50
40
30
20
10
0
f1 (ppm)

16a

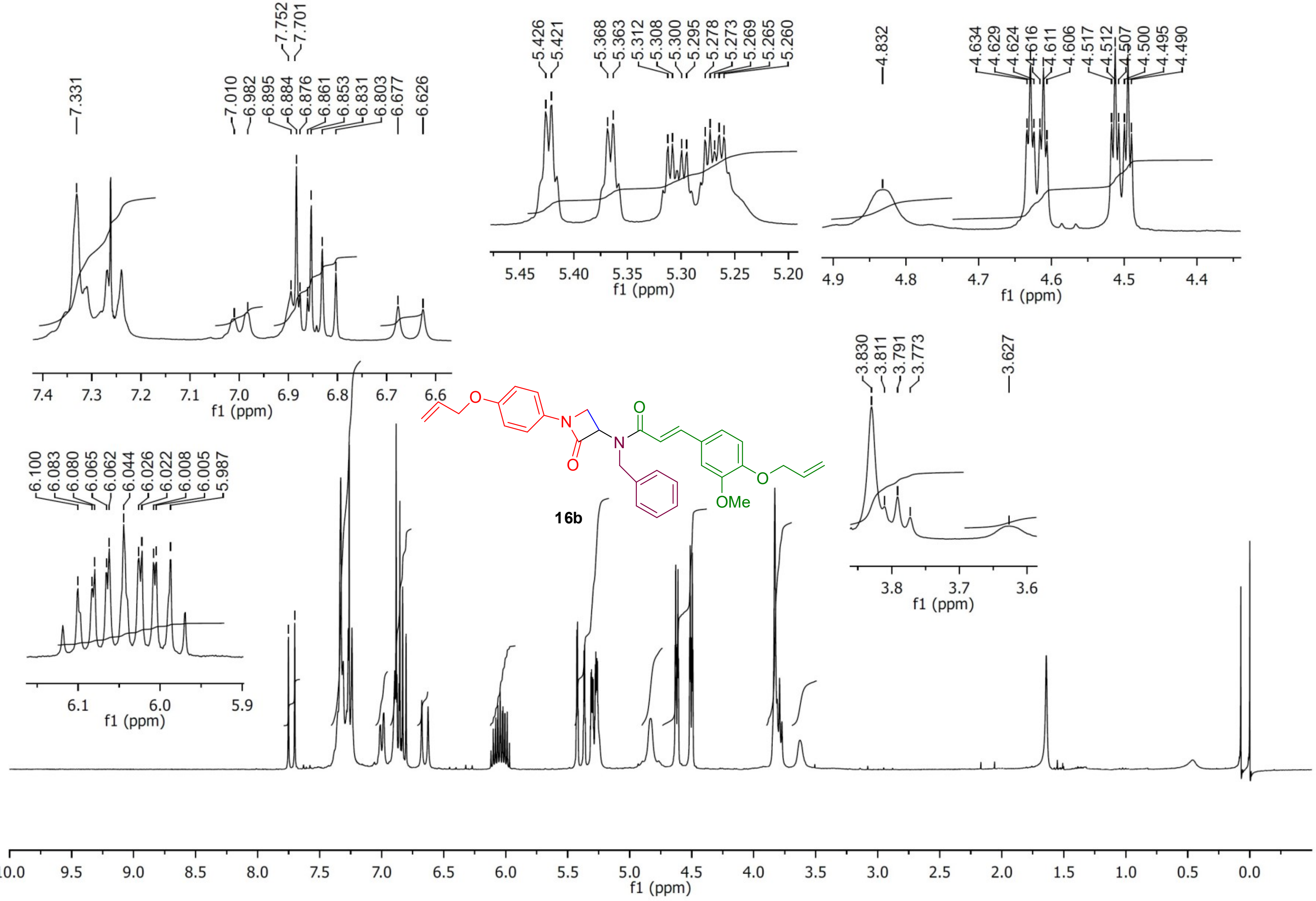
7.331
7.752
7.701
7.010
6.982
6.895
6.884
6.876
6.861
6.853
6.831
6.803
6.677
6.626
5.426
5.421
5.368
5.363
5.312
5.308
5.300
5.295
5.278
5.273
5.269
5.265
5.260
4.832
4.634
4.629
4.624
4.616
4.611
4.606
4.517
4.512
4.507
4.500
4.495
4.490
6.100
6.083
6.080
6.065
6.062
6.044
6.026
6.022
6.008
6.005
5.987
3.830
3.811
3.791
3.773
3.627
f1 (ppm)
O
N
OMe
16b

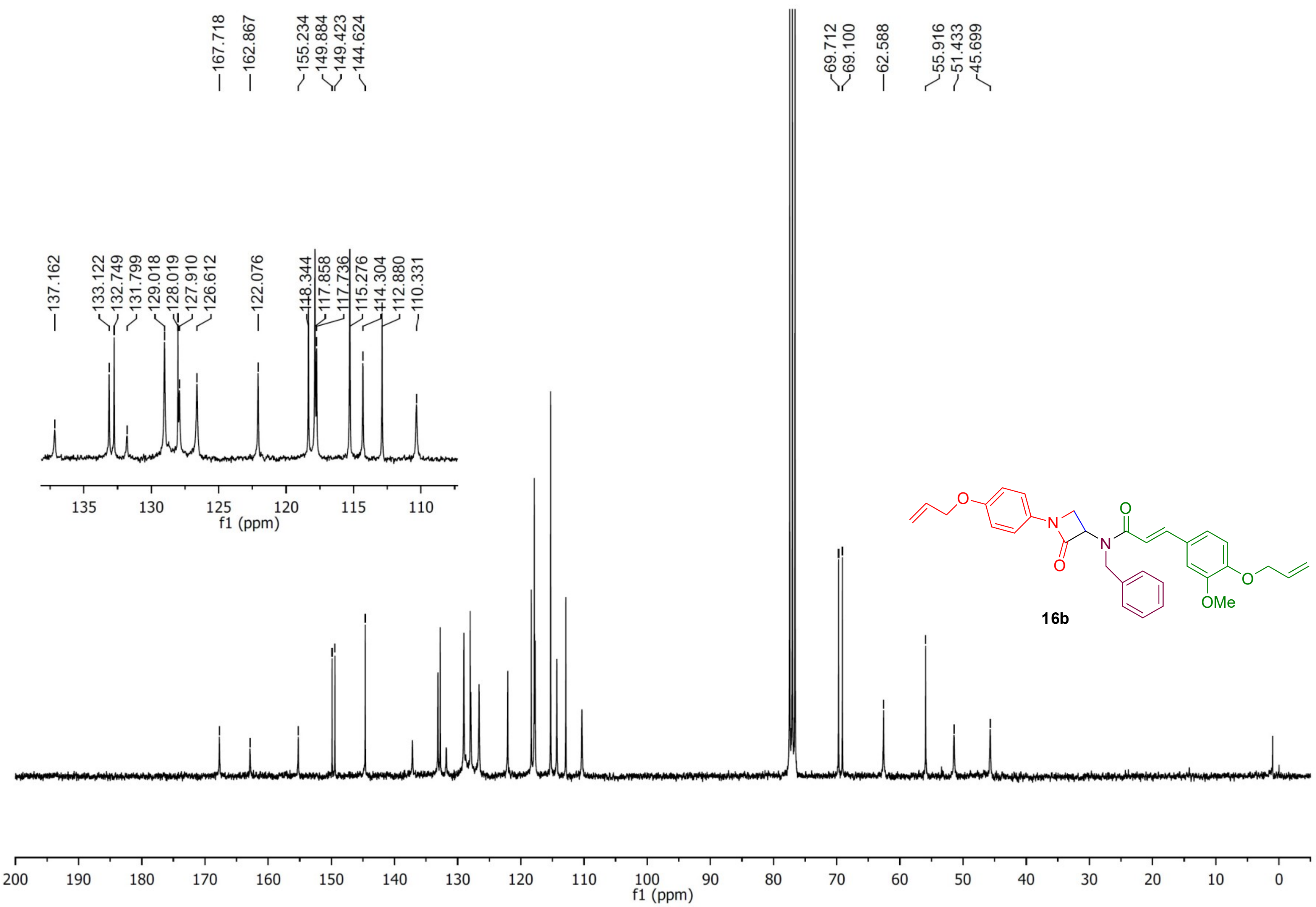
167.718
162.867
155.234
149.884
149.423
144.624
69.712
69.100
62.588
55.916
51.433
45.699
137.162
133.122
132.749
131.799
129.018
128.019
127.910
126.612
122.076
118.344
117.858
117.736
115.276
114.304
112.880
110.331
135
130
125
120
115
110
f1 (ppm)
O
N
O
N
O
O
OMe
16b
200
190
180
170
160
150
140
130
120
110
100
90
80
70
60
50
40
30
20
10
0
f1 (ppm)

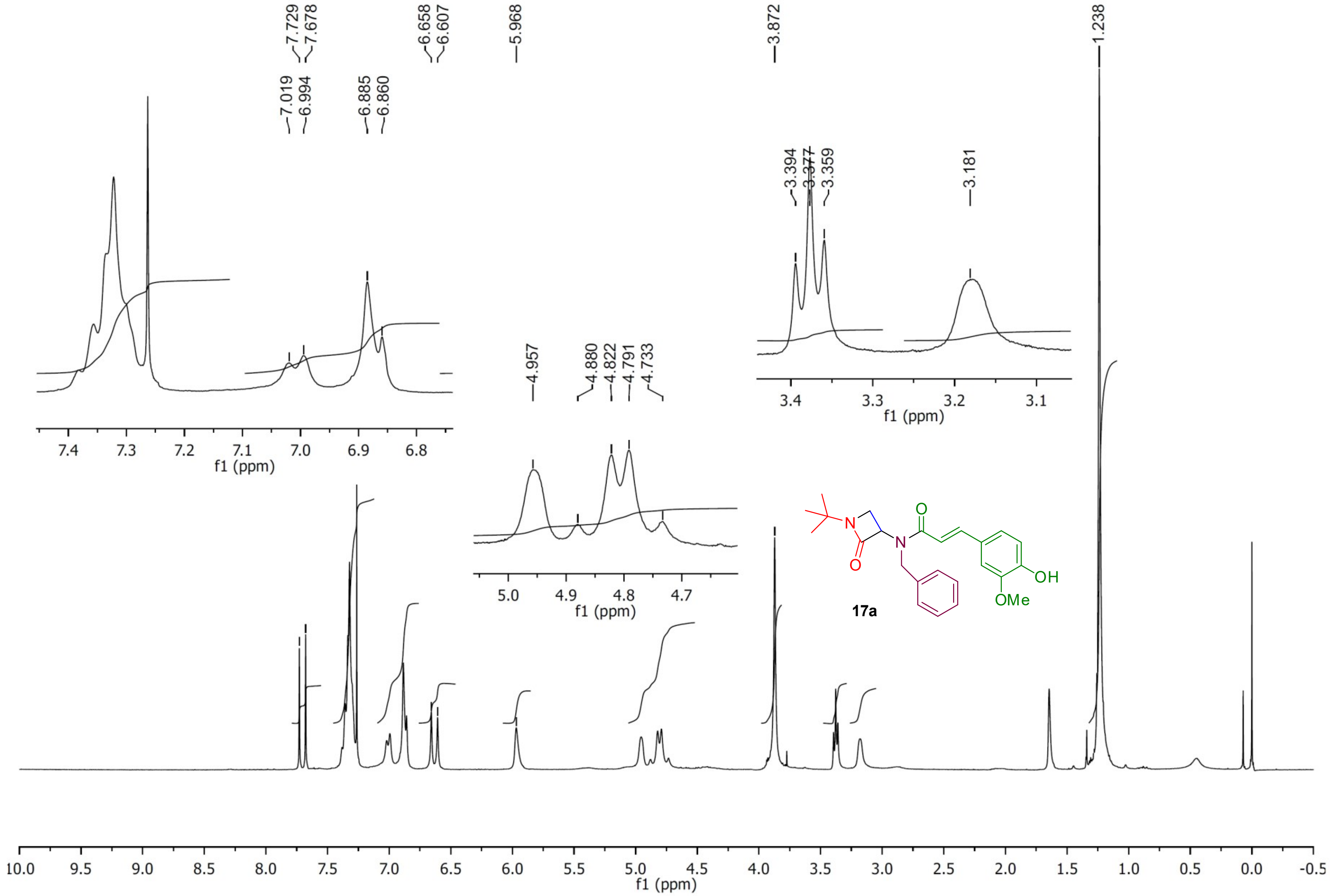
7.729
7.678
7.019
6.994
6.885
6.860
6.658
6.607
5.968
3.872
1.238
4.957
4.880
4.822
4.791
4.733
3.394
3.377
3.359
3.181
f1 (ppm)
N
O
OH
OMe
17a

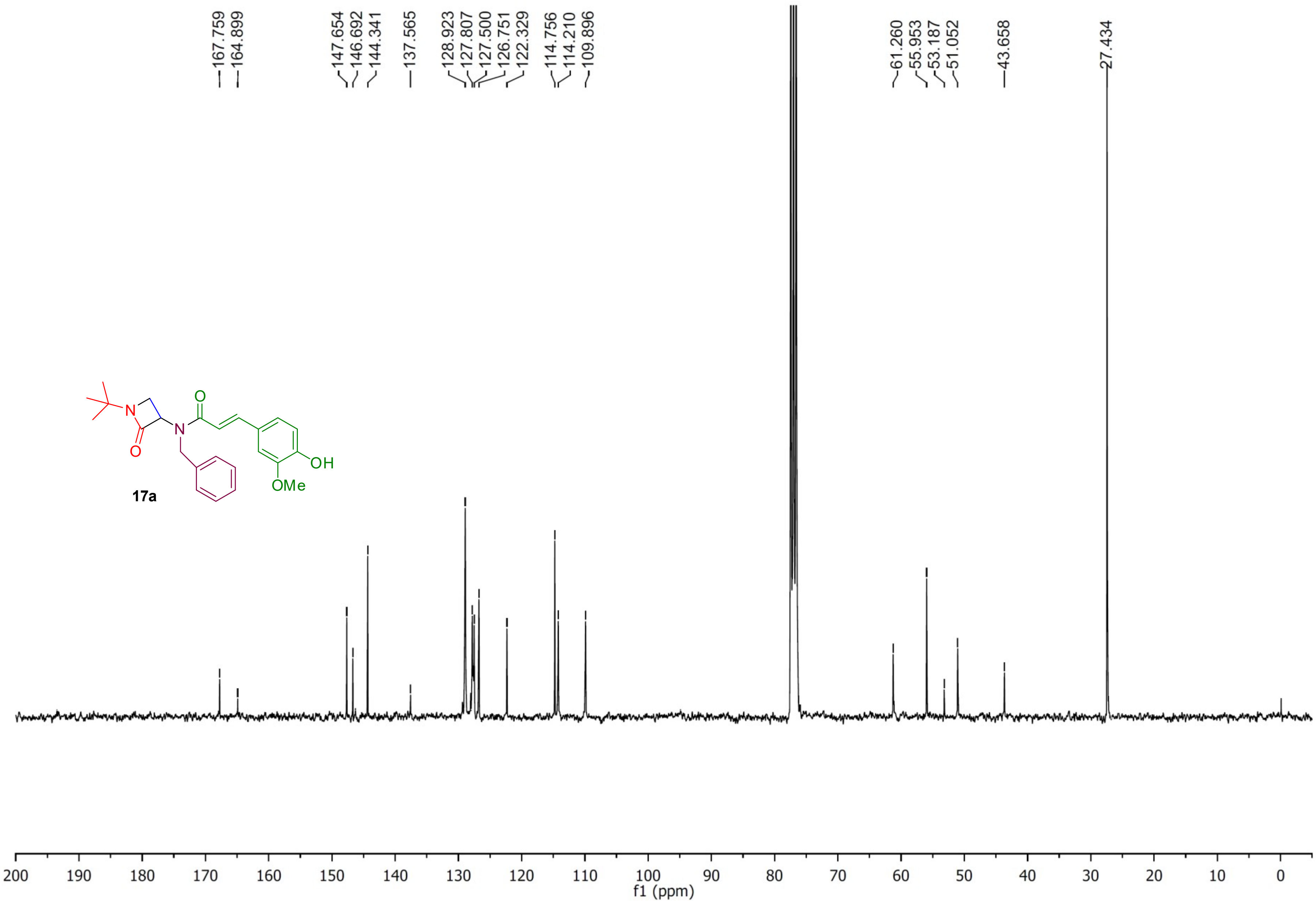
167.759
164.899
147.654
146.692
144.341
137.565
128.923
127.807
127.500
126.751
122.329
114.756
114.210
109.896
61.260
55.953
53.187
51.052
43.658
27.434
N
O
OH
OMe
17a
200 190 180 170 160 150 140 130 120 110 100 90 80 70 60 50 40 30 20 10 0
f1 (ppm)

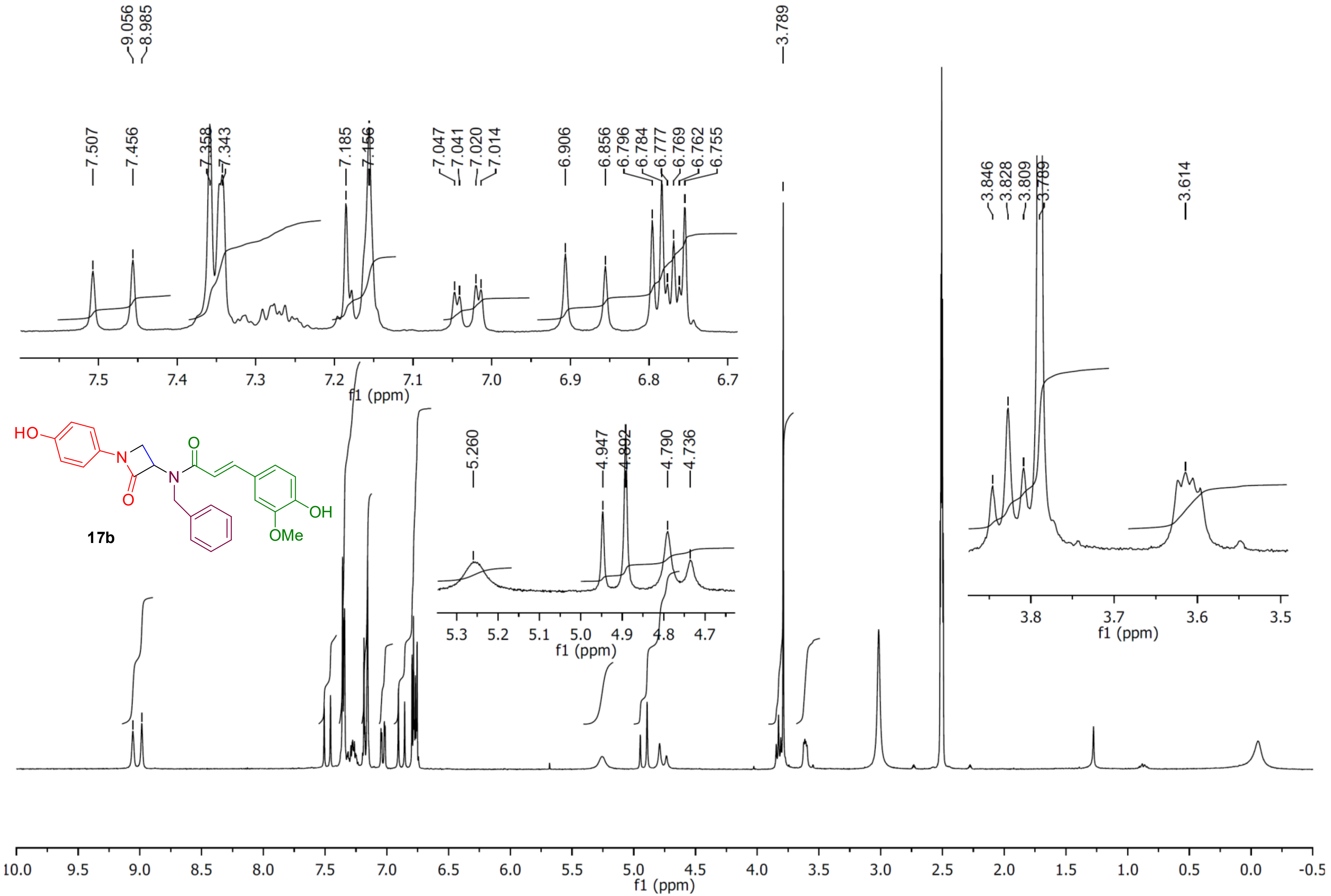

17b

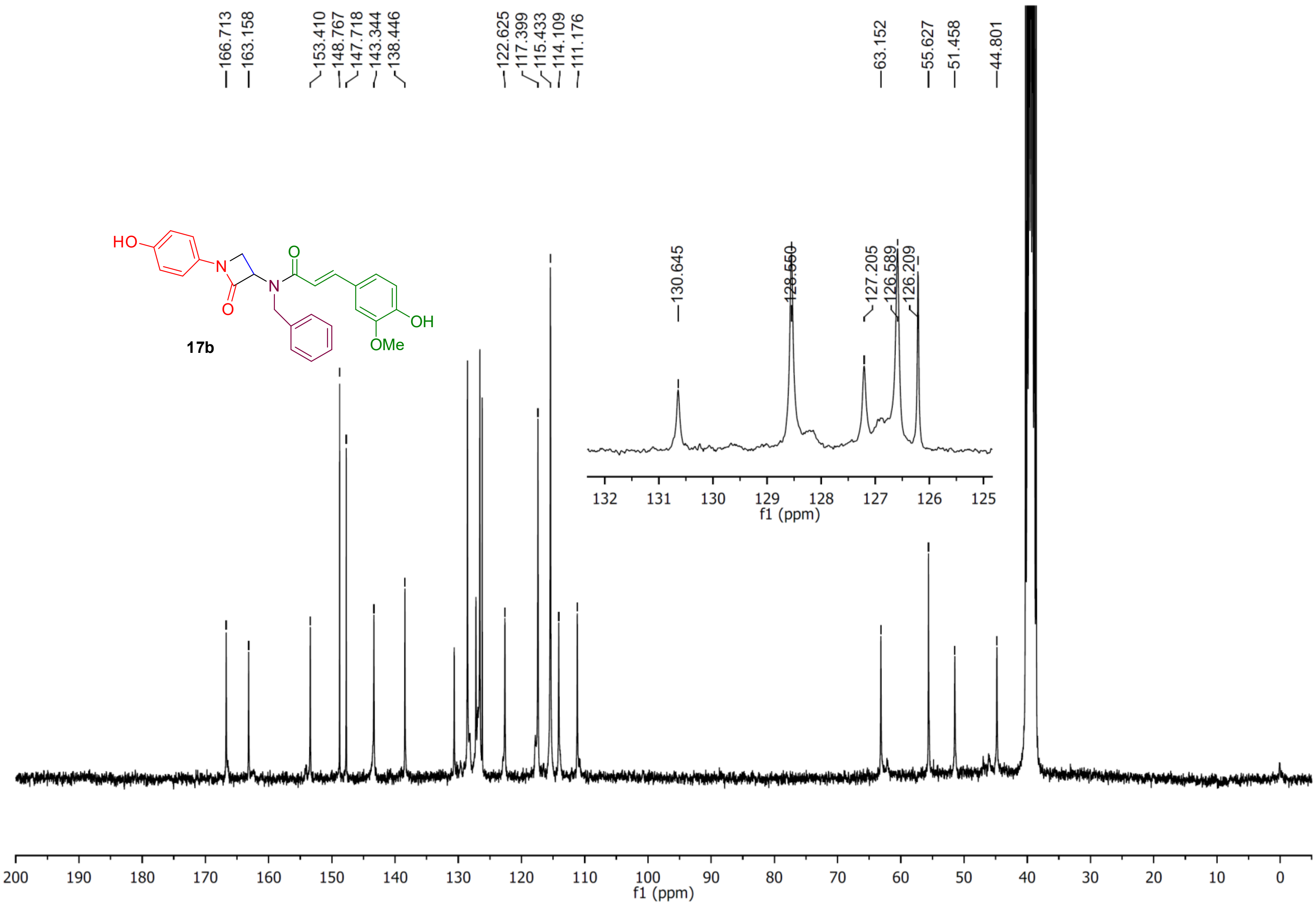
166.713
163.158
153.410
148.767
147.718
143.344
138.446
122.625
117.399
115.433
114.109
111.176
63.152
55.627
51.458
44.801
130.645
128.550
127.205
126.589
126.209
HO
N
O
N
O
OH
OMe
17b
f1 (ppm)
f1 (ppm)
